# Progesterone modulates the immune microenvironment to suppress ovalbumin-induced airway inflammation by inhibiting NETosis

**DOI:** 10.1038/s41598-024-66439-6

**Published:** 2024-07-26

**Authors:** Lin Wang, Feng-Ying Huang, Shu-Zhen Dai, Yongshu Fu, Xiangdong Zhou, Cai-Chun Wang, Guang-Hong Tan, Qi Li

**Affiliations:** 1 Department of Respiratory Medicine, Hainan Province Clinical Medical Center of Respiratory Disease, The First Affiliated Hospital of Hainan Medical University, Haikou, 570102 China; 2 NHC Key Laboratory of Tropical Disease Control, School of Tropical Medicine, The Second Affiliated Hospital, Hainan Medical University, Haikou, 571199 Hainan China

**Keywords:** Progesterone, Asthma, Allergy, Inflammasome, Immune microenvironment, Drug discovery, Immunology, Diseases

## Abstract

Studies have demonstrated that prior to puberty, girls have a lower incidence and severity of asthma symptoms compared to boys. This study aimed to explore the role of progesterone (P4), a sex hormone, in reducing inflammation and altering the immune microenvironment in a mouse model of allergic asthma induced by OVA. Female BALB/c mice with or without ovariectomy to remove the influence of sex hormones were used for the investigations. Serum, bronchoalveolar lavage fluid (BALF), and lung tissue samples were collected for analysis. The results indicated that P4 treatment was effective in decreasing inflammation and mucus secretion in the lungs of OVA-induced allergic asthma mice. P4 treatment also reduced the influx of inflammatory cells into the BALF and increased the levels of Th1 and Th17 cytokines while decreasing the levels of Th2 and Treg cytokines in both BALF and lung microenvironment CD45^+^ T cells. Furthermore, P4 inhibited the infiltration of inflammatory cells into the lungs, suppressed NETosis, and reduced the number of pulmonary CD4^+^ T cells while increasing the number of regulatory T cells. The neutrophil elastase inhibitor GW311616A also suppressed airway inflammation and mucus production and modified the secretion of immune Th1, Th2, Th17, and Treg cytokines in lung CD45^+^ immune cells. These changes led to an alteration of the immunological milieu with increased Th1 and Th17 cells, accompanied by decreased Th2, Treg, and CD44^+^ T cells, similar to the effects of P4 treatment. Treatment with P4 inhibited NETosis by suppressing the p38 pathway activation, leading to reduced reactive oxygen species production. Moreover, P4 treatment hindered the release of double-stranded DNA during NETosis, thereby influencing the immune microenvironment in the lungs. These findings suggest that P4 treatment may be beneficial in reducing inflammation associated with allergic asthma by modulating the immune microenvironment. In conclusion, this research indicates the potential of P4 as a therapeutic agent for ameliorating inflammation in OVA-induced allergic asthma mice.

## Introduction

Asthma is a chronic inflammatory condition of the airways, characterized by an increased sensitivity to various triggers, difficulty in breathing, and coughing and wheezing^1–3^. It has been observed that there is a gender-based disparity in the prevalence and severity of asthma symptoms, with girls having a lower incidence and intensity of symptoms prior to puberty, and women experiencing more serious symptoms during their menstrual cycle and menopause^4–7^. This suggests that sex hormones may have an influence on this condition. Studies have indicated that hormone replacement therapy (HRT) can provide relief from asthma symptoms in postmenopausal women, implying that hormonal changes during menopause can worsen asthma symptoms, whereas HRT can be beneficial^8^. Moreover, recent research has uncovered a complex relationship between neutrophil death and the inflammation associated with asthma. This process, known as NETosis, involves the release of extracellular traps (NETs) composed of DNA, histones, and antimicrobial peptides, which can activate the immune system and attract other immune cells, thus escalating airway inflammation^9,10^. Additionally, neutrophils can also promote inflammation by releasing pro-inflammatory molecules such as cytokines and chemokines, intensifying airway inflammation even further^11^. Furthermore, elevated levels of inflammatory mediators related to airway inflammation, such as IL-4 and IL-13, may affect the activity and survival of neutrophils, leading to an imbalance between neutrophil death and survival^12–14^. Understanding the interaction between neutrophil death and airway inflammation may be instrumental in the development of novel treatments for this persistent respiratory condition.

It is widely accepted within the scientific community that an imbalance between Th1/Th2 and Treg/Th17 cells in the pulmonary immune microenvironment can exacerbate the symptoms of asthma^15^. Th2 cells are known to suppress the production of cytokines by Th1 and natural killer cells, while Th1 cells impede the proliferation and maturation of mast cells, basophils, and eosinophils. It has been suggested that regulating the balance between these cell types, which are regulated by the cytokines produced by Th2 cells^16^, could be a potential therapeutic approach for treating allergic asthma.

Progesterone (P4) is a steroid hormone that plays a significant role in regulating various reproductive processes in females. Its effects are mediated by its interaction with different cell types, including both immune and non-immune cells^17^. Studies have demonstrated that P4 can reduce inflammation, activate dendritic cells and macrophages, and increase the presence of Tregs in both mouse models and human cord blood cells^18^. Additionally, recent evidence suggests that P4 may modulate the innate immune response to SARS-CoV-2 and potentially reduce the severity of SARS-CoV-2-induced pneumonia^19,20^. Furthermore, P4 has been observed to influence the frequency of ciliary pulsation in the airway epithelium through its impact on the expression of P4 receptors^17^. Additionally, P4 has been found to stimulate the differentiation of T cells into Tregs while inhibiting their differentiation into Th17 cells, allowing it to suppress the inflammatory response^21,22^. Therefore, a hypothesis was proposed that P4 may be able to inhibit allergic airway inflammation by influencing the immune microenvironment and NETosis in asthmatic lungs. To test this hypothesis, a female mouse model of allergic airway inflammation was established by sensitizing mice to ovalbumin (OVA) and subjecting them to inhalation challenges with OVA. Additionally, some mice underwent ovariectomy (OE) to examine the effect of hormonal changes on the immune microenvironment. Subsequently, the efficacy of P4 treatment on airway inflammation and the immune microenvironment associated with NETosis in the allergic lungs was evaluated.

## Results

### P4 treatment relieves symptoms and reduces lung inflammation in OVA-mediated allergic mice

**Figure 1:**
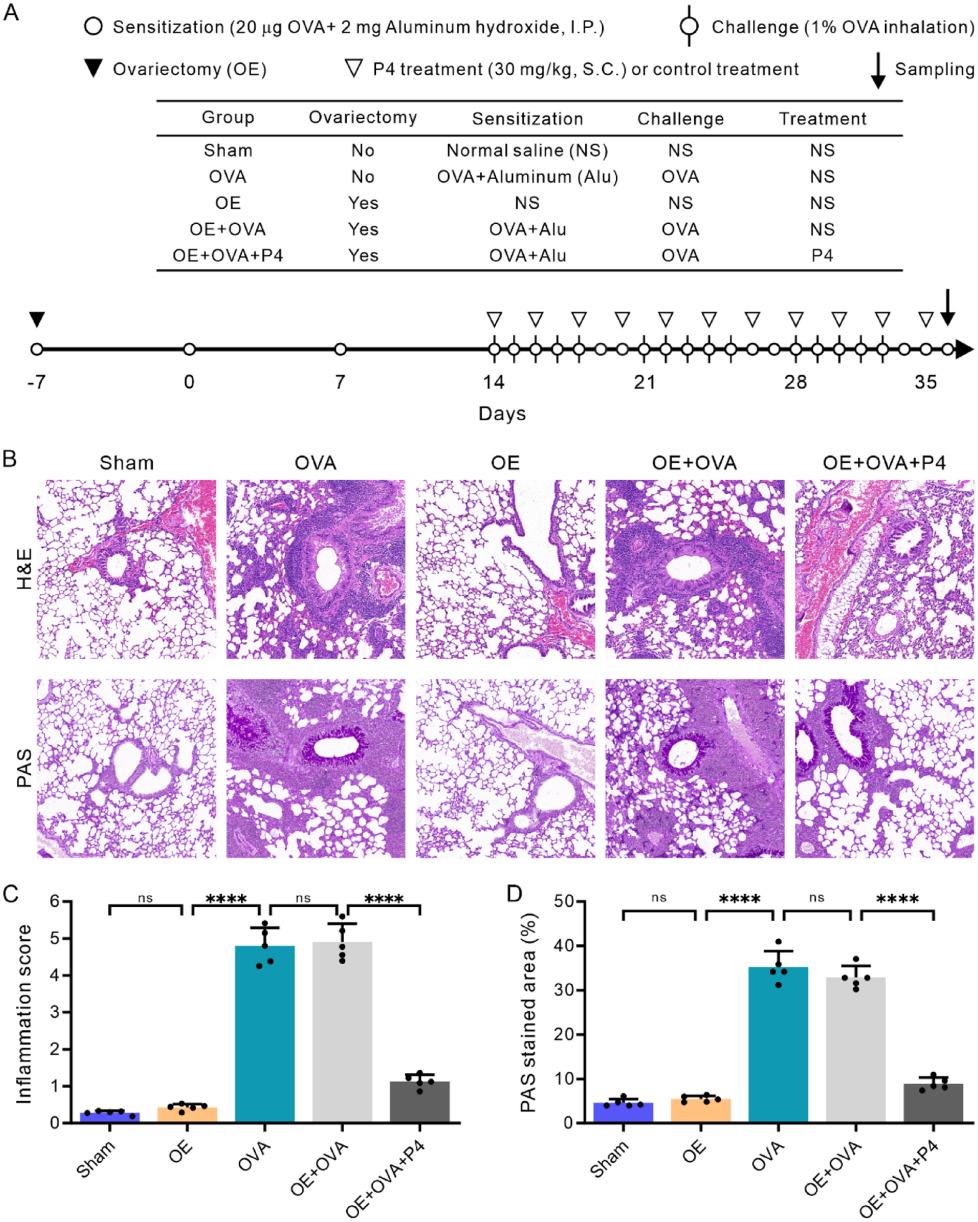
P4 treatment decreases airway inflammation as observed through lung histology. **(A)** The diagram presents the creation of the OVA-induced allergic asthma model and the procedure for sensitization and challenge with OVA, P4 treatment, and ovariectomy. **(B–D)** Lung sections (n = 5) were stained using H&E or PAS stains (**B**), and subsequently, the inflammation scores (**C**) and the percentage of the PAS-stained area (**D**) were determined based on the respective stains used. The experiments utilized two batches of mice, with data collected from the first batch. The number of experimental mice or samples is represented by the number of dots in each bar. Results are displayed as the mean ± SD. They were then analyzed using one-way ANOVA followed by Tukey's post-hoc test. Statistical significance is represented as *P < 0.05, **P < 0.01, ***P < 0.001, and ****P < 0.0001.

Our study demonstrated that mice exposed to a 1% OVA challenge began to display asthma-related symptoms such as sneezing, nasal itching, difficulty breathing, and cyanosis from day 3, and these symptoms became more severe with continued inhalation of 1% OVA. In contrast, the Sham and OE groups did not show any considerable symptoms. However, the OE + OVA + P4 group exhibited a considerable decrease in these symptoms, similar to those observed in the Sham and OE groups. Histological analysis of the lungs revealed that the OVA and OE + OVA groups had an increase in inflammatory cell infiltration, mucus production, and damage to airway epithelial cells (Fig. 1B). Nevertheless, P4 treatment in the OE + OVA + P4 group resulted in a significant reduction of these pathological changes (Fig. 1B). Additionally, airway inflammation scores and periodic acid-Schiff (PAS) staining to measure mucus production were used to further evaluate the efficacy of the treatments. Our findings showed that both the OVA and OE + OVA groups had significantly higher airway inflammation scores (Fig. 1C) and PAS staining areas (Fig. 1D) compared to the Sham and OE groups (P < 0.0001). However, P4 administration resulted in a remarkable decrease in these scores (Fig. 1C) and PAS staining areas (Fig. 1D), bringing them to levels similar to those observed in the Sham and OE groups. Consequently, these results indicate that P4 therapy is highly effective in reducing symptoms and signs of allergic asthma, as well as mitigating airway inflammation.

### P4 treatment decreases the number of inflammatory cells in the BALF

**Figure 2:**
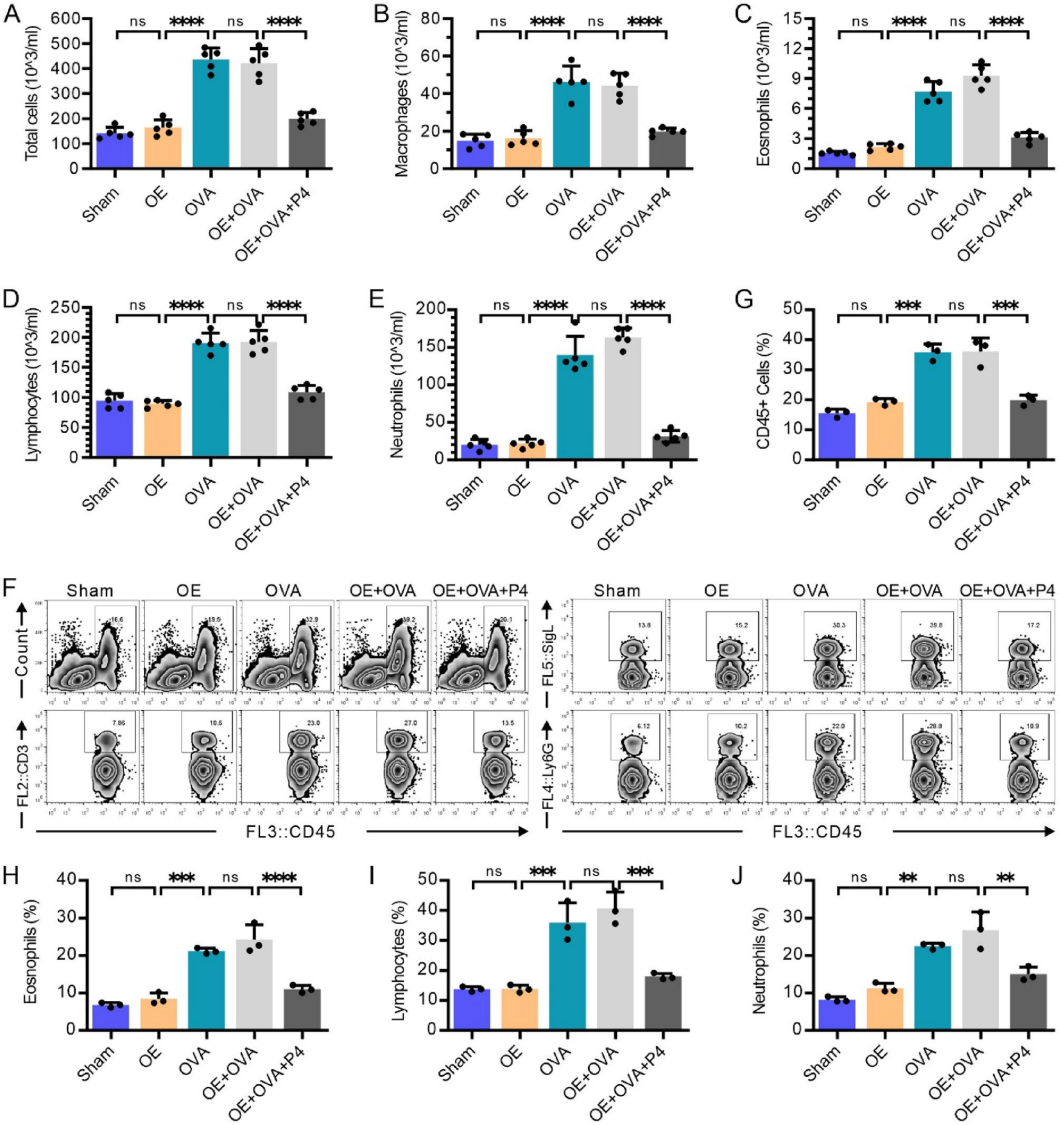
Treatment with P4 reduces the number of inflammatory cells found in the BALF. **(A–E)** The inflammatory cells were stained using Wright's-Giemsa and classified into subgroups such as total inflammatory cells (**A**), macrophages (**B**), eosinophils (**C**), lymphocytes (**D**), and neutrophils (**E**), showing the mean numbers in 5 high-power fields per group. **(F–J)** FCM analysis was conducted to examine the total cell count and classification of the inflammatory cells in the BALF (n = 3). This analysis included representative images (**F**) as well as CD45^+^ total inflammatory cells (**G**), CD45^+^SigL^+^ eosinophils (**H**), CD45^+^CD3^+^ lymphocytes (**I**), and CD45^+^Ly6G^+^ neutrophils (**J**). The experiments utilized two batches of mice, with data collected from the first batch. The number of experimental mice or samples is represented by the number of dots in each bar. Results are displayed as the mean ± SD. They were then analyzed using one-way ANOVA followed by Tukey's post-hoc test. Significance levels were indicated as *P < 0.05, **P < 0.01, ***P < 0.001, and ****P < 0.0001.

In this study, we employed Wright's Giemsa staining to label and classify the inflammatory cells found in BALF, including macrophages, eosinophils, lymphocytes, and neutrophils. The results revealed a notable increase in the overall count of inflammatory cells (Fig. 2A), macrophages (Fig. 2B), eosinophils (Fig. 2C), lymphocytes (Fig. 2D), and neutrophils (Fig. 2E) in both the groups exposed to OVA and OE + OVA, when compared to the control groups (Sham and OE) (p < 0.0001). Intriguingly, the number of these cells in the OE + OVA + P4 group was significantly lower than in the OVA and OE + OVA groups, and closely resembled the levels observed in the Sham and OE groups (p < 0.0001, Fig. 2A–E). Additionally, we utilized flow cytometry analysis to investigate the expression of CD45^+^ cells, which serves as a marker for total inflammatory cells. This analysis also enabled us to classify double-positive eosinophils (CD45^+^SigL^+^), lymphocytes (CD45^+^CD3^+^), and neutrophils (CD45^+^Ly6G^+^). The results obtained from flow cytometry (Fig. 2F–J) were consistent with those obtained from Wright's Giemsa staining, revealing significantly higher proportions of CD45^+^ total inflammatory cells (Fig. 2G), CD45^+^SigL^+^ double-positive eosinophils (Fig. 2H), CD45^+^CD3^+^ double-positive lymphocytes (Fig. 2I), and CD45^+^Ly6G^+^ double-positive neutrophils (Fig. 2J) in the OVA and OE + OVA groups compared to the Sham and OE groups. However, CD45^+^ total inflammatory cells (Fig. 2G) and their subtypes, including eosinophils (Fig. 2H), lymphocytes (Fig. 2I), and neutrophils (Fig. 2J), were significantly reduced in the OE + OVA + P4 group, which showed a similar percentage to the Sham and OE groups. These findings suggest that P4 treatment can effectively reduce the number of inflammatory cells in the airways of mice with allergic asthma.

### P4 treatment influences the concentrations of cytokines associated with Th1/Th2, Th17, and Tregs in the BALF

**Figure 3:**
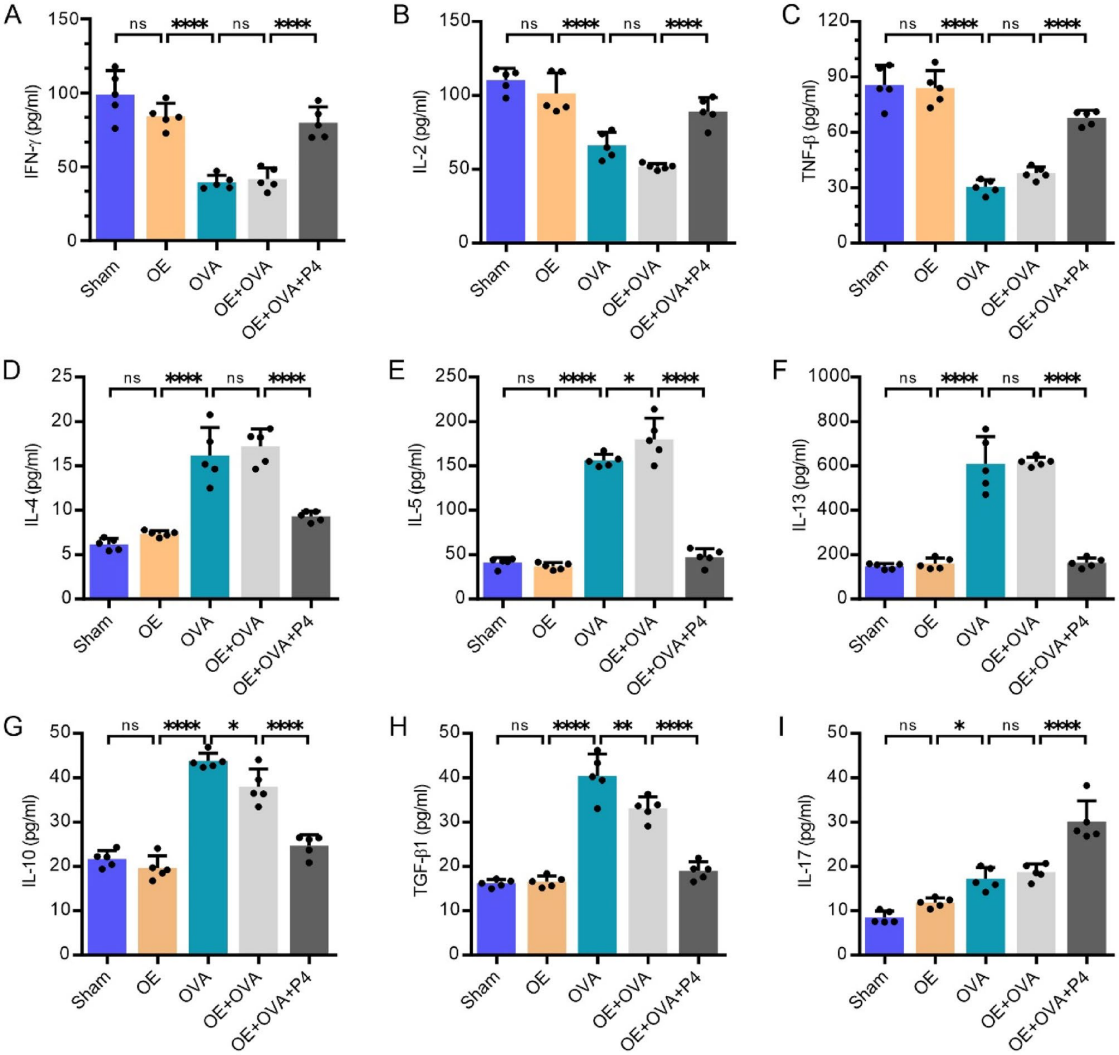
P4 treatment alters the secretion of Th1, Th2, Th17, and Treg cytokines in the BALF as determined by ELISA analysis. **(A–C)** The levels of the Th1 cytokines IFN-γ (**A**), IL-2 (**B**), and TNF-β (**C**). **(D–F)** The levels of the Th2 cytokines IL-4 (**D**), IL-5 (**E**), and IL-13 (**F**). **(G)** The level of the Th17 cytokine IL-17. **(H,I)** The levels of the Treg cytokines IL-10 (**H**) and TGF-β1 (**I**). The experiments utilized two batches of mice, with data collected from the first batch. The number of experimental mice or samples is represented by the number of dots in each bar. Results are displayed as the mean ± SD. Statistical analysis was performed using one-way ANOVA followed by Tukey's post-hoc test. Statistical significance is denoted as *P < 0.05, **P < 0.001, and ****P < 0.0001.

ELISA was used to quantify the levels of Th1 (IFN-γ, IL-2, and TNF-β), Th2 (IL-4, IL-5, and IL-13), Tregs (IL-10 and TGF-β1), and Th17 (IL-17) cytokines in BALF. The results showed that the OVA and OE + OVA groups displayed significantly lower levels of IFN-γ (Fig. 3A), IL-2 (Fig. 3B), and TNF-β (Fig. 3C) in comparison to the Sham and OE groups (P < 0.0001). In contrast, the concentrations of IL-4 (Fig. 3D), IL-5 (Fig. 3E), and IL-13 (Fig. 3F) were significantly higher in the OVA and OE + OVA groups than the Sham and OE groups (P < 0.0001). However, P4 treatment in the OE + OVA + P4 group caused a significant increase in the levels of IFN-γ (Fig. 3A), IL-2 (Fig. 3B), and TNF-β (Fig. 3C), while significantly decreasing the levels of IL-4 (Fig. 3D), IL-5 (Fig. 3E), and IL-13 (Fig. 3F). These findings suggest that P4 has a regulatory effect on the dominant Th2 cytokines, shifting them towards Th1 cytokines in mice with OVA-induced allergic inflammation.

In addition, the concentrations of Treg-associated cytokines IL-10 (Fig. 3G) and TGF-β1 (Fig. 3H) were higher in both the OVA and OE + OVA groups compared to the Sham and OE groups. Nevertheless, P4 treatment in the OE + OVA + P4 group reversed the levels of IL-10 (Fig. 3G) and TGF-β1 (Fig. 3H) to a similar degree as those seen in the Sham and OE groups. On the other hand, the concentrations of IL-17 in the OVA and OE + OVA groups were higher than those in the Sham or OE groups, and P4 treatment in the OE + OVA + P4 group further increased the IL-17 level (Fig. 3I). These findings indicate that P4 has the ability to modify the balance between Treg and Th17 cells in mice with OVA-induced allergic asthma.

### P4 treatment modifies the cellular production of cytokines related to Th1, Th2, Th17, and Tregs in the lungs

**Figure 4:**
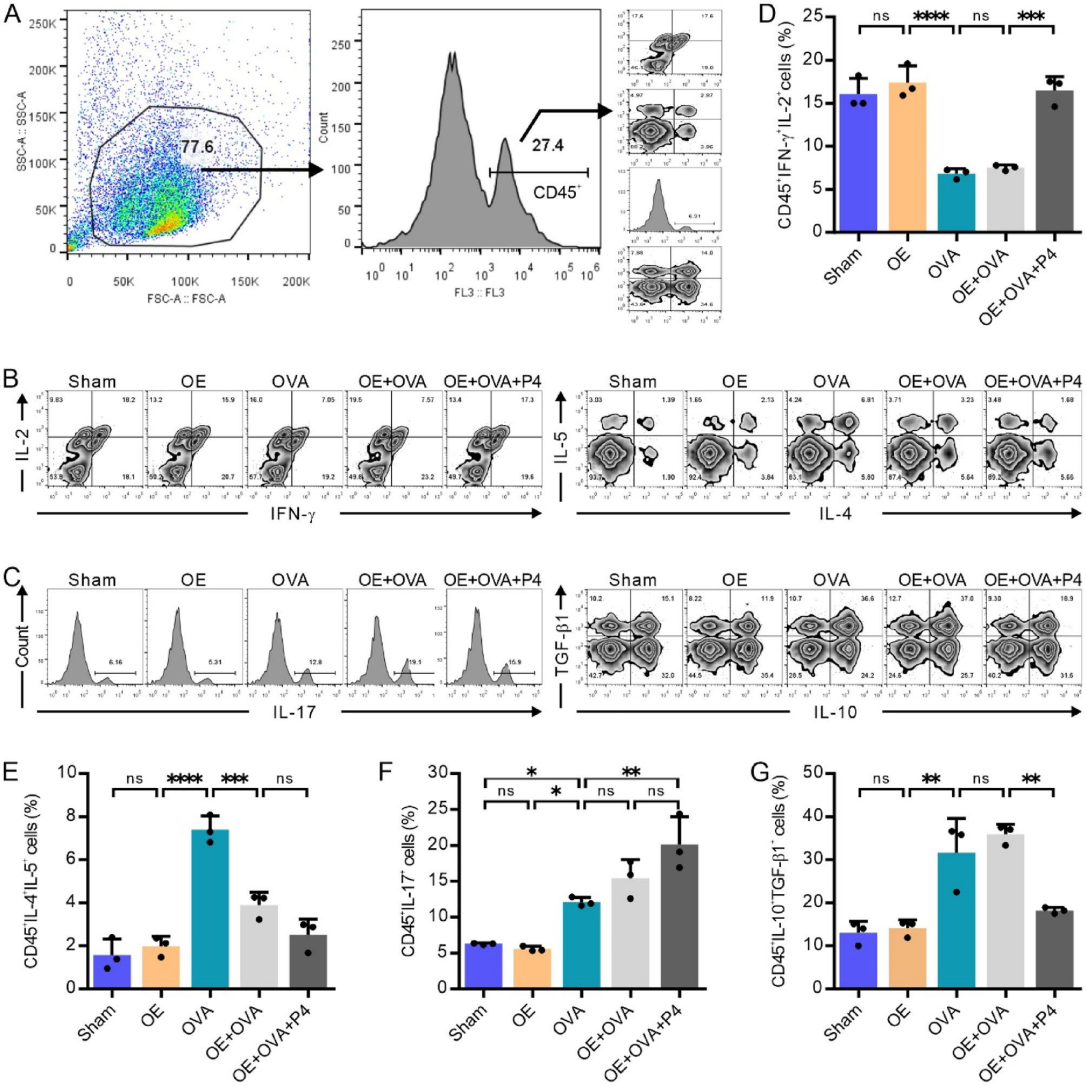
P4 treatment affects the number of CD45 + immune cells producing Th1, Th2, Th17, and Treg cytokines in lung tissues, as determined by FCM. **(A)** The gating strategy used to analyze the percentages of CD45^+^ immune cells producing Th1, Th2, Th17, and Treg cytokines. **(B,C)** Representative FCM images showing CD45^+^ cells producing Th1 cytokines IFN-γ and IL-2 (**B**, left panel), Th2 cytokines IL-4 and IL-5 (**B**, right panel), Th17 cytokine IL-17 (**C**, left panel), and Treg cytokines IL-10 and TGF-β1 (**C**, right panel). **(D–G)** Quantification of three FCM experiments measuring the production of Th1 (**D**), Th2 (**E**), Th17 (**F**), and Treg (**G**) cytokines by CD45^+^ cells. The experiments utilized two batches of mice, with data collected from the first batch. The number of experimental mice or samples is represented by the number of dots in each bar. Results are displayed as the mean ± SD. The statistical analysis was conducted using one-way ANOVA followed by Tukey's post-hoc test. Statistical significance is indicated as *P < 0.05, **P < 0.001, and ****P < 0.0001.

Flow cytometry (FCM) was employed to isolate CD45-positive cells, which were then used to measure the presence of cells secreting cytokines associated with Th1, Th2, Th17, and Treg cells (Fig. 4A). Results showed that the proportion of CD45^+^IFN-γ^+^IL-2^+^ Th1 cells was higher in the Sham and OE groups than in the OVA and OE + OVA groups (Fig. 4B, D). However, P4 treatment decreased the percentage of CD45^+^IFN-γ^+^IL-2^+^ Th1 cells to a level similar to that of the Sham and OE groups (Fig. 4B, D). In contrast, the proportion of CD45^+^IL-4^+^IL-5^+^ Th2 cells was lower in the Sham and OE groups than in the OVA and OE + OVA groups, and P4 treatment significantly reduced these cells to the same level as that of the Sham and OE groups (Fig. 4B, E). Additionally, the percentage of CD45^+^IL-17^+^ Th17 cells was higher in the OVA and OE groups compared to the Sham and OE groups, and P4 treatment further increased the percentage of CD45^+^IL-17^+^ Th17 cells (Fig. 4C, F). The percentage of CD45^+^IL-10^+^TGF-β1^+^ Treg cells was significantly higher in the OVA and OE + OVA groups than in the Sham and OE groups, but P4 treatment decreased the percentage of CD45^+^IL-10^+^TGF-β1^+^ Treg cells to a level comparable to that of the Sham and OE groups (Fig. 4C, G). These findings suggest that P4 treatment modulates the balance between Th1 and Th2, as well as Th17 and Treg cells, thus exhibiting its therapeutic effect.

### P4 treatment results in a decrease in the number of CD4^+^ T cells, but an increase in the Tregs in the lungs

**Figure 5:**
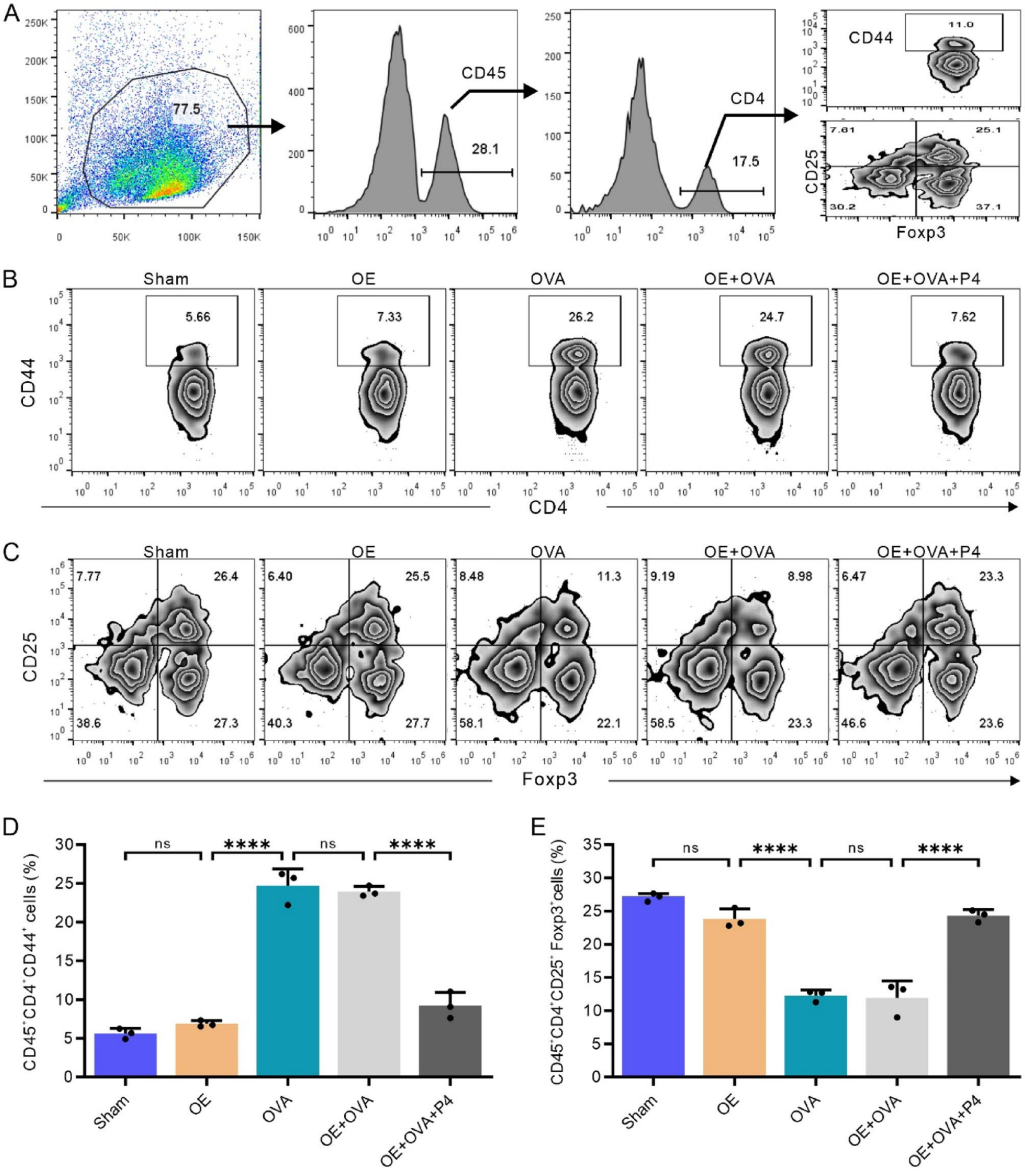
P4 treatment reduces the number of CD4^+^ T cells while increasing the number of Tregs in the lungs. **(A)** Flow cytometry gating strategy, showing that CD45^+^ immune cells were gated out from the total lung cell population for further analysis of CD4^+^CD44^+^ T cells and CD4^+^CD25^+^Foxp3^+^ Tregs. **(B,C)** Representative images depicting the analysis of CD45^+^CD4^+^CD44^+^ T cells (**B**) and CD45^+^CD4^+^CD25^+^Foxp3^+^ Tregs (**C**). **(D,E)** Percentages of CD45^+^CD4^+^CD44^+^ T cells (**D**) and CD45^+^CD4^+^CD25^+^Foxp3^+^ Tregs (**E**) in three samples. The experiments utilized two batches of mice, with data collected from the first batch. The number of experimental mice or samples is represented by the number of dots in each bar. Results are displayed as the mean ± SD. They were then statistically analyzed using one-way ANOVA followed by Tukey's post-hoc test; *P < 0.05, **P < 0.01, ***P < 0.001, and ****P < 0.0001.

Research has revealed that an imbalance in the Th1/Th2 ratio may be implicated in the development of allergic asthma, a condition characterized by an excessive Th2 immune response. To evaluate the efficacy of P4 therapy in suppressing the Th2 immune response in the airways of mice with OVA-induced allergies, we examined the levels of CD4^+^ T cells and Tregs in the lungs. Flow cytometry was utilized to analyze the percentages of CD4^+^CD44^+^ T cells and CD4^+^CD25^+^Foxp3^+^ regulatory T cells among the overall lung cells (Fig. 5A). The results showed that the OVA and OE + OVA groups had notably higher levels of CD4^+^CD44^+^ T cells compared to the Sham and OE groups (Fig. 5B, D). Conversely, the OVA and OE + OVA groups had significantly lower percentages of CD4^+^CD25^+^Foxp3^+^ regulatory T cells than the Sham and OE groups (Fig. 5C, E). Notably, P4 treatment in the OE + OVA + P4 group resulted in a significant reduction in the number of CD4^+^CD44^+^ T cells in the lungs (Fig. 5B, D) and a significant increase in the number of CD4^+^CD25^+^Foxp3^+^ regulatory T cells, comparable to the levels observed in the Sham and OE groups (Fig. 5C, E). These findings indicate that P4 therapy may be effective in reducing airway inflammation in mice with OVA-induced asthma by decreasing the number of CD4^+^ T cells and increasing the number of Tregs in the lungs.

### P4 treatment reduces neutrophil infiltration and NETosis, possibly by suppressing p38 signaling and decreasing the production of ROS in neutrophils

The results of the above experiments indicated no significant difference between the sham mice and OE mice. To conserve resources and minimize the use of mice, the following experiments on the correlation between progesterone and NETosis were conducted using only OE mice, which were divided into four groups: OE, OE + OVA, OE + OVP + P4, and OE + OVA + NEi. In addition, to determine whether P4 can reduce neutrophil infiltration and inhibit NETosis, we included a control group (OE + OVA + NEi) of mice treated with the neutrophil elastase inhibitor GW311616A (NEi) in the following experiments.

**Figure 6:**
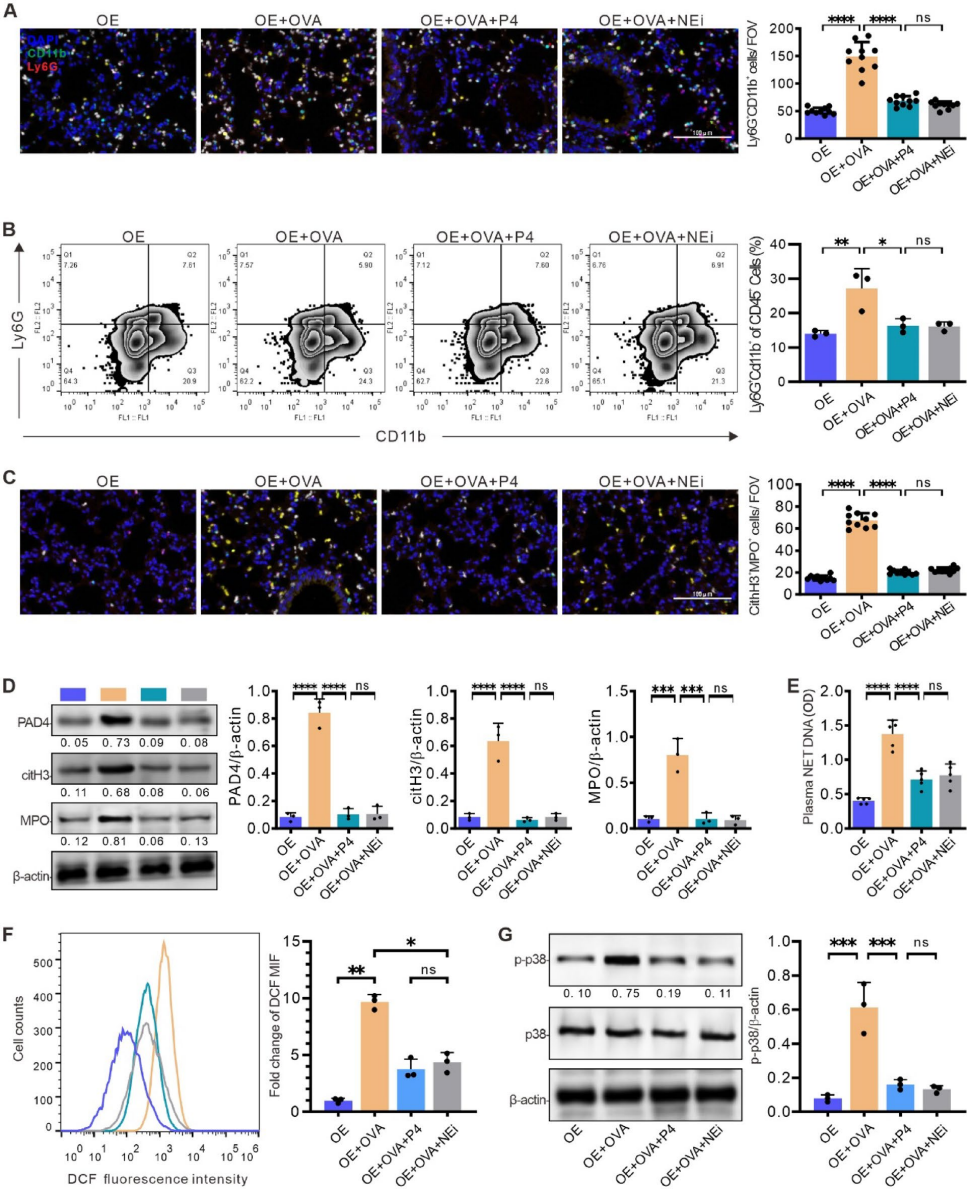
P4 treatment reduces neutrophil infiltration and NETosis, possibly by suppressing p38 signaling and decreasing the production of ROS in neutrophils. **(A)** Lung slides from mice, as indicated, were stained with neutrophil markers, anti-CD11b and anti-Ly6G antibodies, and DAPI. Representative images and quantification of colocalized cells in ten fields of view (FOV) are shown (n = 10). **(B)** Cytometry analysis of CD11b^+^ and Ly6G^+^ double-positive neutrophils in the lung tissues (n = 3). **(C)** Observation of MPO and citH3 colocalized NETs in the lung slides (FOV, n = 10). **(D)** Western blotting quantification of NET components PAD4, citH3, and MPO (n = 3). **(E)** Quantification of NET DNA in the serum (n = 3). (**F**) Flow cytometry analysis of ROS (DCF) levels in the neutrophils from the lung tissues treated with the indicated ingredients (n = 3). (**G**) Western blotting quantification of p38 and phosphorylated p38 (p-p38, n = 3). The experiments utilized two batches of mice, with data collected from the first batch. The number of experimental mice or samples is represented by the number of dots in each bar. Results are displayed as the mean ± SD. They were analyzed by one-way ANOVA with Tukey's multiple comparisons. *P < 0.05, **P < 0.01, ***P < 0.001, and ****P < 0.0001.

The efficacy of P4 in suppressing neutrophil infiltration into the lungs of mice with allergic airway inflammation was investigated using confocal microscopy and FCM analysis, with antibodies against two neutrophil biomarkers, CD11b and Ly6G. The results showed that the lungs from the OE + OVA group had a significantly higher number of double-positive neutrophils compared to the OE group; however, P4 or NEi treatment was able to reduce the number of neutrophils to a level similar to that of the OE group (Fig. 6A). FCM analysis yielded similar results to those observed with confocal microscopy, demonstrating that the percentage of CD11b^+^ and Ly6G^+^ double-positive neutrophils in the lungs of the OE + OVA group was significantly higher than in the OE group (Fig. 6B). These levels were significantly reduced by either P4 or NEi treatment (Fig. 6B). These findings indicate that P4 treatment could effectively impede neutrophil infiltration into the lungs of mice suffering from allergic airway inflammation.

A double staining technique involving FITC-conjugated antibodies against citH3 and PE-conjugated antibodies against MPO was used to investigate the effect of P4 on NETosis in the lungs of mice with allergic airway inflammation. The results showed an increase in citH3 and MPO double-positive cells in the OE + OVA group, which was reduced to a level similar to that of the OE group after P4 or NEi treatment (Fig. 6C). Western blot analysis also demonstrated that the levels of PAD4, citH3, and MPO expression in the lungs were higher than those in the OE group, but were significantly suppressed with P4 or NEi treatment (Fig. 6D). Additionally, ELISA analysis of serum NET-related dsDNA indicated that the levels of serum dsDNA were significantly higher in the OE + OVA group mice, but were reduced to levels comparable to those in the OE group after P4 or NEi treatment (Fig. 6E). Taken together, these findings suggest that P4 treatment can effectively inhibit NETosis in the lungs of RV-infected mice.

Recent research has demonstrated the crucial involvement of reactive oxygen species (ROS) in the process of NETosis, during which neutrophil elastase translocates from the cytoplasm to the nucleus in response to various stimuli^23,24^. Activation of p38 MAPK signaling has been identified as a trigger for ROS generation and subsequent NETosis^25,26^. Thus, we focused on evaluating the potential of P4 treatment to attenuate p38 signaling and diminish ROS levels in neutrophils. Flow cytometry analysis indicated a significant elevation in ROS levels in neutrophils from mice in the OE + OVA group compared to those in the OE group, with a subsequent reduction observed following treatment with P4 or Nei (Fig. 6F). Furthermore, the upregulation of phosphorylated p38 (p-p38) expression in neutrophils from the OE + OVA group confirmed the activation of p38 signaling, a response that was reversed by P4 or Nei intervention (Fig. 6G). These findings suggest that P4 treatment holds promise in inhibiting NETosis, possibly by suppressing p38 signaling and mitigating ROS production in neutrophils.

### P4 treatment induces comparable anti-inflammatory effects to those of a NETosis inhibitor

**Figure 7:**
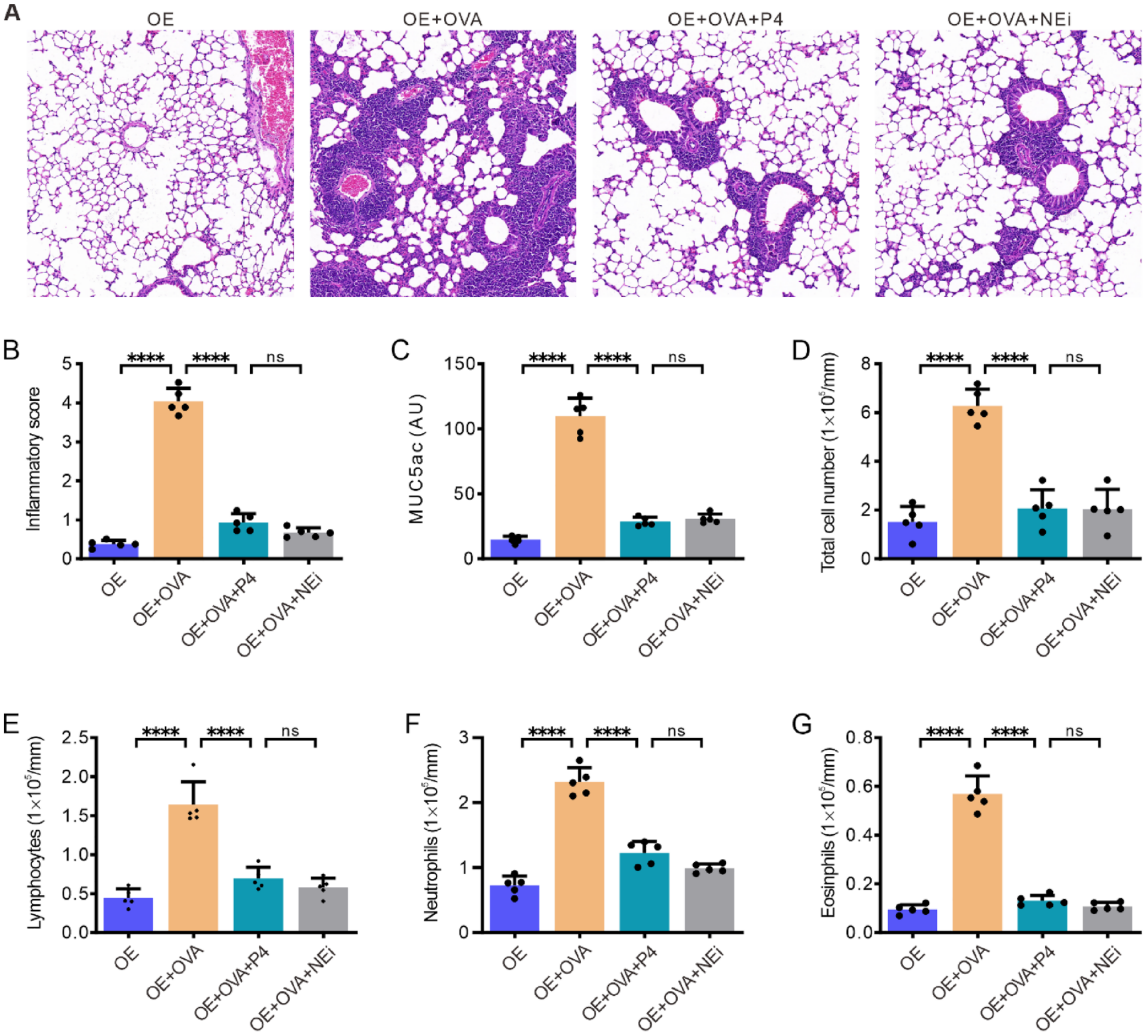
Progesterone treatment induces comparable anti-inflammatory effects to those of a NETosis inhibitor. **(A)** Characteristic images of the lung slides stained with H&E. **(B)** Inflammation scores determined based on the H&E stain. **(C)** Relative quantification of MUC5ac protein in the BALF. **(D–G)** Total inflammatory cell counts (**D**) and their subtypes, including lymphocytes (**E**), neutrophils (**F**), and eosinophils (**G**) in the BALF. The experiments utilized two batches of mice, with data collected from the second batch. The number of experimental mice or samples is represented by the number of dots in each bar. Results are displayed as the mean ± SD. They were then analyzed using one-way ANOVA followed by Tukey's post-hoc test. Statistical significance is represented as *P < 0.05, **P < 0.01, ***P < 0.001, and ****P < 0.0001.

Our further experiments were conducted to ascertain whether the anti-inflammatory effects of P4 were caused by its inhibition of NETosis. To this end, P4 or NEi was administered to OVA-induced inflammation mice, and the lung histology and inflammatory cells in the BALF were evaluated. The results showed that compared to the OE group, the OE + OVA group had increased peribronchial and perivascular infiltration of inflammatory cells, while treatment with either P4 or NEi significantly decreased the inflammatory cell infiltration (Fig. 7A) and the inflammatory score (Fig. 7B). Additionally, mucus production in the BALF of the OE + OVA group mice was higher than that of the other three groups but was significantly reduced with P4 or NEi treatment (Fig. 7C). Furthermore, the total number of inflammatory cells (Fig. 7D) and their subsets, including lymphocytes (Fig. 7E), neutrophils (Fig. 7F), and eosinophils (Fig. 7G), in the BALF of the OE + OVA group was significantly higher than those of the OE group. However, treatment with P4 or NEi resulted in a reduction of these parameters to levels comparable to those of the OE group (Fig. 7D–G). These findings suggest that the inhibition of NETosis by NEi alone has similar anti-inflammatory effects as P4, implying that P4 exerts its anti-inflammatory capacity through the inhibition of NETosis in the pulmonary environment.

### P4 treatment induces comparable effects on the modulation of the immune microenvironment as those of a NETosis inhibitor

**Figure 8:**
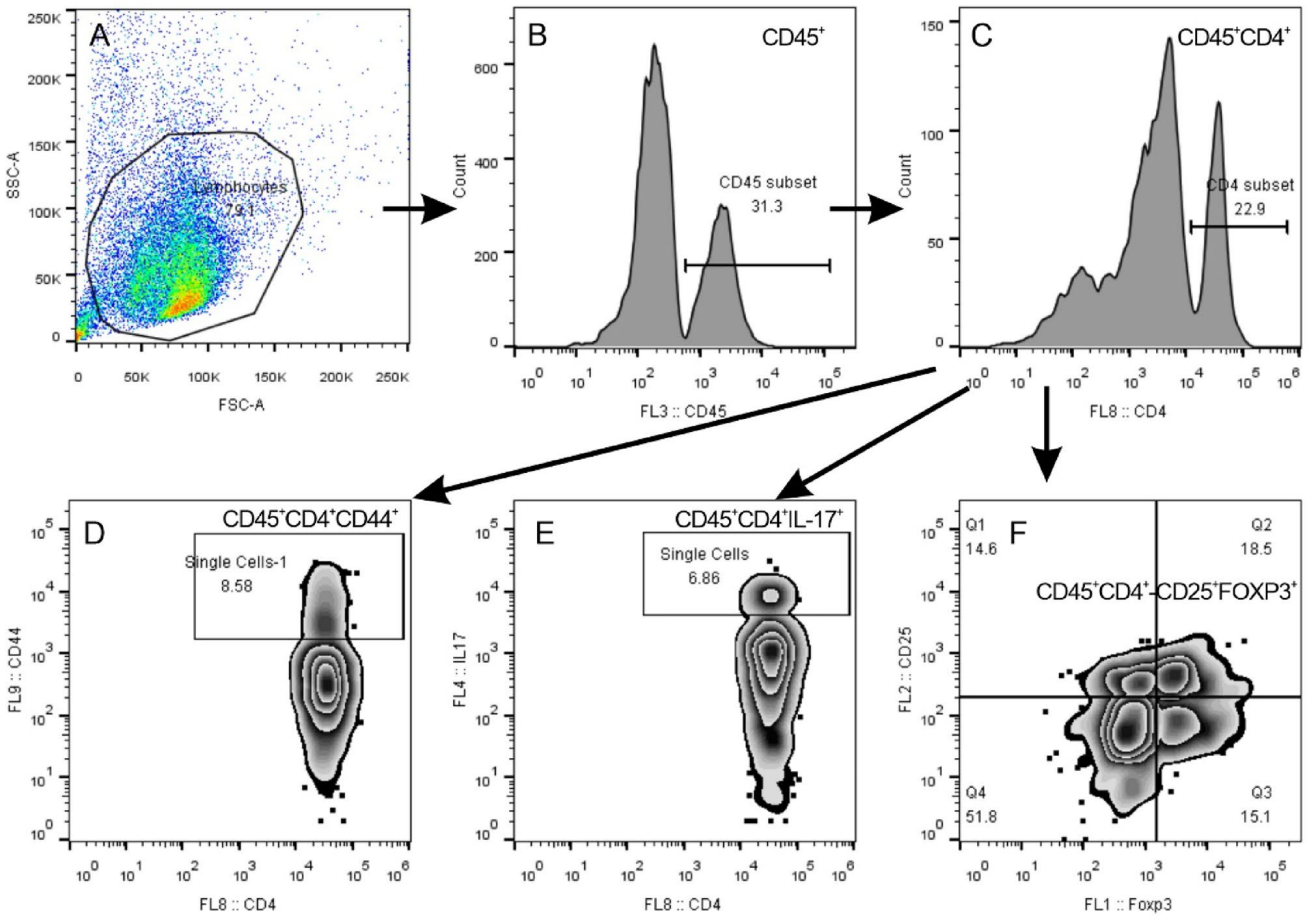
FCM gating strategy for gating out the CD4^+^ subset of immune cells for further analysis in Fig. 9. (**A**) FSC and SSC gates are used to differentiate intact cells from the single cells of lung tissue and to eliminate cell debris. (**B**) CD45^+^ immune cells are isolated from the intact cells in (**A**). **(C)** CD45^+^CD4^+^ T cells are separated from the CD45^+^ immune cells in (**B**). **(D–F)** The subset of CD45^+^CD4^+^ T cells is further used to quantify the CD45^+^CD4^+^CD44^+^ T cells (**D**), CD45^+^CD4^+^IL-17^+^ Th17 cells (**E**), and CD45^+^CD4^+^CD25^+^Foxp3^+^ Treg cells (**F**), respectively.

Flow cytometry was used to assess the subtypes of immune cells present in lung tissue. By analyzing single cells within the lung tissue, we were able to quantify the percentages of CD45^+^CD4^+^CD44^+^ T cells, CD45^+^CD4^+^IL-17^+^ Th17 cells, and CD45^+^CD4^+^CD25^+^Foxp3^+^ Tregs in CD45^+^CD4^+^ immune cells using a flow cytometry gating strategy (Fig. 8).

**Figure 9:**
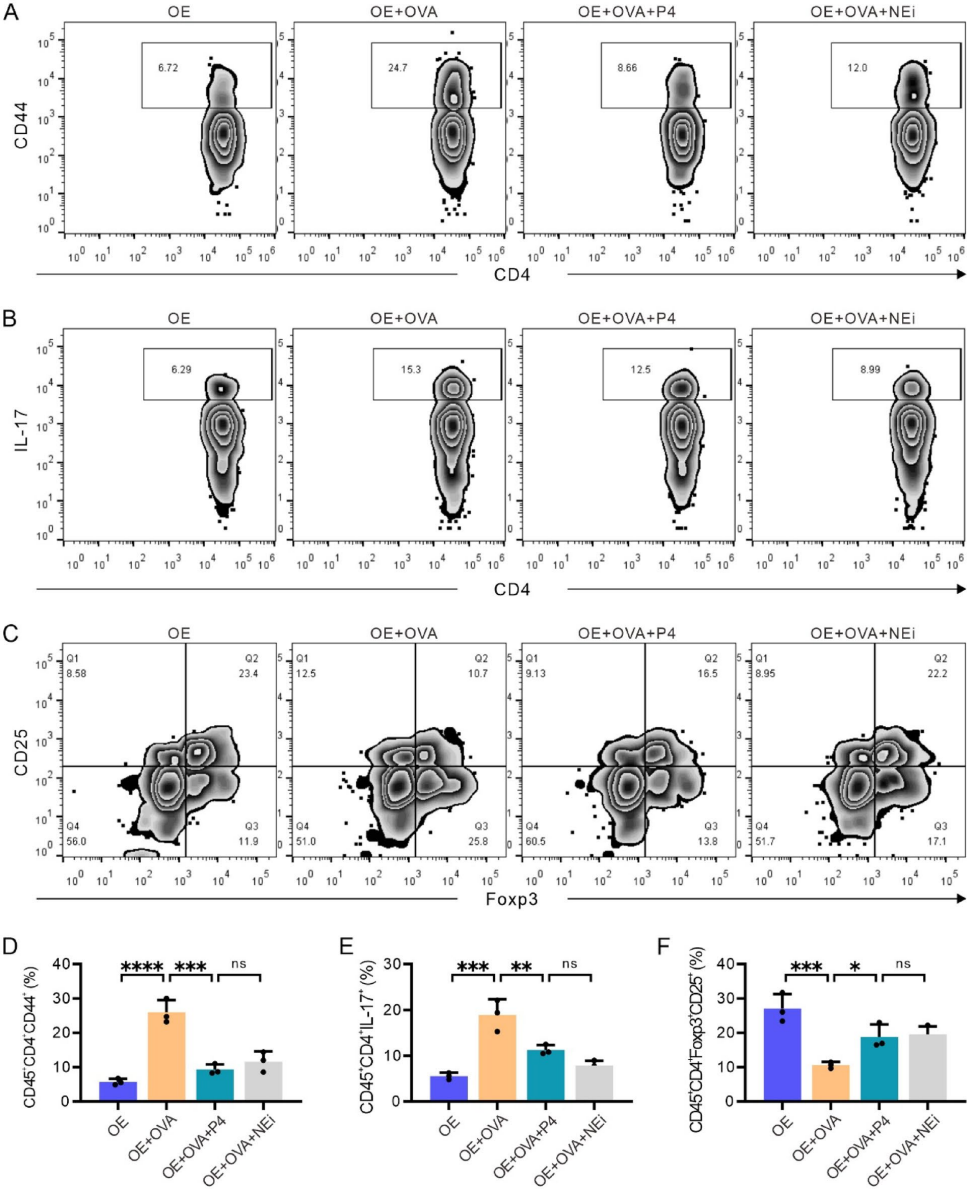
Progesterone treatment induces comparable effects on the modulation of the immune microenvironment as those of a NETosis inhibitor. The CD45^+^CD4^+^ subtypes indicated in this figure were gated out, as shown in Fig. 8. **(A–C)** Representative images depicting the analysis of CD45^+^CD4^+^CD44^+^ T cells (**A**), CD45^+^CD4^+^IL-17^+^ Th17 cells (**B**), and CD45^+^CD4^+^CD25^+^Foxp3^+^ Treg cells (**C**). **(D–F)** Quantitative data from FCM analysis, showing the percentages of CD45^+^CD4^+^CD44^+^ T cells (**D**), CD45^+^CD4^+^IL-17^+^ Th17 cells (**E**), and CD45^+^CD4^+^CD25^+^Foxp3^+^ Treg cells (**F**). The experiments utilized two batches of mice, with data collected from the second batch. The number of experimental mice or samples is represented by the number of dots in each bar. Results are displayed as the mean ± SD. They were then statistically analyzed using one-way ANOVA followed by Tukey's post-hoc test; *P < 0.05, **P < 0.01, ***P < 0.001, and ****P < 0.0001.

The results showed that compared to the OE group, the OE + OVA group had an increased number of CD45^+^CD4^+^CD44^+^ T cells, while P4 or NEi treatment significantly reduced the number of pulmonary CD45^+^CD4^+^CD44^+^ T cells (Fig. 9A, D). Additionally, the percentage of CD45^+^CD4^+^IL-17^+^ Th17 cells was higher in the OE + OVA group than in the OE group, and P4 or NEi treatment also decreased this percentage (Fig. 9B, E). Conversely, the percentage of CD45^+^CD4^+^CD25^+^Foxp3^+^ Tregs was significantly decreased in the OE + OVA group compared to the OE group, but P4 or NEi treatment significantly increased the number of pulmonary CD45^+^CD4^+^CD25^+^Foxp3^+^ Tregs to a level comparable to that of the OE group (Fig. 9C, F). These findings suggest that NEi treatment has similar effects on the modulation of immune microenvironments as P4 treatment, implying that P4 modulates immune microenvironments by inhibiting NETosis in the lungs of OVA-induced allergic inflammation.

### P4 treatment partially impacts the immune microenvironment by inhibiting the release of dsDNA

**Figure 10:**
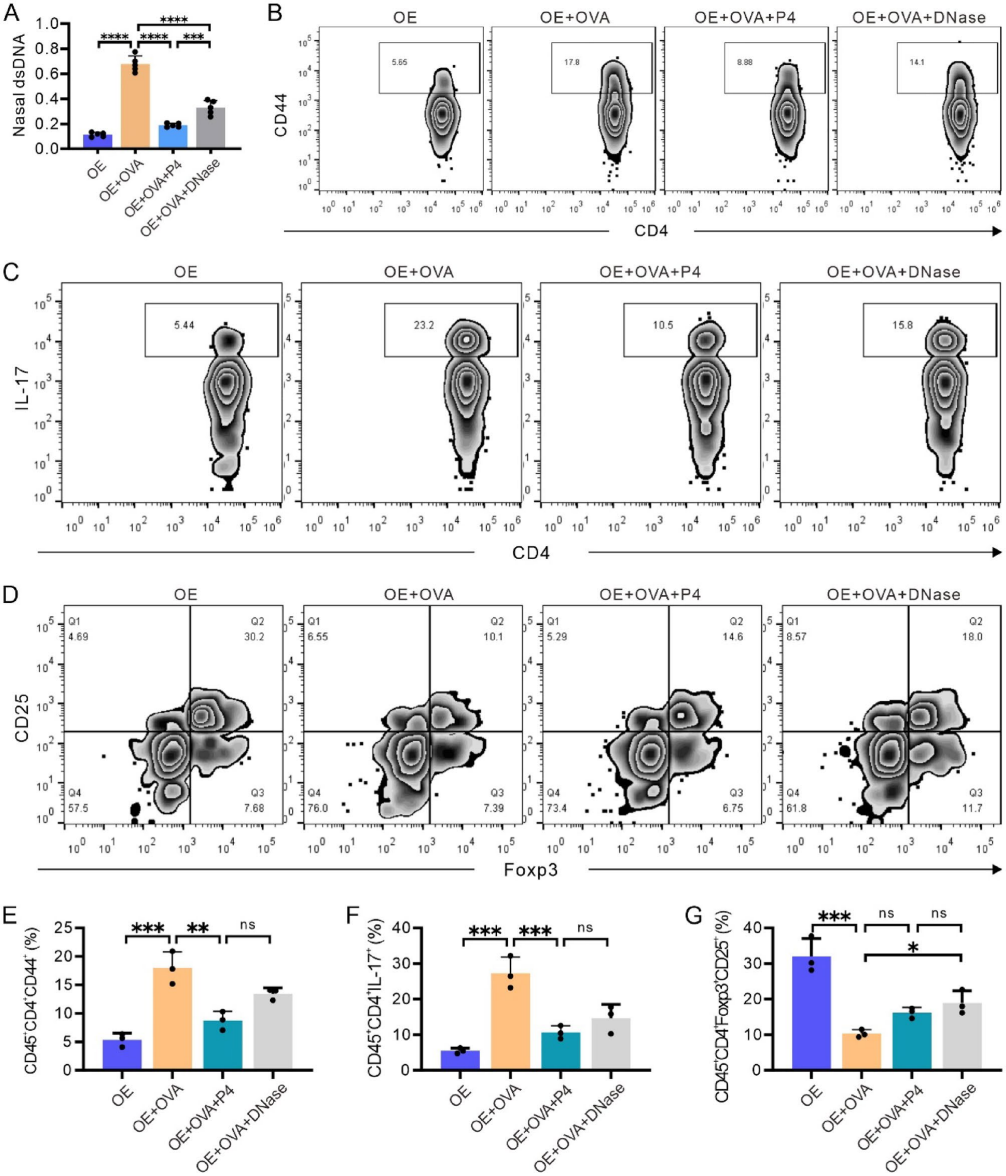
P4 treatment partially impacts the immune microenvironment by inhibiting the release of dsDNA. **(A)** Quantification of dsDNA in the BALF. **(B–D)** The CD45^+^CD4^+^ subtypes indicated were gated out, as shown in Fig. 8. Representative images depicting the analysis of CD45^+^CD4^+^CD44^+^ T cells (**B**), CD45^+^CD4^+^IL-17^+^ Th17 cells (**C**), and CD45^+^CD4^+^CD25^+^Foxp3^+^ Treg cells (**D**). **(E–G)** Quantitative data from FCM analysis, showing the percentages of CD45^+^CD4^+^CD44^+^ T cells (**E**), CD45^+^CD4^+^IL-17^+^ Th17 cells (**F**), and CD45^+^CD4^+^CD25^+^Foxp3^+^ Treg cells (**G**). The experiments utilized two batches of mice, with data collected from the second batch. The number of experimental mice or samples is represented by the number of dots in each bar. Results are displayed as the mean ± SD. They were then statistically analyzed using one-way ANOVA followed by Tukey's post-hoc test; *P < 0.05, **P < 0.01, ***P < 0.001, and ****P < 0.0001.

A recent study has shown that host dsDNA released during NETosis contributes to airway inflammation and exacerbates type-2 acute asthma triggered by rhinovirus infection^27^. Building upon this discovery, we examined the influence of dsDNA on the immune microenvironment. We conducted an experiment involving a cohort of OVA-induced asthma mice that were administered deoxyribonuclease I (DNase) to eliminate dsDNA from their airways. Subsequently, we measured dsDNA levels in the BALF. Our findings revealed a significant elevation in dsDNA levels in the BALF of mice in the OVA group compared to the OE group. Treatment with P4 or DNase resulted in a notable decrease in dsDNA levels (Fig. 10A). However, the reduction observed with DNase treatment was less pronounced than that with P4 treatment, and a statistically significant difference was observed between the two treatments (p < 0.001, Fig. 10A).

Flow cytometry was utilized to examine the impact of DNase treatment on the immune microenvironment in lung tissue. The subtypes of immune cells were assessed using a consistent gating strategy as depicted in Fig. 8. Our findings revealed that the OE + OVA group exhibited a higher count of CD45^+^CD4^+^CD44^+^ T cells compared to the OE group. Conversely, treatment with P4 or DNase led to a notable reduction in pulmonary CD45^+^CD4^+^CD44^+^ T cells (Fig. 10B, E). Moreover, the percentage of CD45^+^CD4^+^IL-17^+^ Th17 cells was elevated in the OE + OVA group compared to the OE group, while treatment with P4 or DNase resulted in a decrease in this percentage (Fig. 10C, F). On the other hand, there was a significant decrease in the percentage of CD45^+^CD4^+^CD25^+^Foxp3^+^ Tregs in the OE + OVA group compared to the OE group, but treatment with P4 or DNase partially increased the count of pulmonary CD45^+^CD4^+^CD25^+^Foxp3^+^ Tregs (Fig. 10D, G). Furthermore, it was noted that although DNase treatment improved these indicators in the lungs, the overall effect was inferior to that of P4 treatment (Fig. 10E-G). These results suggest that P4 treatment partially modulates the immune microenvironment by suppressing the release of dsDNA.

## Discussion

Recent studies have indicated that P4 may have potential benefits in the management of acute or chronic inflammatory conditions, such as COVID-19^19,20^, Neisseria gonorrhoeae^28^, influenza^29^, and stress-induced inflammation^30^. Additionally, P4 produced by pregnant women may reduce estrogen, chorionic gonadotropin, and granulocyte colony-stimulating factor-induced NETosis during normal pregnancy ^31^. To explore the effects of P4 on allergic airway inflammation, a mouse model of OVA-induced allergic asthma was employed in our current study. The results showed that P4 treatment effectively ameliorated asthma symptoms, such as sneezing, nasal itching, difficulty breathing, and cyanosis. Furthermore, P4 treatment was associated with reduced inflammation scores and areas stained by Periodic Acid-Schiff (PAS) in the lungs of treated mice. Flow cytometry and histology analyses revealed a significant decrease in CD45-positive inflammatory cells, including eosinophils, lymphocytes, neutrophils, and macrophages, in the lung tissues and BALF of the P4-treated mice in the OE + OVA + P4 group. Our research indicates that P4 treatment is effective in reducing airway inflammation in a mouse model of OVA-induced allergic asthma. Asthma is a chronic condition characterized by persistent inflammation of the airways, caused by the activation of various cells, including eosinophils, lymphocytes, neutrophils, macrophages, and epithelial cells^32^, which release proinflammatory cytokines, resulting in airway hyperresponsiveness and long-term symptoms. Allergen-induced airway inflammation is associated with an increase in IgE levels in the blood plasma and infiltration of eosinophils into the airways^33^. Evidence from studies and our own research suggest that P4 may be a promising anti-inflammatory option for treating asthma. Therefore, early intervention with anti-inflammatory agents such as P4, which can reduce inflammatory changes, may be beneficial in reversing airway obstruction and halting the progression of airway remodeling.

Although NETosis is a vital immune response, too much or dysfunctional NETosis can lead to inflammatory responses, tissue damage, and a variety of diseases, including sepsis, systemic lupus erythematosus, rheumatoid arthritis, small-vessel vasculitis, inflammatory bowel disease, COVID-19, and cancer metastasis^34–37^. In our current study, we found that NETosis is associated with modulation of the immune microenvironment in mice with OVA-induced allergic airway inflammation. This was shown by the different changes in Th1/Th2, Th17/Treg and Th2 CD4^+^CD44^+^ T cells after treatment with GW311616A (NEi), a neutrophil elastase inhibitor, which inhibited the production of NETosis and had comparable effects to those of P4 treatment. This is believed to be the first report of NETosis being able to regulate the immune microenvironment that exacerbates allergic airway inflammation. Consequently, the development of effective therapies to inhibit the process and/or products of NETosis could have a wide range of applications in treating not only allergic airway inflammation, but also other inflammatory diseases that are mediated by NETosis.

Evidence is mounting that non-eosinophilic airway inflammation, particularly neutrophilic inflammation, may be involved in the development of fatal, severe, and steroid-resistant asthma^38^. In this study, the infiltration of neutrophils and the generation of NETosis in the OVA-induced allergic airway inflammation mouse model was investigated. It was found that CD11b^+^Ly-6G^+^ neutrophils were increased in the OVA-group mice, whereas treatment with P4 significantly reduced their infiltration into lung tissues. Flow cytometry analysis revealed a marked increase in the percentage of CD11b^+^Ly-6G^+^ neutrophils in the OVA group, but a significant decrease in the P4 group. Interestingly, NEi treatment alone had comparable effects in terms of inhibiting airway inflammation and modulating the immune microenvironment as the P4 treatment did. These results suggest that P4 may be effective in preventing not only allergic inflammation, but also other inflammatory diseases that are mediated by NETosis. Further research is required to explore the therapeutic effects of P4 on NETosis-mediated inflammatory diseases.

In individuals with asthma, there is an imbalance between two types of T cells known as Th1 and Th2 cells within the pulmonary microenvironment^16^. Th2 cells are mainly responsible for the allergic responses and asthma, as they secrete cytokines such as IL-4, IL-5, and IL-13. These cytokines can lead to the recruitment of eosinophils, inflammation of the airways, and the production of IgE antibodies^39,40^. Conversely, Th1 cells are involved in cellular immunity and secrete IFN-γ and IL-2, which possess anti-inflammatory properties and can suppress the activity of Th2 cells^41,42^. This imbalance, with an excessive presence of Th2 cells and a decreased population of Th1 cells in asthmatic lungs, results in an overreactive Th2-associated inflammatory response, causing persistent airway inflammation, bronchial hyperresponsiveness, and the typical symptoms of asthma^16^. Our recent study has demonstrated that P4 treatment has a positive effect on the number of Th1 cells and a negative effect on the number of Th2 cells in the lungs of mice with OVA-induced asthma. This suggests that P4 may be a potential anti-inflammatory agent for the treatment of asthma. Nevertheless, further research is needed to fully comprehend the regulatory pathways between Th1 and Th2 cells in response to P4 treatment, as well as their contribution to the development and severity of asthma.

T cells play a crucial role in the immune system and have been found to have two subgroups, Th17 and Treg cells, in individuals with asthma. Th17 cells are responsible for releasing pro-inflammatory cytokines, such as IL-17, which regulate inflammatory responses, while Treg cells are immunosuppressive and help maintain immune balance^43^. It has been suggested that an imbalance between Th17 and Treg cells is associated with the development and progression of asthma symptoms^44,45^. Studies have shown that individuals with asthma have an increased number and activity of Th17 cells, while the number and functionality of Treg cells are decreased ^46^. This imbalance can lead to an exaggerated inflammatory response and heightened airway hyperresponsiveness^47^. In order to further investigate this phenomenon, our study focused on the impact of P4 treatment on the balance between Th17 and Treg cells in mice with OVA-induced allergic asthma. The results of our study revealed that P4 treatment had a positive effect on the number of Treg cells, while negatively affecting the number of Th17 cells in the lungs of mice with OVA-induced allergic asthma. These findings suggest that P4 could serve as a potential anti-inflammatory agent by modulating the Th17/Treg balance in asthma. However, it is important to note that our present study is limited to evidence from a single animal experiment. We also lack a comprehensive in vitro and clinical experimentation. Therefore, the precise mechanisms underlying the interplay between Th17 and Treg cells in asthma remain unclear. Further research is needed to gain a better understanding of their roles in asthma and to explore potential therapeutic approaches based on these findings.

Recent studies have shed light on the role of the immune microenvironment in the progression of allergic asthma, both in humans and animals^48^. In particular, two distinct types of allergen-specific Th2 memory cells have been identified: central memory T cells (TCM) and effector memory T cells (TEM)^49,50^. TCM cells are capable of self-renewal and establishing long-term memory, while TEM cells have slower rates of proliferation and behave similarly to effector T cells^51^. In our current study, we evaluated the impact of P4 treatments on pulmonary memory CD4^+^ T cells using an OVA-induced chronic allergic mouse model. The results indicated that both treatments significantly reduced the number of CD4^+^CD44^+^ T cells, most of which were memory T cells, in addition to reducing lung inflammation and lower levels of Th2 cytokines, such as IL-4, IL-5, and IL-13. These findings suggest that P4 may suppress the CD4^+^ T cell response, thus mitigating Th2 inflammation. In light of previous research on Th1/Th2 and Th17/Treg, these findings provide further evidence that P4 treatment may be effective in alleviating allergic airway inflammation by modulating the immune microenvironment in OVA-induced allergic asthma in mice. As such, further research is needed to gain insight into the mechanisms by which P4 affects the modulation of the lung microenvironment in allergic asthma.

Asthma is a common long-term inflammatory condition that affects the airways and is characterized by underlying inflammatory processes. There are two main types of asthma: eosinophilic asthma and neutrophilic asthma^52,53^. Eosinophilic asthma, which is present in approximately half of asthma patients, is identified by increased levels of eosinophils and type 2 allergic inflammation in the airways^54^. This involves communication between T2 helper cells and innate lymphoid cells. Neutrophilic asthma, on the other hand, is characterized by non-T2 inflammation in the airways, involving Th1 and Th17 cells, as well as cytokines such as IL-6, IL-8, and TNF-α^54^. Recent researches demonstrate that both neutrophils and eosinophils are capable of forming and releasing extracellular traps (ETs)^55^, among which release nuclear dsDNA into an extracellular structure and embedding granule proteins in this structure. Research has indicated that excessive levels of ETs can damage the airway epithelium and trigger an inflammatory response, exacerbating asthma. In this study, it was observed that there was a notable presence of dsDNA in the serum and BALF of mice with OVA-induced asthma. Treatment with P4 effectively reduced the release of dsDNA, and using RNase to eliminate dsDNA from the lungs yielded similar results to P4 treatment in enhancing the immune environment of the lungs. This suggests that dsDNA may influence the immune environment of the lungs. The OVA-induced asthma model utilized in this study is a commonly used experimental model for studying asthma development. In this model, mice are sensitized to OVA and then exposed to OVA to induce an inflammatory response. Neurological inflammation typically precedes eosinophilic inflammation in this model and is present throughout the progression of asthma pathology. Our study observed an increase in the number of neutrophils and eosinophils in the BALF of OVA-induced asthma model mice. However, our methods were unable to determine the origin of the dsDNA in the BALF, whether it stemmed from neutrophils or eosinophils. Therefore, further research is necessary to identify the specific source of dsDNA in the lungs and its role in asthma development and the modulation of the asthma immune microenvironment in the future.

In this study, we investigated the inhibitory effects of P4 on inflammation associated with allergic asthma and its relationship with the immune milieu of asthma in a murine model. Our findings suggest that the therapeutic advantages of P4 are closely associated with the inhibition of NETosis, potentially mediated by the reduction of reactive oxygen species (ROS) production through the blockade of the p38 signaling pathway. It is important to note that the results presented in our research primarily consist of observational findings. Due to various limitations, we did not extensively explore the molecular mechanisms underlying the ability of P4 to suppress NETosis, thus underscoring a significant research gap. At present, P4 exhibits specific binding capabilities to two types of progesterone receptors: progesterone receptors (PRs) and membrane progesterone receptors (mPRs). The latter, characterized as high-affinity receptors, are membrane-associated and facilitate rapid activation of distinct intracellular signaling pathways, diverging from the classical pathways governed by PRs^48^. These non-classical pathways are affiliated with the progestin and adipoQ receptor (PAQR) family, which is distinct from the G protein-coupled receptor (GPCR) superfamily. Phylogenetic analysis has identified five vertebrate mPR subtypes: mPRα (PAQR7), mPRβ (PAQR8), mPRγ (PAQR5), mPRδ (PAQR6), and mPRε (PAQR9)^48^. These mPRs are prevalent in various mammalian tissues, including reproductive, non-reproductive, and cancerous tissues^48, 61^. Recent research has revealed the expression of mPRs in the human immune system, notably in monocytes, macrophages, dendritic cells, and lymphocytes^62, 63^. Hence, we postulate that P3 could potentially impede the p38 signaling pathway, thereby suppressing RSO generation either directly or indirectly through these receptors. Nevertheless, additional systematic experiments are imperative to ascertain whether this inhibition occurs through these receptors or alternative pathways. Through the publication of this research, our objective is to capture the interest of our peers in the field, and we are open to engaging in a comprehensive exploration of the molecular mechanisms by which P4 inhibits NETosis.

In summary, the findings of the study suggest that the administration of P4 was successful in mitigating OVA-induced allergic airway inflammation, as demonstrated by a decrease in the influx of inflammatory cells in both the lungs and BALF of the mice. Additionally, P4 therapy was observed to diminish the production of airway mucus. Moreover, P4 had a notable effect on the immune microenvironment within the lungs, fostering a balance between Th1/Th2 and Treg/Th17 cells, CD4^+^ T cells, and controlling NETosis initiated by OVA-induced allergies. These results point to P4 as a potential treatment for allergic inflammation, and manipulating the immune microenvironment by maintaining equilibrium between Th1/Th2 and Treg/Th17 cells may be an effective approach for managing such conditions.

## Materials and methods

### Ethics approval for animal experiments

All research studies involving animals were conducted in accordance with the ARRIVE guidelines and have been granted ethical approval from Hainan Medical University (approval number: ETH_HMU202000226). This ensures that the study adheres to national and international guidelines for the care and use of laboratory animals.

### Mice and their groups

A total of fifty female BALB/c mice, aged between 5 and 6 weeks and weighing 20–25 g, were obtained from the Chengdu Laboratory Animal Center in China for this study. The mice were carefully chosen to ensure uniformity in age and weight. To provide a suitable environment for the mice, they were kept in a controlled facility with a temperature ranging from 20 to 24 °C, humidity maintained at 55 ± 5%, and subjected to a 12-h light and dark cycle. The mice had unrestricted access to food and water throughout the experiment. The experimental protocols and conditions were approved by the Ethics Review Board for Animal Studies of Hainan Medical University and conducted in accordance with the guidelines set by the National Institutes of Health and the Animal Care and Use Ethics Committee of Hainan Medical University, China. Strict ethical standards were followed throughout the study. To induce airway allergic inflammation and create an allergic asthma model^56^, the fifty mice were randomly divided into five groups. The first group, referred to as the Sham group, consisted of unovariectomized mice that were sensitized and exposed to normal saline. The second group, known as the OVA group, included unovariectomized mice that were sensitized and exposed to OVA. The third group, designated as the OE group, consisted of ovariectomized mice that were sensitized and exposed to normal saline. The fourth group, referred to as the OE + OVA group, consisted of ovariectomized mice that were sensitized and exposed to OVA. The fifth group, known as the OE + OVA + P4 group, consisted of mice that had undergone ovariectomy, were sensitized and exposed to OVA, and received P4 treatment. The dosage of P4 given was determined based on a previous study and our own preliminary experiments. A detailed experimental timeline and protocols followed in this study are shown in Fig. 1A. Various experiments were conducted on appropriate specimens, such as BALF, including histological staining to determine the number of different types of inflammatory cells, ELISA to measure cytokine levels, and flow cytometry to analyze inflammatory cell percentages. To ensure an adequate amount of BALF for the experiments, five specimens were used for ELISA tests and three for flow cytometry analysis. The remaining two specimens, along with specimens from other mice, were examined for inflammatory cells through staining. This process was also applied to lung tissue specimens. Results were reported based on specimens from three to five experimental mice randomly selected per group. Two batches of mice were used for testing, with results in Figs. 1, 2, 3, 4, 5 and 6 from the first batch and Figs. 7, 8, 9 and 10 from the second batch.

### Ovariectomy

In this study, female mice were administered an intraperitoneal injection of 8 mL/kg of 1% pentobarbital sodium for anesthesia. After seven days, the ovaries were extracted from both sides of their backs, in accordance with a procedure similar to one reported previously^56^. To guarantee the mice's welfare, a thermostatic blanket was used to keep their body temperatures while they regained consciousness. For the control group (Sham), the same steps were taken, except for the exclusion of ovary removal.

### Establishment of the OVA-mediated allergic asthma mouse model and treatment of mice

In accordance with a prior study^57^, an experimental protocol was conducted to induce allergic asthma in mice. This procedure involved performing ovariectomy, followed by sensitizing the mice to OVA through intraperitoneal injection of a mixture containing 20 μg OVA and 2 mg potassium aluminum sulfate dissolved in 0.5 mL of normal saline, repeated on Day 0 and Day 7. Subsequently, from Day 14 to Day 32, the mice were exposed to aerosolized 1% OVA for a duration of 30 min each day via an ultrasonic nebulizer, in a regimen comprising five consecutive days of OVA inhalation followed by two days of rest, with a total of three cycles completed (as shown in Fig. 1A). Two control groups, namely the Sham group and the OE group, were administered an equal volume of normal saline instead of undergoing OVA sensitization or inhalation challenge. Conversely, the treatment group, designated as OE + OVA + P4, received subcutaneous injections of P4 at a dosage of 30 μg/kg/day every two days commencing from Day 14 until Day 35. The P4 was initially dissolved in sesame oil to achieve a concentration of 5 μg/mL prior to administration. The OE + OVA group, on the other hand, was given the same volume of sesame oil as a control treatment. After the experimental protocol was completed, the mice were euthanized on Day 36 by administering an intraperitoneal injection of a combination of Ketamine (250 mg/kg) and Xylazine (25 mg/kg) until the toe pinch and corneal reflex were no longer present, as previously reported^58^. Blood samples were procured for serum analysis, and BALF from the left lung was obtained. The right lung was used for histopathological examination, immunofluorescence staining, real-time reverse transcription polymerase chain reaction (qPCR), Western blot analysis, and isolation of single cells for flow cytometry analysis. An illustration of the entire experimental protocol can be seen in Fig. 1A.

In line with the administration of P4, the neutrophil elastase inhibitor GW311616A (NEi, 50 μg, AxonMedChem, Groningen, Netherlands) and DNase I (500 IU, Sigma-Aldrich) were also administered through inhalation following the same protocol as P4, as illustrated in Fig. 1A, as detailed in a previous study^27^.

### Categorization of the inflammatory cells in the BALF

The collection of BALF from the left lungs of mice was conducted using a previously reported method^57^. This method involved the introduction and removal of 0.5 mL of phosphate-buffered saline (PBS) three times via a tracheal cannula. The collected BALF was then centrifuged at a low speed of 800×*g* at a temperature of 4 °C for a duration of five minutes. The cell debris was re-suspended using 200 μL of PBS and applied to slides by cytospinning. To stain the slides, Wright Stain solution (Boster Biology, Wuhan, China) was used. To determine the total count of inflammatory cells and their different subtypes (lymphocytes, monocytes, neutrophils, and eosinophils), a minimum of 200 cells were counted under a hemocytometer. The cell-free supernatants were stored at −80 °C for subsequent detection of related cytokines.

### Lung histology

The right lung tissue was fixed for 24 h in 4% paraformaldehyde to ensure preservation, after which it was embedded in paraffin and cut into 5 μm sections. Hematoxylin and eosin staining (H&E) was then conducted according to a standard protocol in order to determine the degree of airway inflammation, which was evaluated using a validated scoring system^59^. Additionally, Periodic Acid-Schiff staining (PAS) was employed to quantify the number of goblet cells containing glycoproteins in the bronchial walls, providing an estimate of goblet cell proliferation and mucus production. The goblet cell proliferation score, which illustrates the extent of goblet cell proliferation, was then calculated based on a methodology described in a previous study^59^.

### Flow cytometry

FCM was utilized in this study to evaluate the presence of inflammatory cells in BALF and to characterize the CD45 positive Th1, Th2, Th17, and Tregs cells in lung tissue, following previously established protocols^60–63^. The procedure involved incubating single cells from BALF and digested lungs with specific conjugated antibodies obtained from Abcam (Cambridge, MA). These antibodies included APC-anti-CD45 (ab210182), PerCP-anti-CD3 (ab157317), FITC-anti-CD4 (ab269349), FITC-anti-Ly6G (ab25024), PE-anti-Siglec-F (ab240998), PE-anti-CD25 (ab25175), PE/Cy5-anti-Foxp3 (ab272247), and PerCP/Cy5.5-anti-CD44 (ab234445). The incubation was carried out at a temperature of 4 °C for a duration of 20–30 min. Subsequently, the CD45 positive cells were identified and sorted from the total cells in the lungs using the CD45^+^ channel. The same methodology was employed to analyze the inflammatory cells in BALF. The stained cells were then evaluated using the FACSAria II FCM system manufactured by BD Bioscience (Lake Franklin, NJ, USA). The resulting FCS files were further analyzed and the images of the results were obtained using FlowJo software version 10 (https://www.flowjo.com/solutions/flowjo) provided by BD Biosciences.

In order to measure levels of reactive oxygen species (ROS), a single-cell suspension from the lung was prepared and stained with antibodies targeting CD45, CD11b, and Ly6G. Additionally, the H2DCFDA-Cellular ROS Assay Kit (ab113851, Abcam) was used according to the manufacturer's instructions. Neutrophils (CD45^+^CD11b^+^Ly6G^+^) were gated out, and the levels of ROS (DCF) in the neutrophils were further quantified using the FACSAria II flow cytometry system. Subsequently, the FCS files were analyzed with FlowJo software version 10, as mentioned above.

### Enzyme-linked immunosorbent assay

To measure the levels of inflammatory cytokines in BALF, we conducted an enzyme-linked immunosorbent assay (ELISA) using a commercial kit from Boster Bioscience (Wuhan, China). This kit allowed us to assess the concentrations of various cytokines, including IFN-γ, TNF-β, IL-4, IL-5, IL-13, IL-10, TGF-β1 and IL-17. The detection of the reaction was carried out at 450 nm using an enzyme marker and a 96-well plate reader. In order to calculate the concentrations of these cytokines, we established their respective standard curves, as previously described in a published study^64^. We followed the instructions provided by the manufacturer to ensure accurate and reliable measurements.

### Detection of dsDNA in vitro

The QuantiT PicoGreen dsDNA Assay Kit (Thermo Fisher Scientific) was used to determine the levels of NET-related dsDNA present in the BALF or serum, following the manufacturer's instructions. The data were obtained using the ELX808IU spectrofluorometer reader (Bio-Tek, VT), and the concentration of dsDNA was calculated.

### Fluorescence microscopy

Immunostaining was performed on lung slides (3–5 μm) for two hours at 37 °C using specific antibodies, including anti-citrullinated histone H3 (citH3, 1:100; ab5103 Abcam, Cambridge, UK), anti-myeloperoxidase (1:40; af3667, R&D Systems), anti-CD11b (ab25175, Abcam), and anti-Ly6G (ab25024, Abcam). The slides were then washed with phosphate-buffered saline (PBS) and secondary antibodies conjugated with Alexa Fluor 568 (1:200, polyclonal, Thermo Fisher) or Alexa Fluor 488 (1:200, polyclonal, Thermo Fisher) were applied to the primary antibodies that did not have fluorescent labels. Additionally, the cell nuclei were stained with DAPI (1:1000; 10236276001, Roche) for 15 min at room temperature. The number of positive cells and the intensity of fluorescence were then evaluated under high-power fields, as previously described^65^.

### Western blotting analysis

Western blotting (WB) analysis was conducted to identify biomarkers associated with NETosis, such as PAD4, citrullinated histone H3 (citH3), and myeloperoxidase (MPO), as previously reported in the literature^60^. First, lung tissue samples were washed twice with phosphate-buffered saline (PBS) and then subjected to protein extraction using a lysis buffer. The protein content was measured using a bicinchoninic acid (BCA) assay kit (Thermo Scientific, USA). Subsequently, 30 µg of denatured total protein were separated by 10% sodium dodecyl sulfate–polyacrylamide gel electrophoresis (SDS-PAGE) and transferred onto a 0.45 μm polyvinylidene fluoride (PVDF) membrane. The membrane was then blocked with 5% skim milk at room temperature for one hour, and antibodies specific to PAD4 (1:80, ab214810, Abcam), citH3 (1:100; ab1791, Abcam), MPO (1:40; ab253440, Abcam), p38 (1:60, ab316937, Abcam), p-p38 (phosphor T180, 1:80, ab178867, Abcam), or β-actin (1:1000, Bioss, China) were added. After overnight incubation at 4 °C, a horseradish peroxidase (HRP)-conjugated secondary antibody (1:5000, Boster, China) was added and allowed to incubate for one hour at room temperature with shaking. Afterwards, the membrane was washed five times in tris-buffered saline-Tween 20 (TBST) solution for 10 min, and the protein content of the PVDF membrane was analyzed using ImageJ gel analysis software (https://imagej.net/software/nih-image).

### Statistical analysis

The mean ± SD was used to represent the resultant data. To analyze the differences among the various groups, a one-way ANOVA with Tukey correction for multiple comparisons was performed using GraphPad Prism (version 9.4.1). The significance level was determined by adjusted *P*-values, with a threshold of less than 0.05 considered statistically significant. The levels of significance were denoted as follows: * (P < 0.05), ** (P < 0.01), *** (P < 0.001), and **** (P < 0.0001).

### Supplementary Information

Supplementary Information.

## Data Availability

All data created or analyzed during this study are enrolled in this published article or are available from the corresponding author on reasonable request.

## Abbreviations

BALF: Bronchoalveolar lavage fluid
DNase: Deoxyribonuclease I
ELISA: Enzyme-linked immunosorbent assay
FCM: Flow cytometry
H&E: Haematoxylin and eosin
Ly-6G: Lymphocyte antigen 6G
mPRs: Membrane progesterone receptors
NS: Normal saline
OE: Ovariectomy
OVA: Ovalbumin
P4: Progesterone
PAS: Periodic acid–Schiff
PBS: Phosphate-buffered saline
PRs: Nuclear progesterone receptors
ROS: Reactive oxygen species
TCM: Central memory T cell
TEM: Effector memory T cell
Treg: Regulatory T cell
WB: Western blotting
