# Supplementary material for: Progesterone modulates the immune microenvironment to suppress ovalbumin-induced airway inflammation by inhibiting NETosis
Source: Sci Rep. 2024 Jul 26;14:17241. doi: 10.1038/s41598-024-66439-6 (PMC11282239; doi:10.1038/s41598-024-66439-6)

WB and Flowcytometry Original Data

Progesterone modulates the immune microenvironment to suppress ovalbumin-induced airway inflammation by inhibiting NETosis

Lin Wang1#, Feng-Ying Huang1, 2*, Shu-Zhen Dai1#, Yongshu Fu1, Xiangdong Zhou1, Cai-Chun Wang1*, Guang-Hong Tan1, 2*, Qi Li1*

1. Department of Respiratory Medicine, The First Affiliated Hospital of Hainan Medical University & Hainan Province Clinical Medical Center of Respiratory Disease, Haikou 570102, China

2. Key Laborato1y of Tropical Translational Medicine of Ministry of Education & School of Tropical Medicine, Hainan Medical University, Haikou 571199, China.

# These authors contributed equally to the work.

*Corresponding authors: Prof. Qi Li or Cai-Chun Wang or Department of Respiratory Medicine, The First Affiliated Hospital of Hainan Medical University, Haikou 570102, China. Tel: (+86) 139-7611-8659, Fax:(+86) 898-6652-8130, E-mail: yctanyc@126.com (WCC) or lqlq198210@sina.com (LQ); Or Prof. Guang-Hong Tan, E-mail: tanhoho@163.com, Key Laboratory of Tropical Translational Medicine of Ministry of Education & School of Tropical Medicine, Hainan Medical University, Haikou 571199, China.

Three replicates of WB data for Figure 6D……...1-2

Three replicates of WB data for Figure 6F……..3-4

Flowcytometry for Fig. 2F…………………………..……………………….5-10

Flowcytometry for Fig. 4……………………...……..………………..………11-17

Flowcytometry for Fig. 5…………………………..………………………18-23

Flowcytometry for Fig. 6B…………………………..………………………24-27

Flowcytometry for Fig. 6F…………………………..………………………28-29

Flowcytometry for Fig. 8…………………………..………………………….30

Flowcytometry for Fig. 9…………………………..……………………….31-36

Flowcytometry for Fig. 10…………………………..……………………….37-43

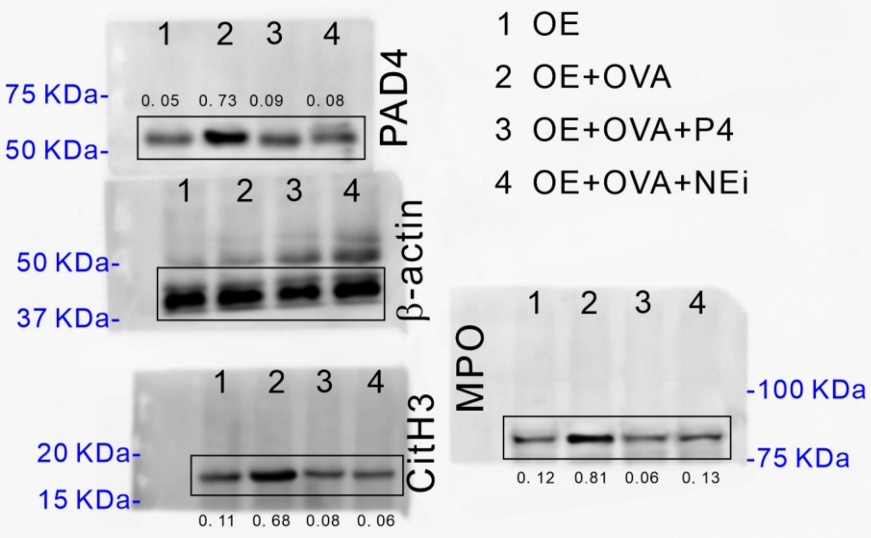

Fig6D_WL_replicate 1, Showing in Figure 6D

Fig6D_WL_replicate 2

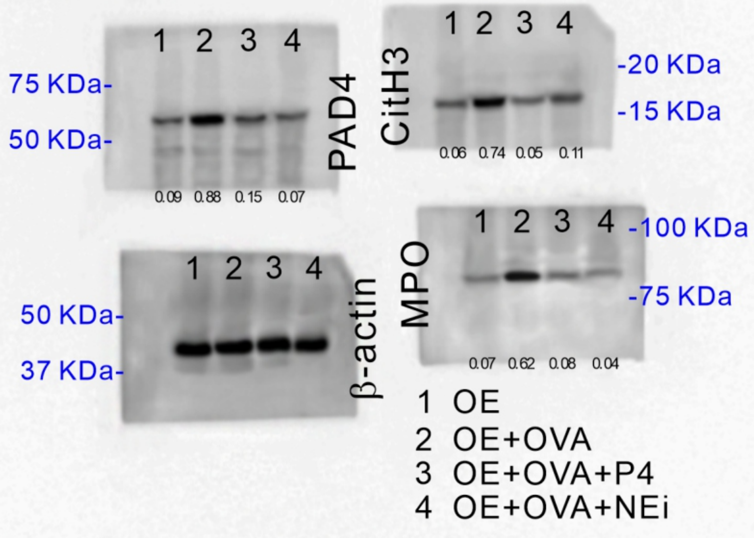

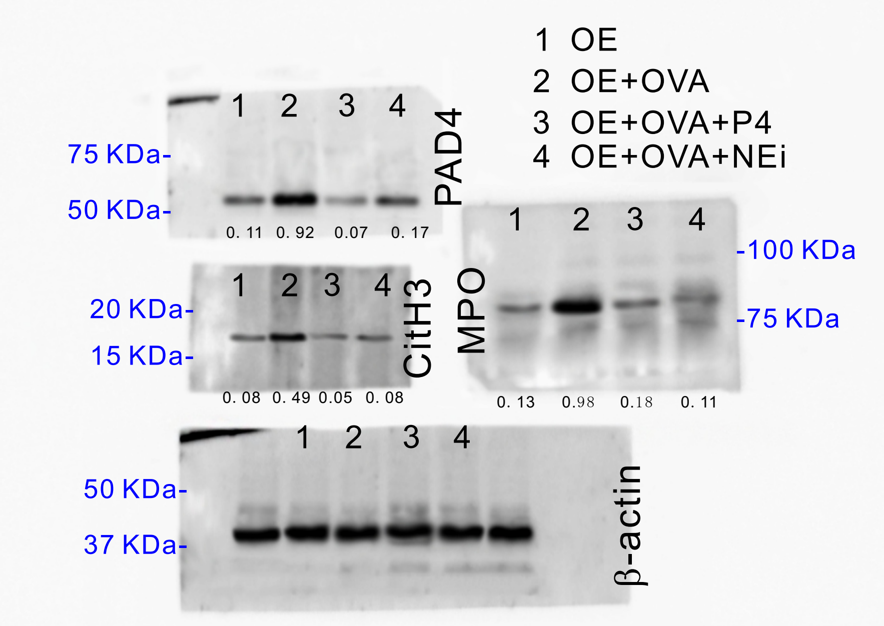

Fig6D_WL_replicate 3

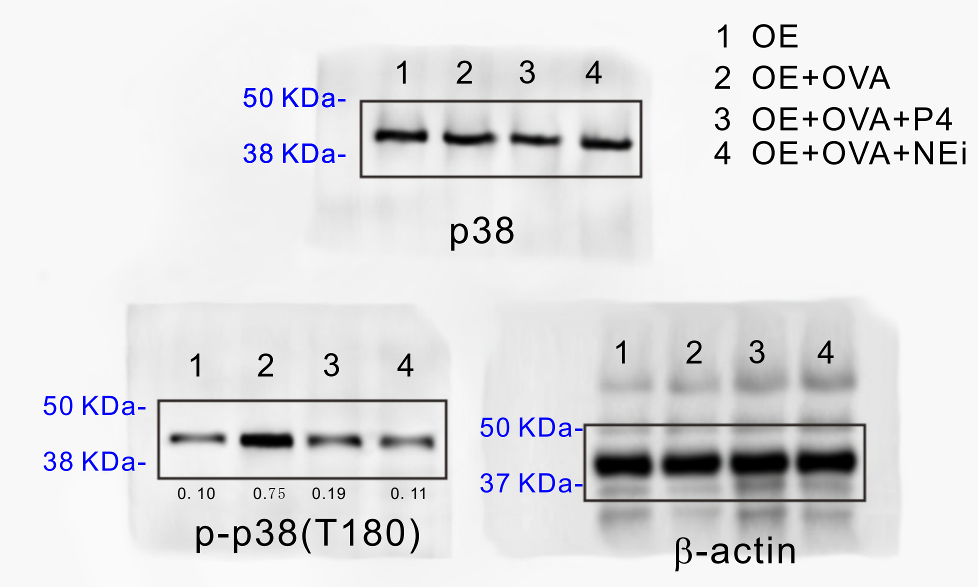

Fig6G_WL_replicate 1, Showing in Figure 6G

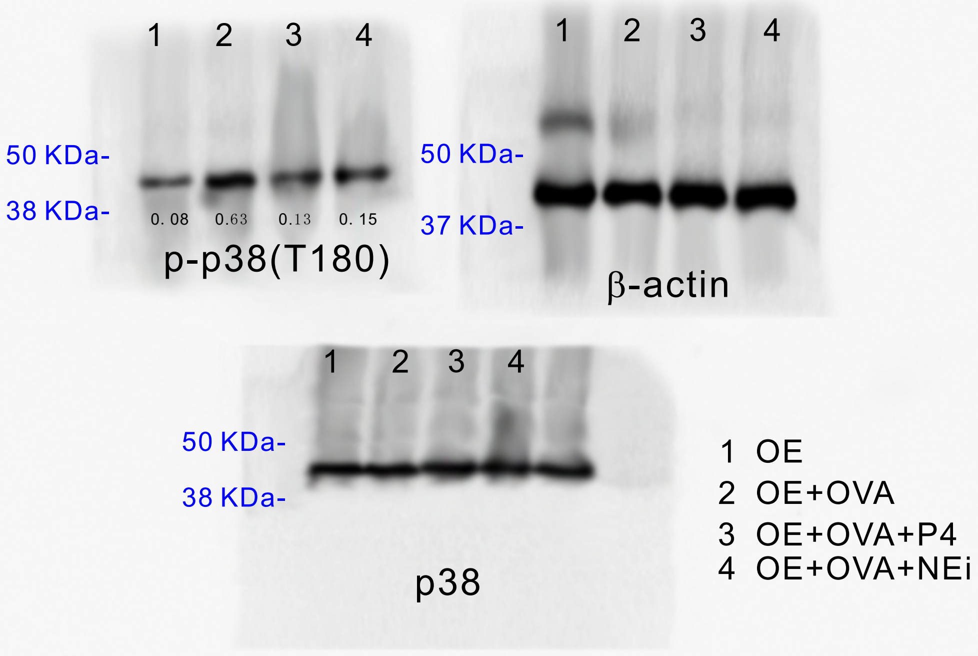

Fig6G_WL_replicate 2

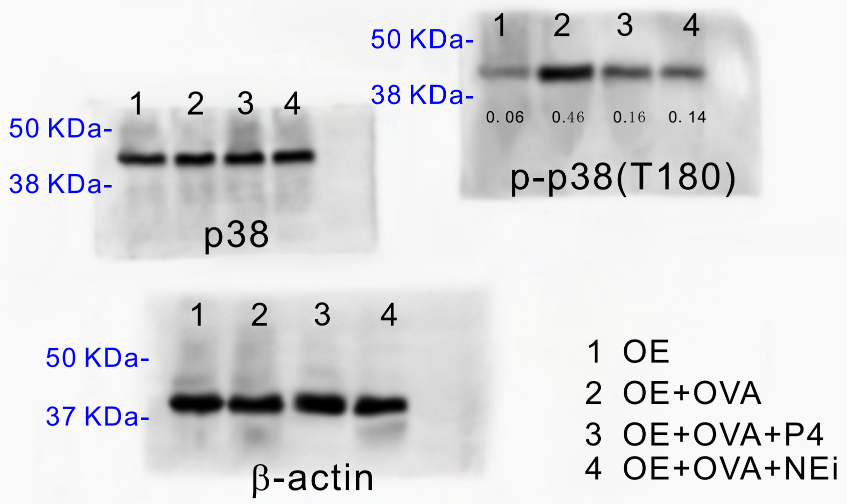

Fig6G_WL_replicate 3

Fig.2F: Flowcytometry: gating strategy

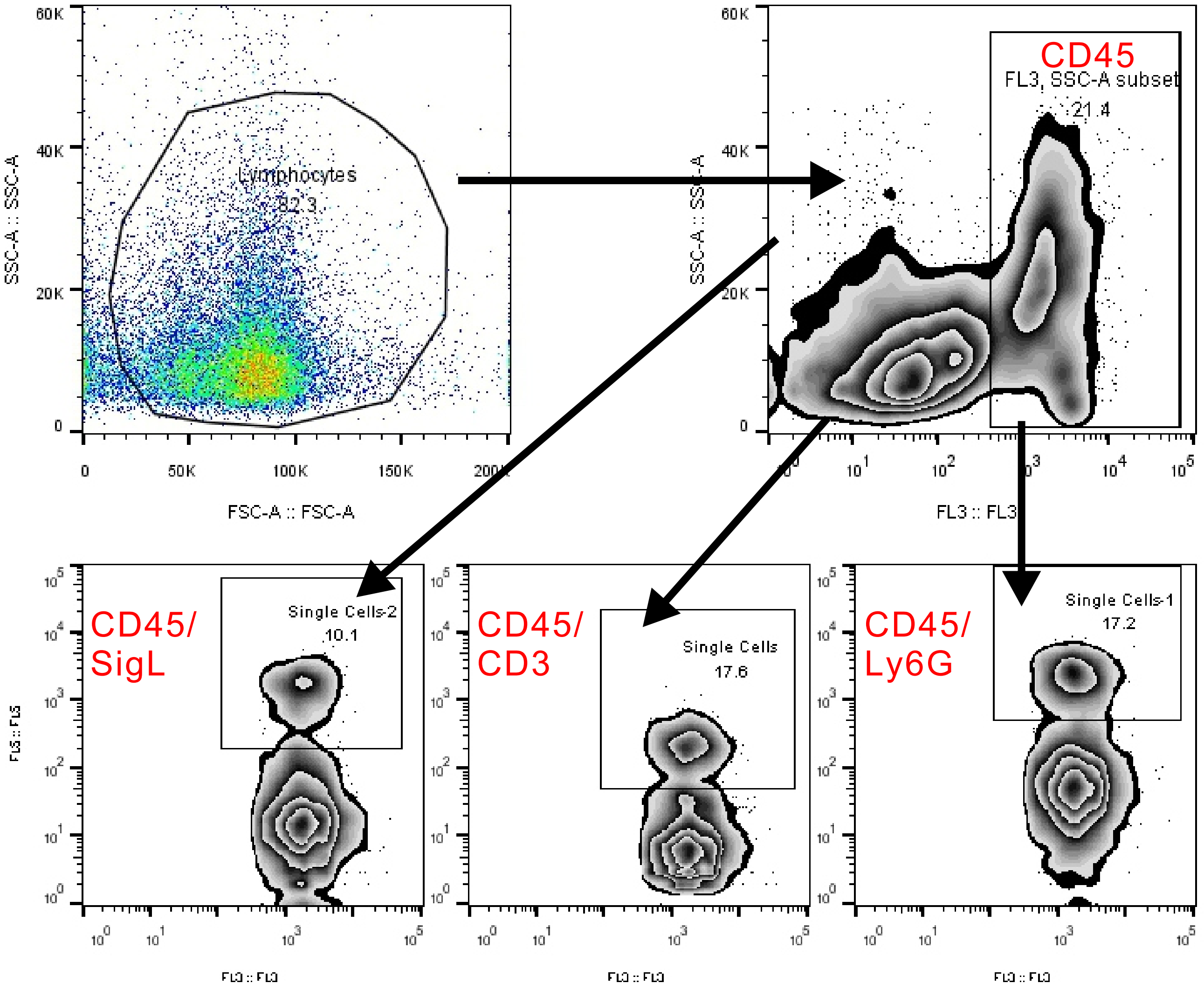

Fig.2F: Flowcytometry FSC::FSC/SSC::SSC

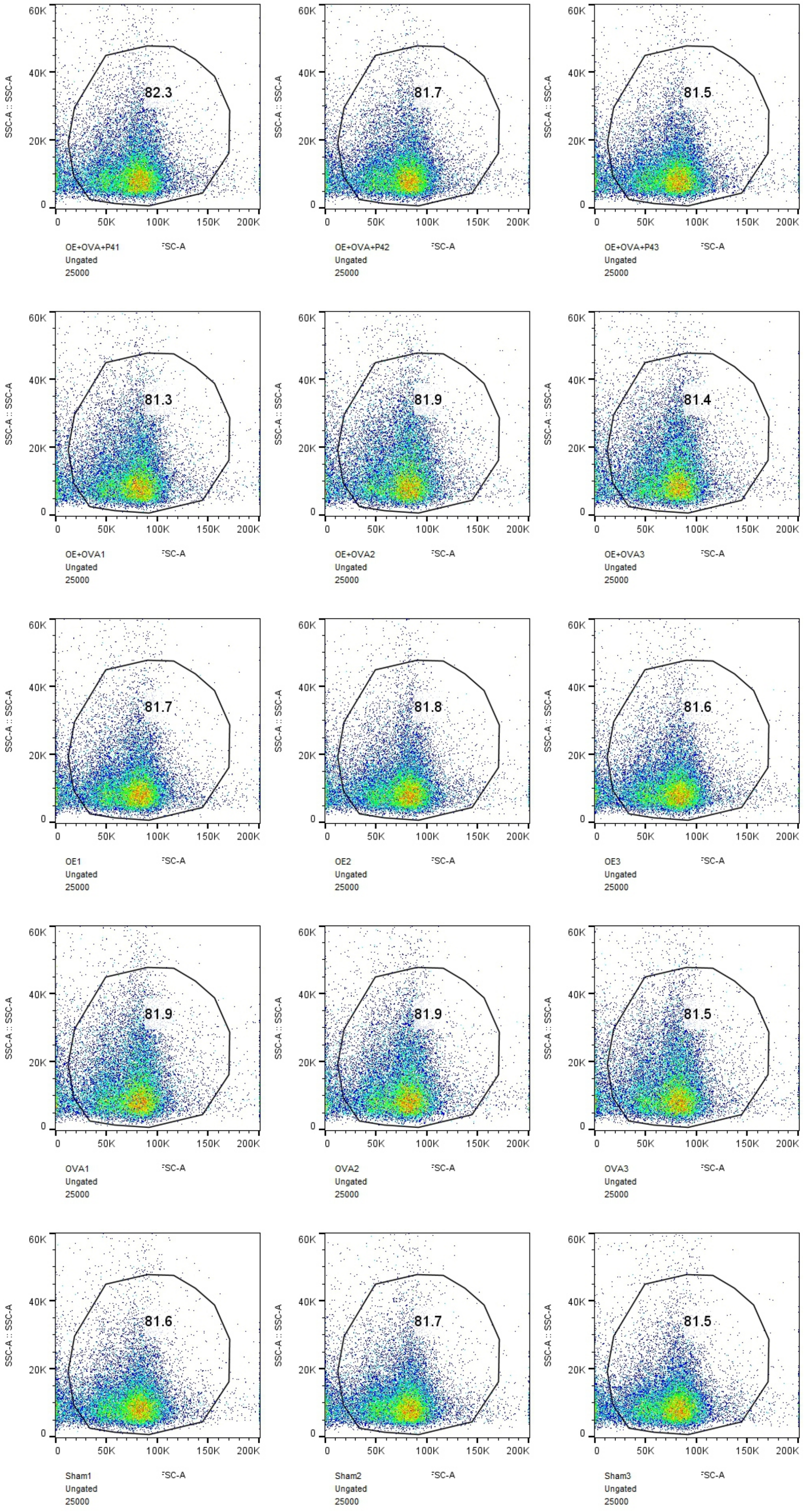

Fig.2F: Flowcytometry FL3::CD45/SSC:SSC

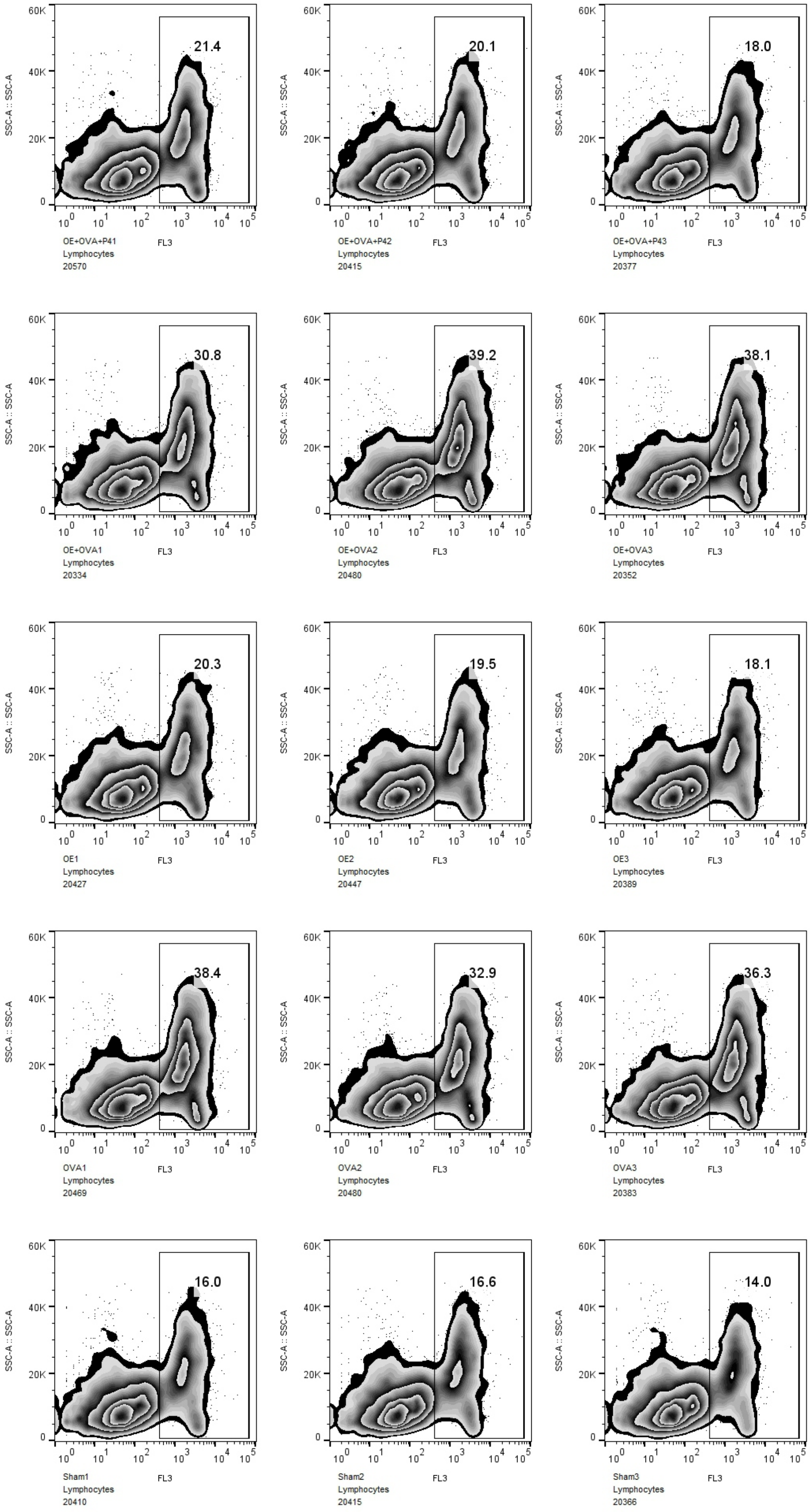

Fig.2F: Flowcytometry FL3::CD45/FL5::SigI

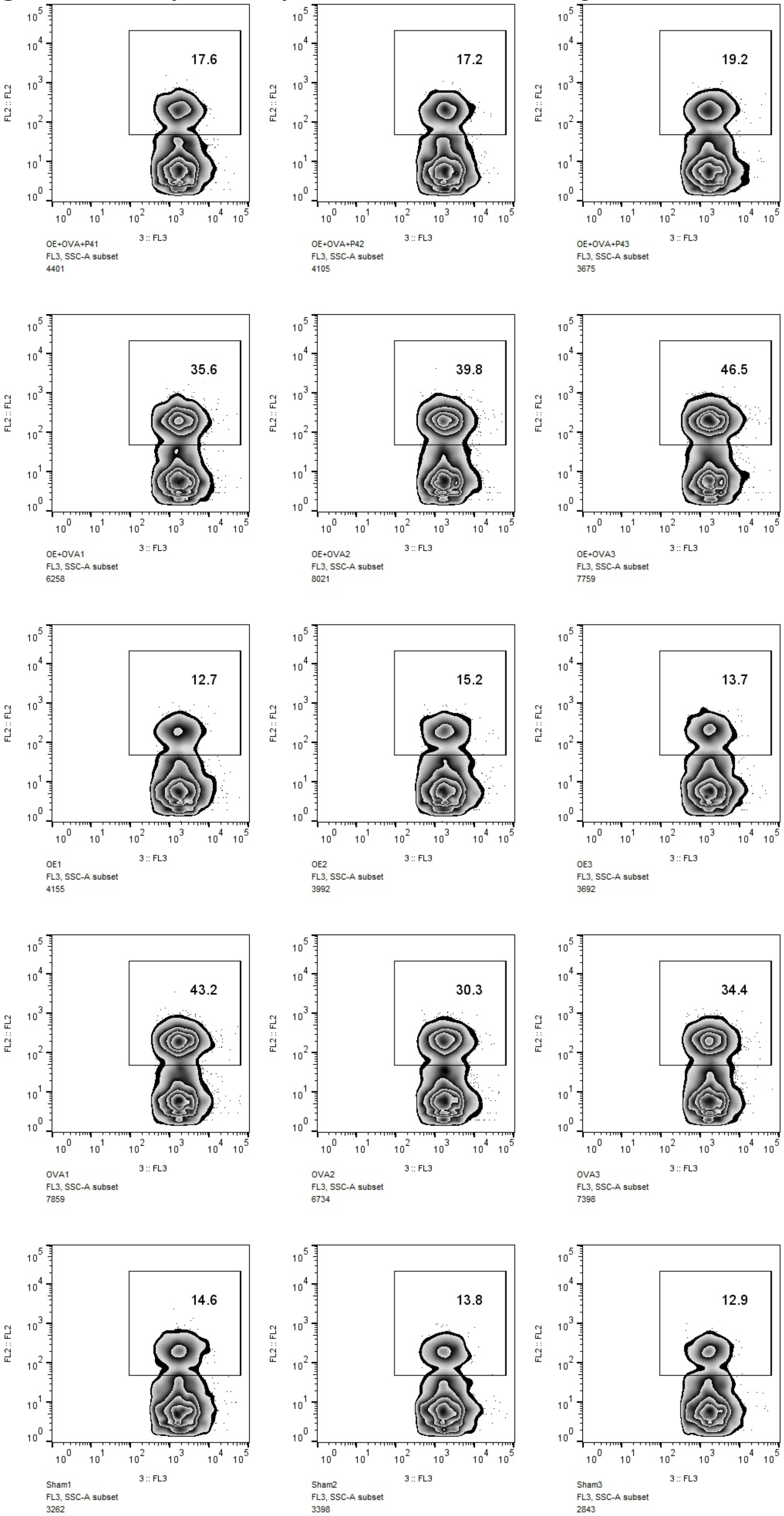

Fig.2F: Flowcytometry FL3::CD45/FL2::CD3

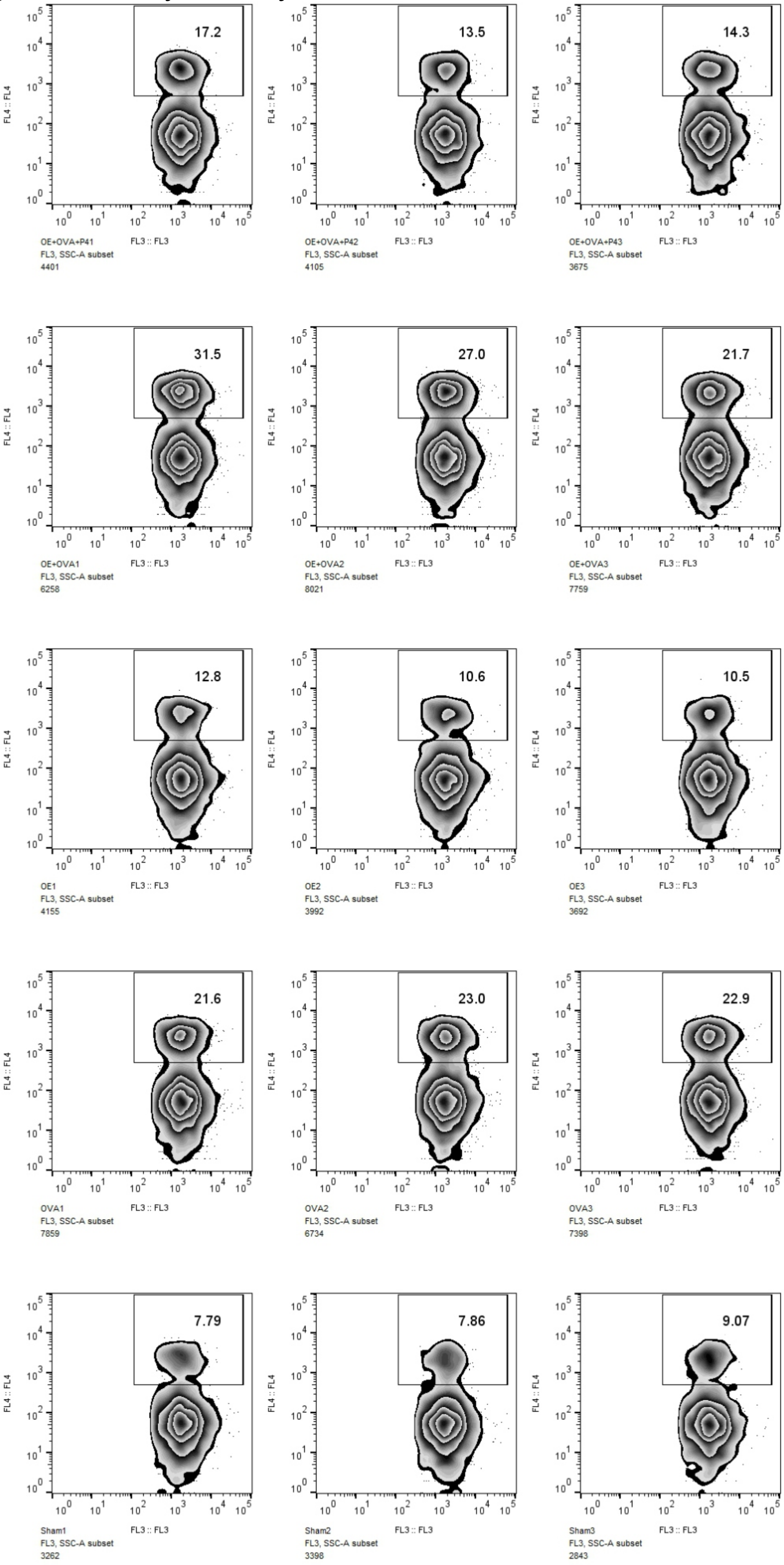

Fig.2F: Flowcytometry FL3::CD45/FL4::Ly6G

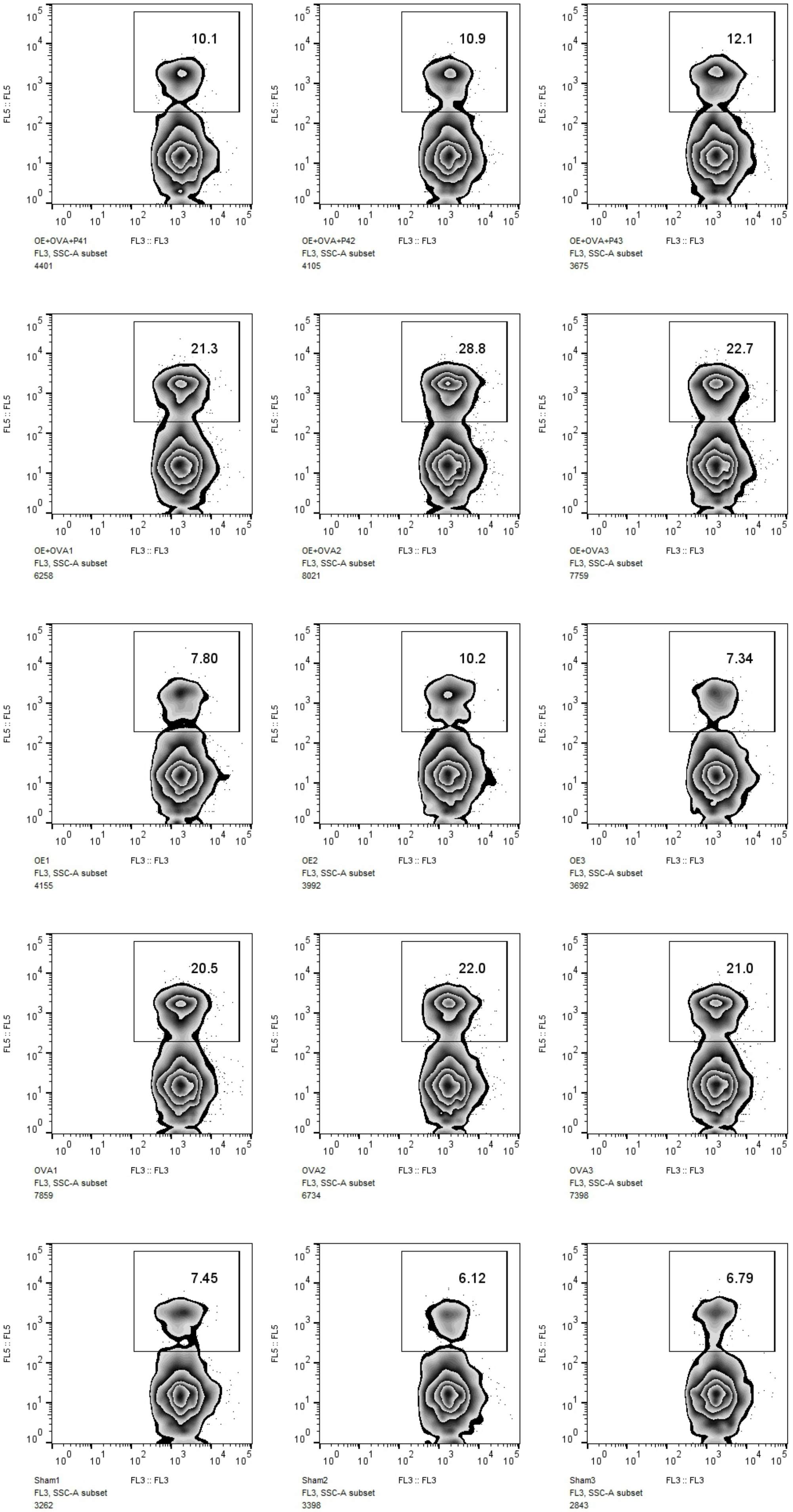

Fig. 4: Flowcytometry: gating strategy for Fig. 4B, C

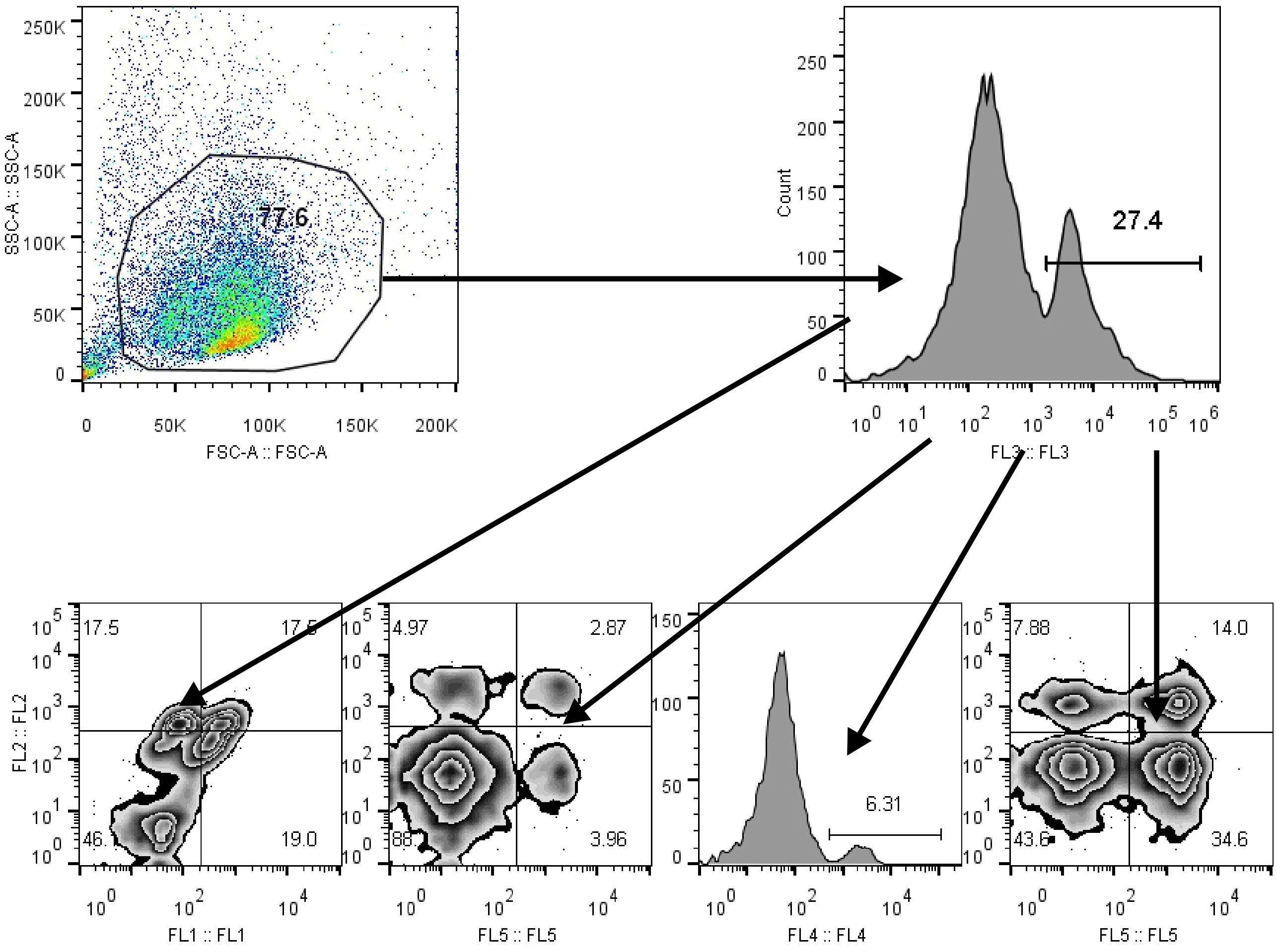

Fig.4A: Flowcytometry FSC/SSC

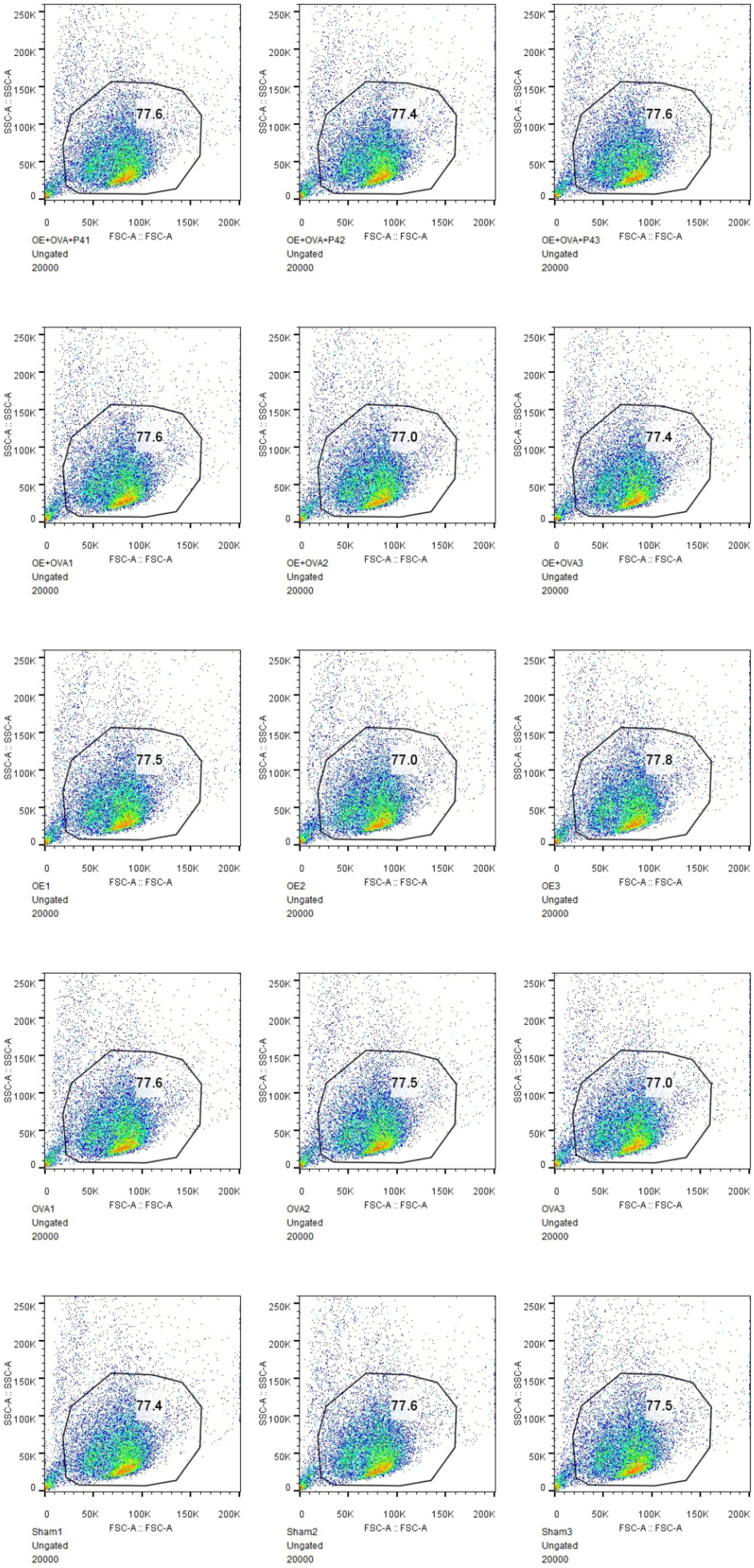

Fig.4A and B: Flowcytometry FSC/FL3::CD45

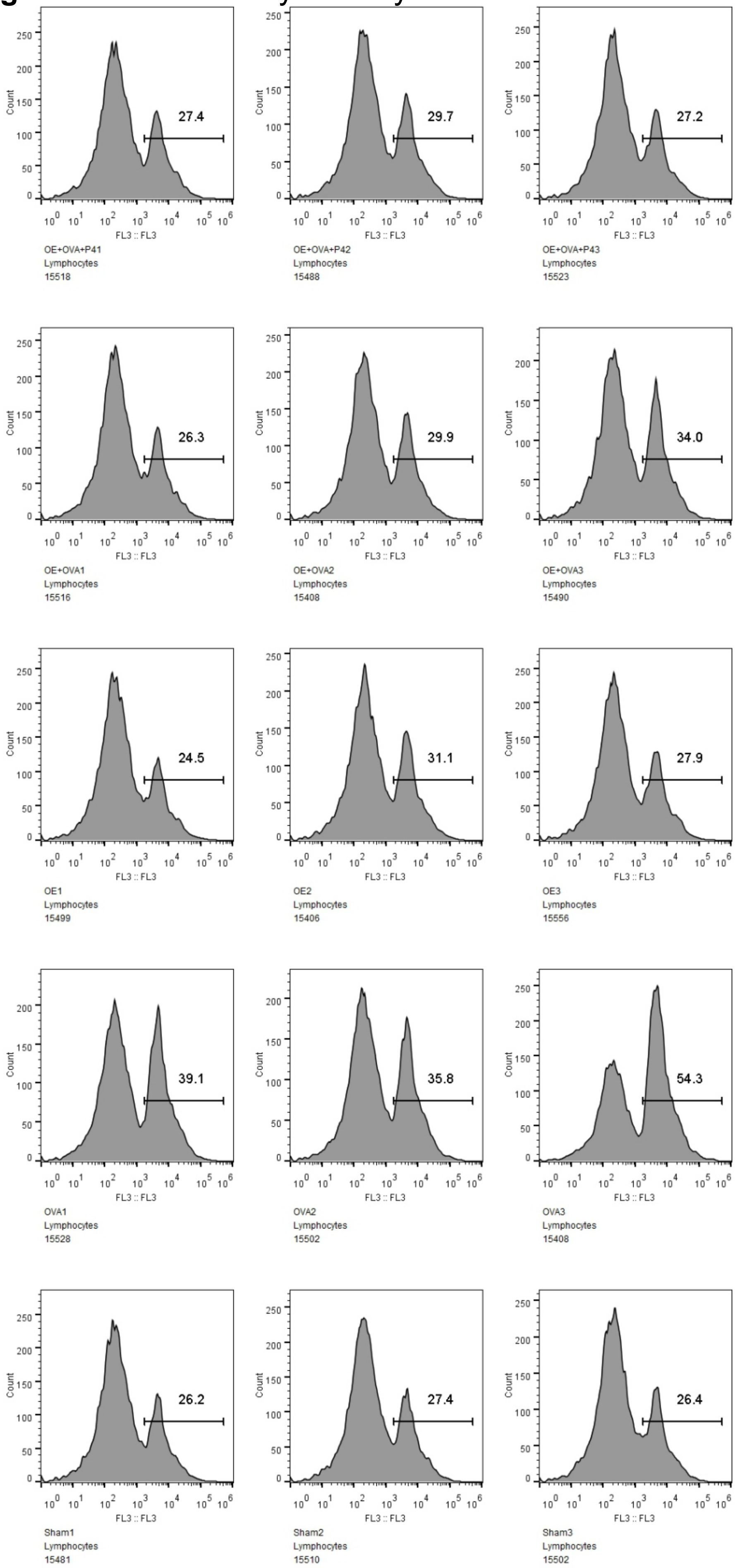

Fig.4A and B: Flowcytometry FL1::IFN-γ/FL2::IL-2

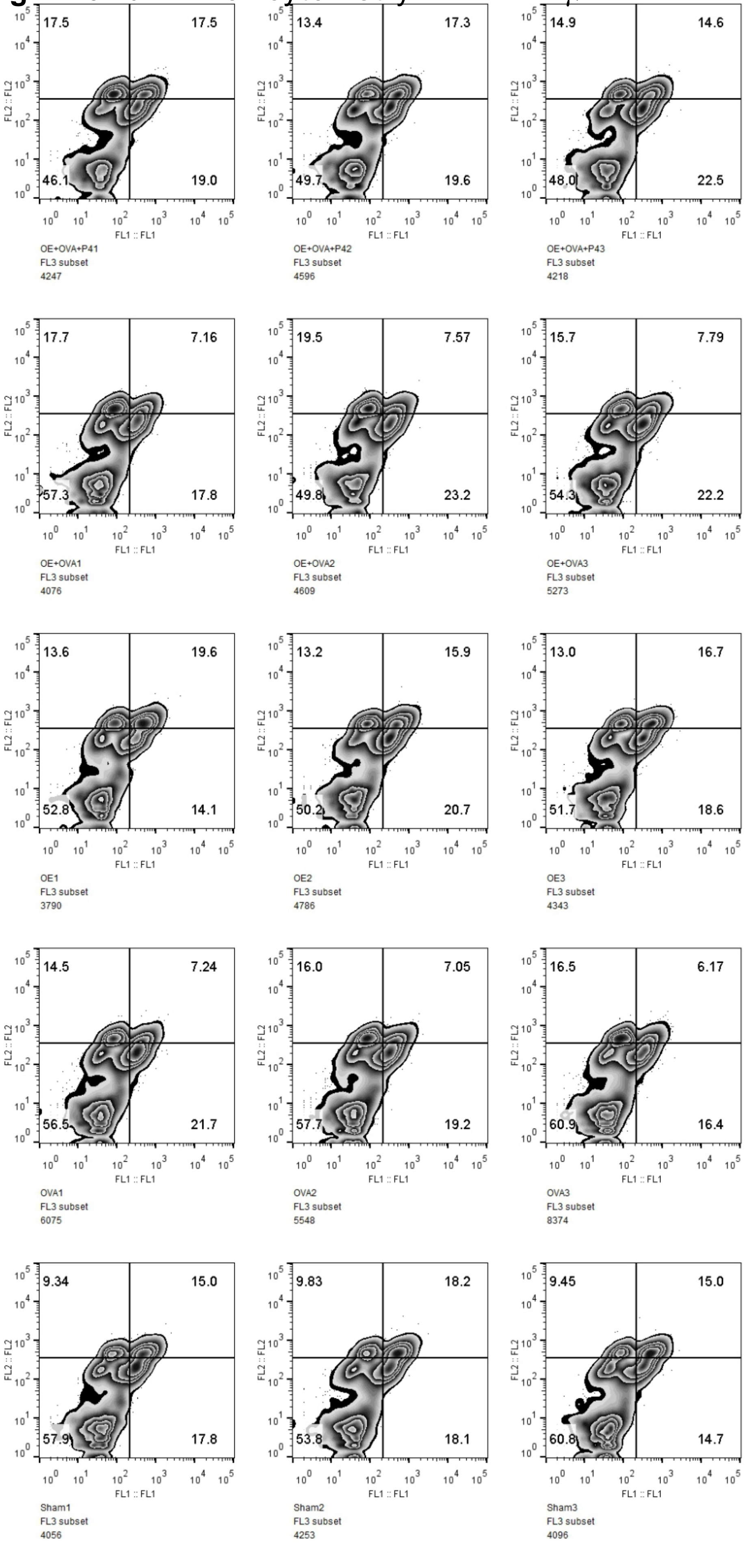

Fig.4A and B: Flowcytometry FL5::IL-4/FL4::IL-5

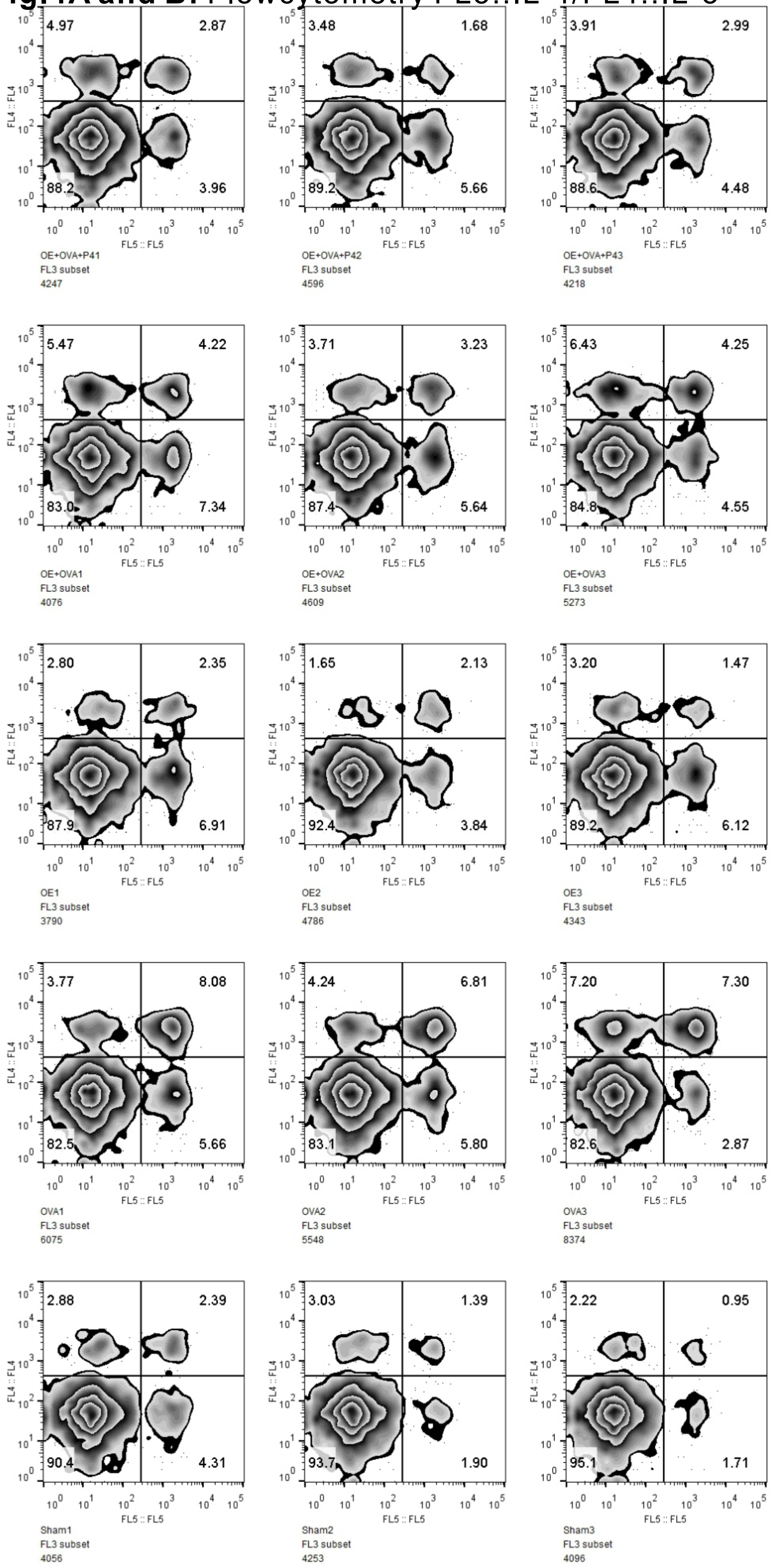

Fig.4 A and C Flowcytometry: SSC/FL4::IL-17

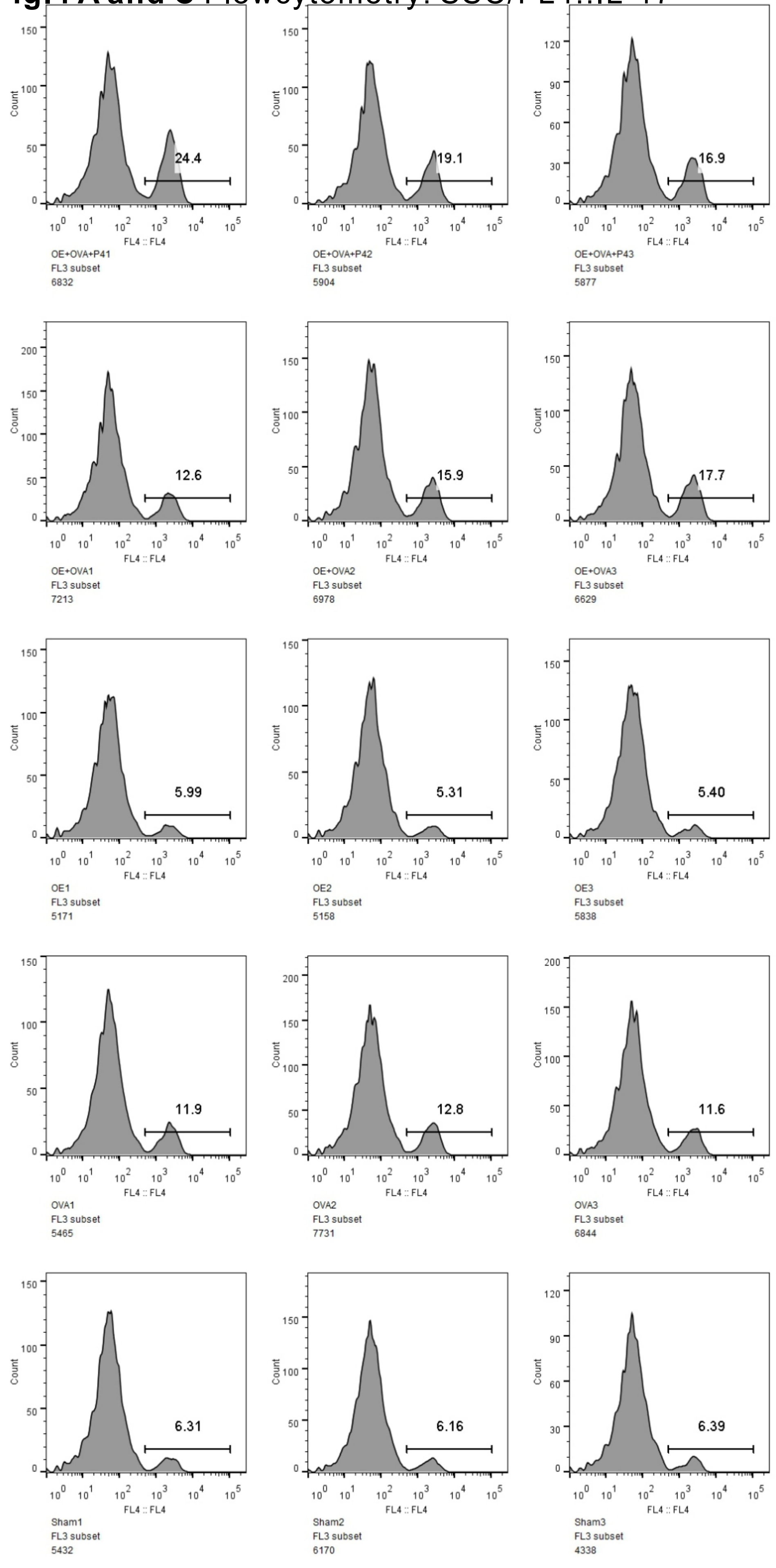

Fig.4A and C Flowcytometry: FL5::IL-10/FL2::TGF-β1

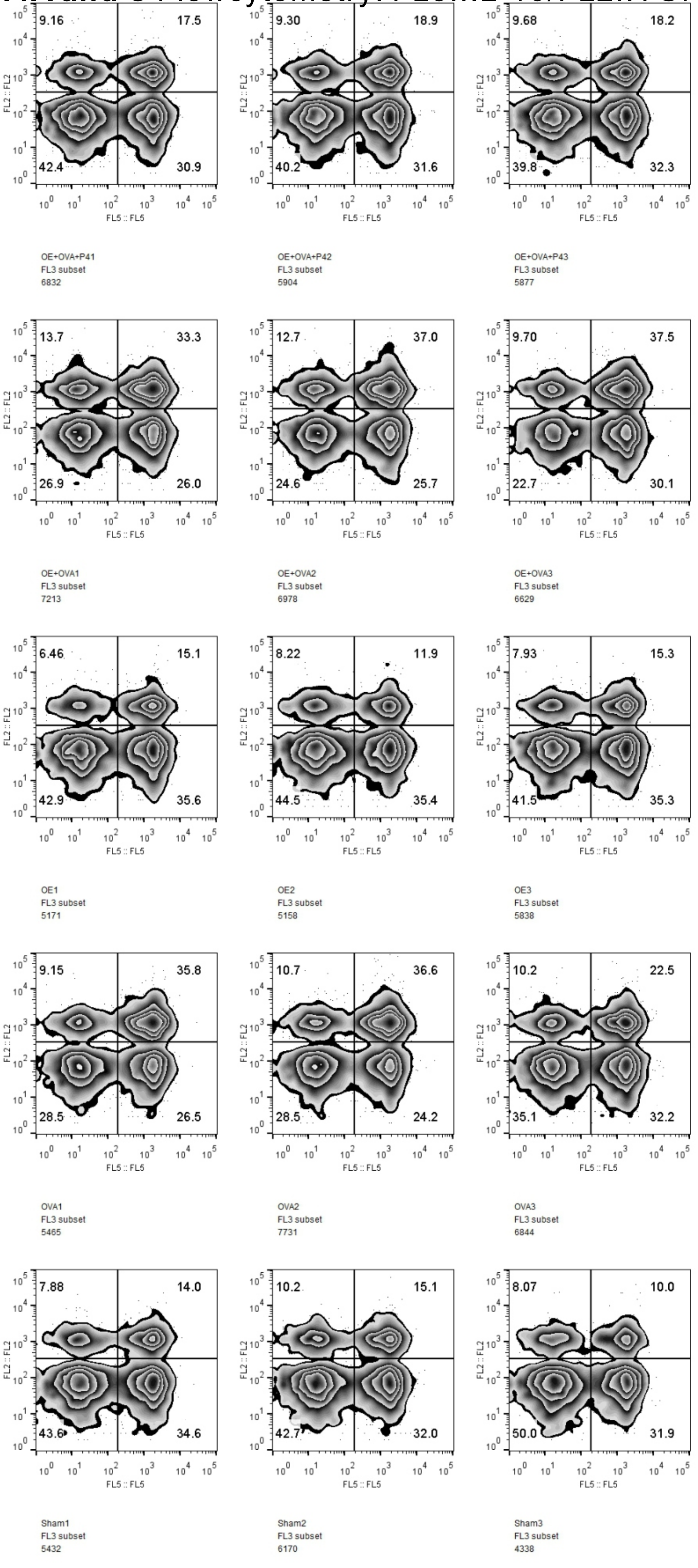

Fig.5A: Flowcytometry: gating strategy for Fig.5B, C

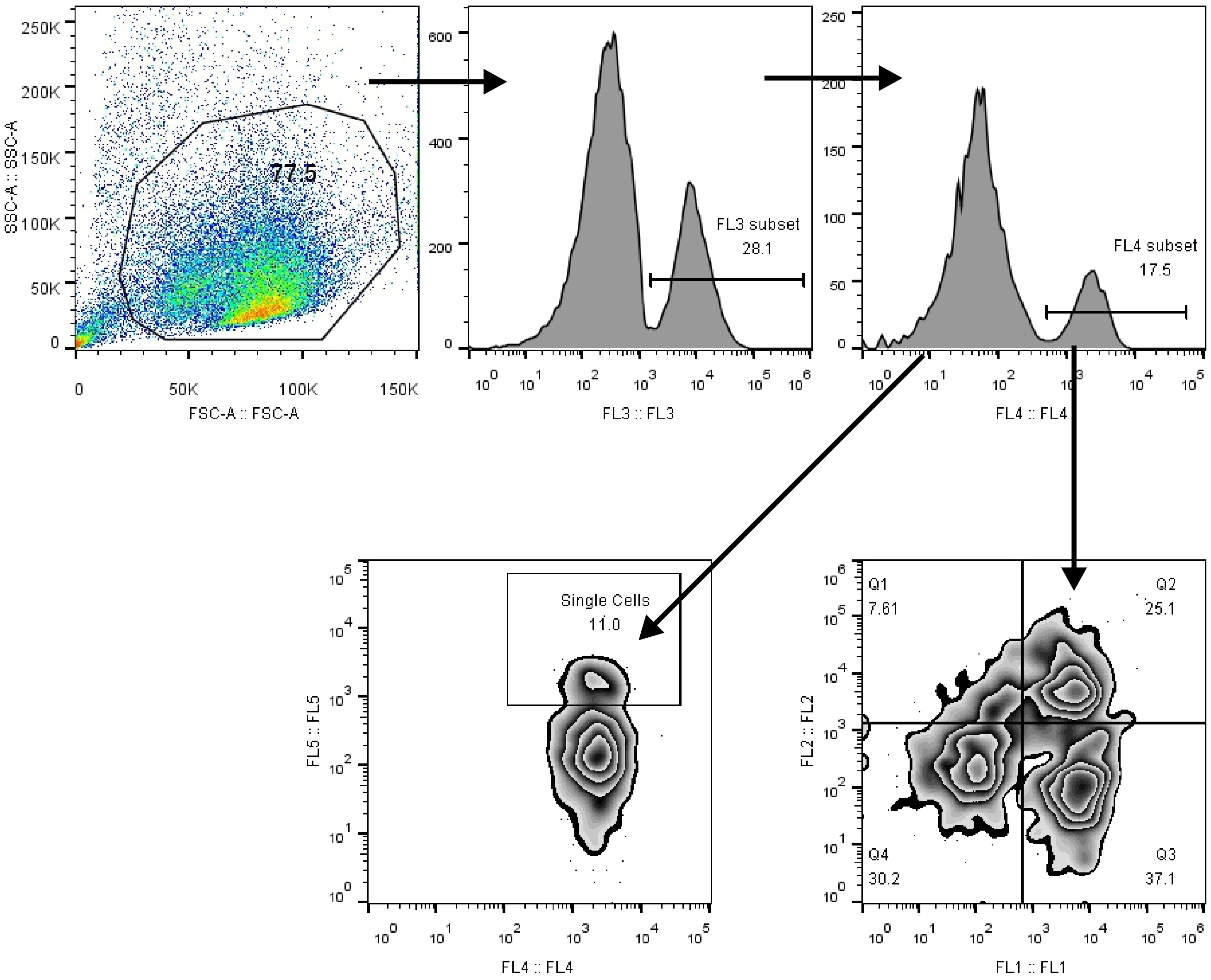

Fig.5A: Flowcytometry FSC::FSC/SSC::SSC

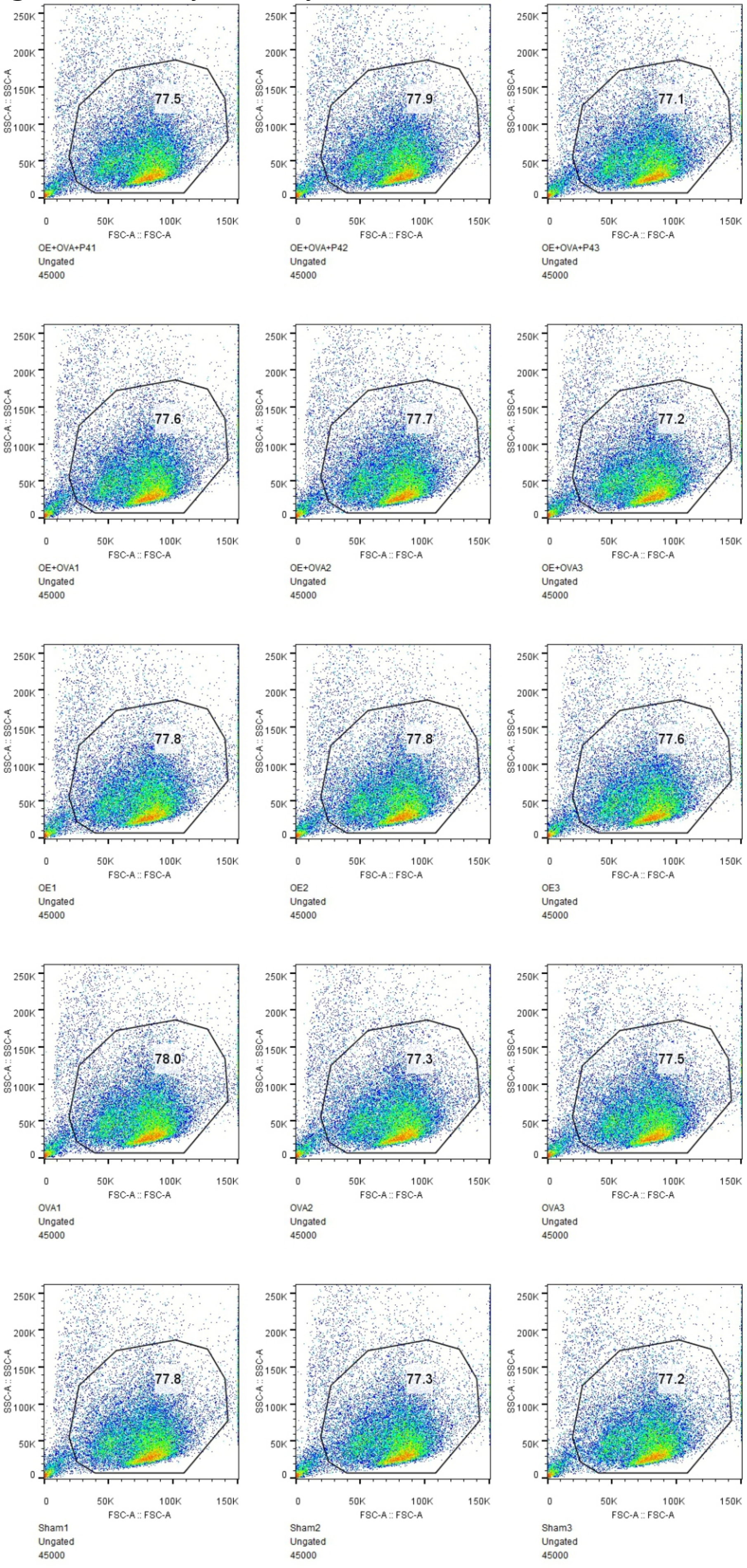

Fig.5A: Flowcytometry SSC::SSC/FL3::CD45

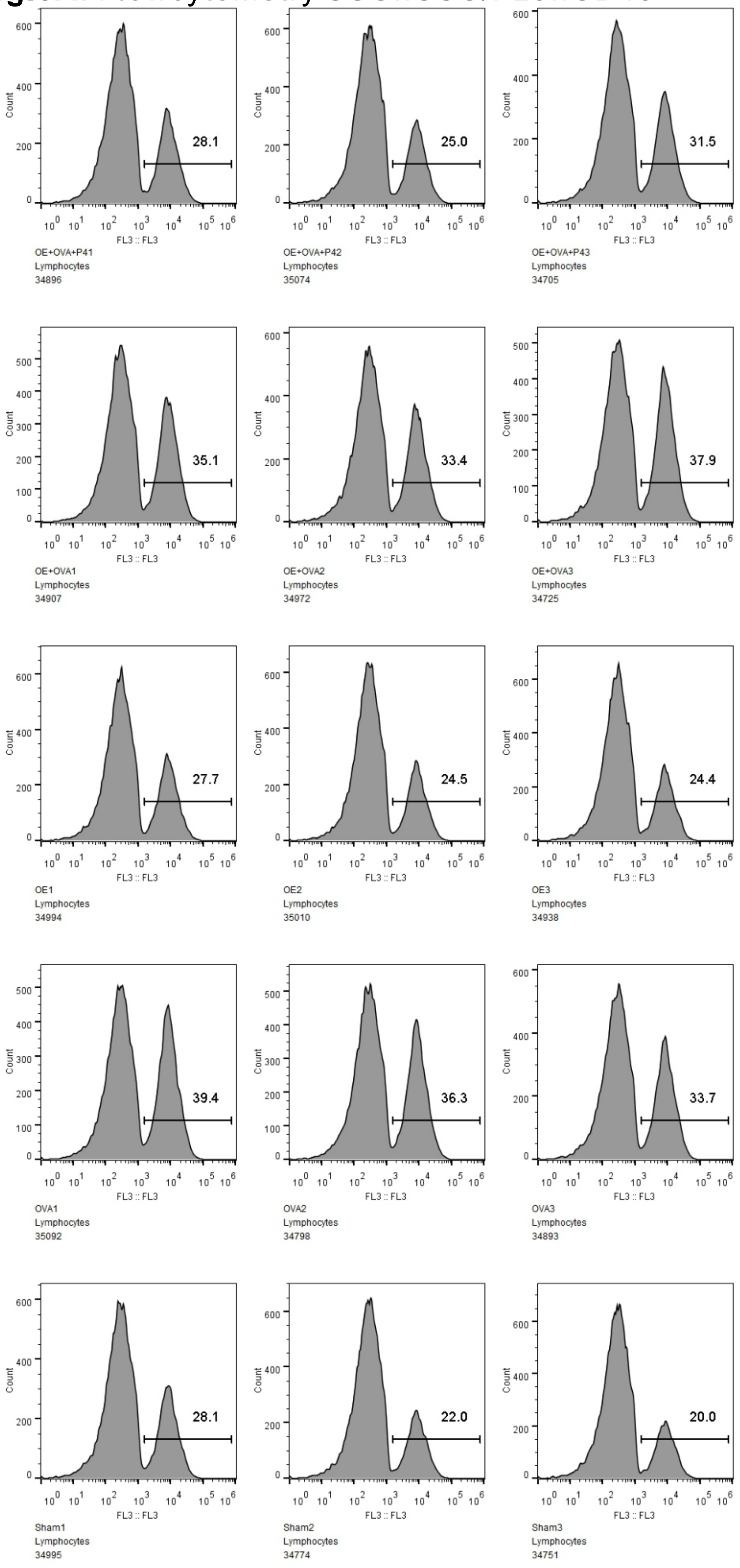

Fig.5A: Flowcytometry SSC::SSC/FL4::CD4

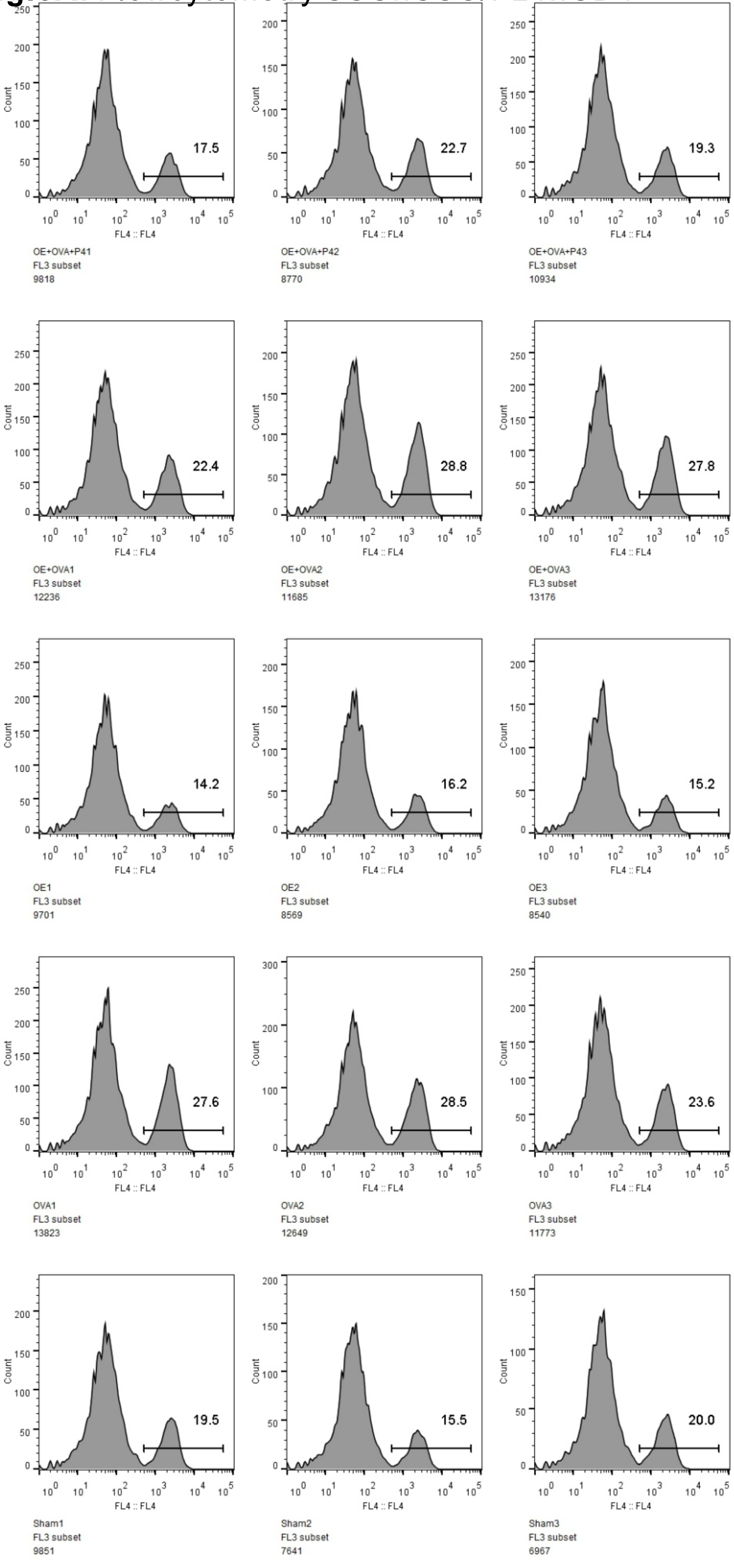

Fig.5A and B: Flowcytometry FL4::CD4/FL5::CD44

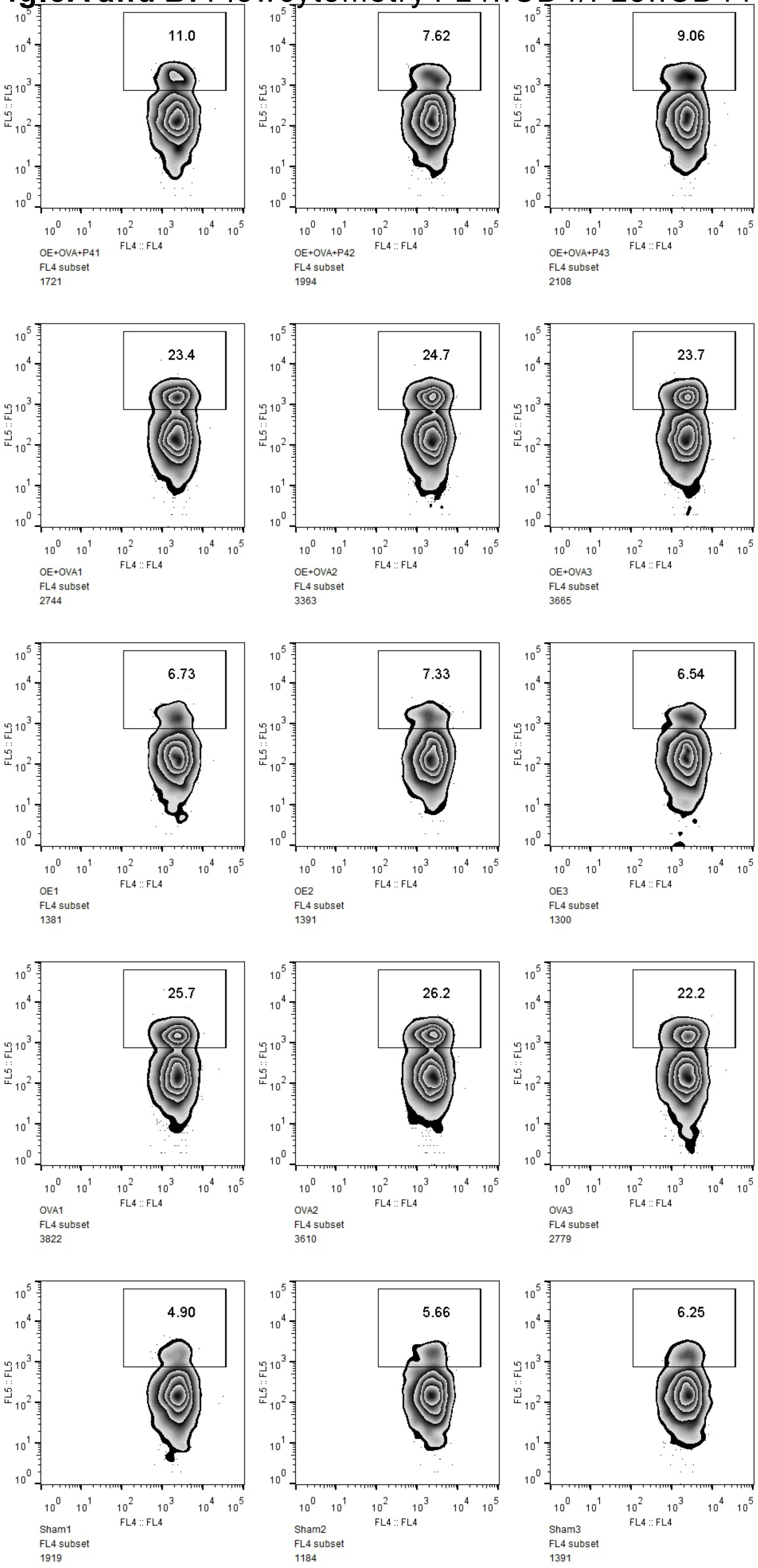

Fig.5A and C: Flowcytometry FL1::Foxp3/FL2::CD25

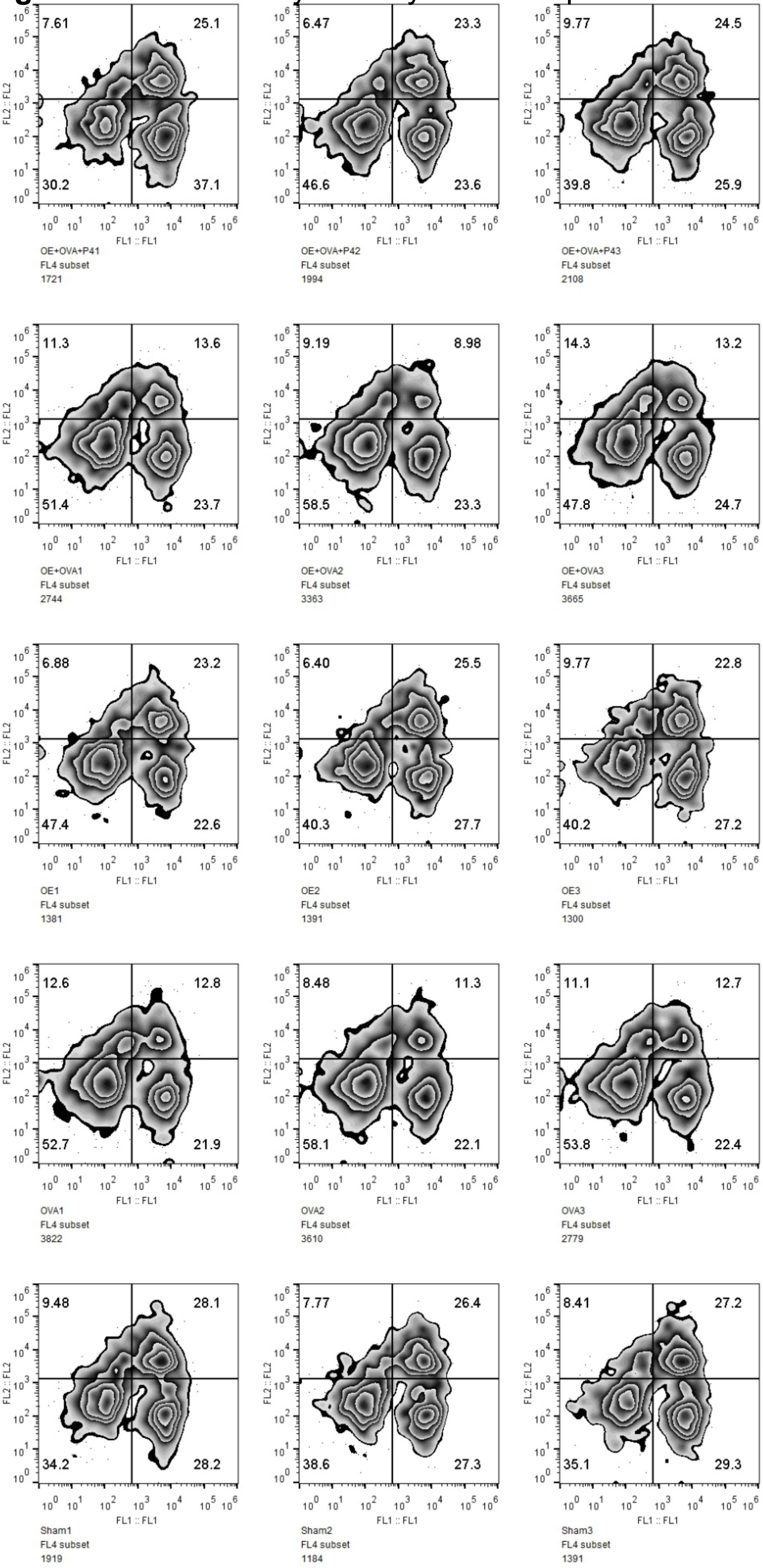

Fig.6B: Flowcytometry: gating strategy for Fig.6B

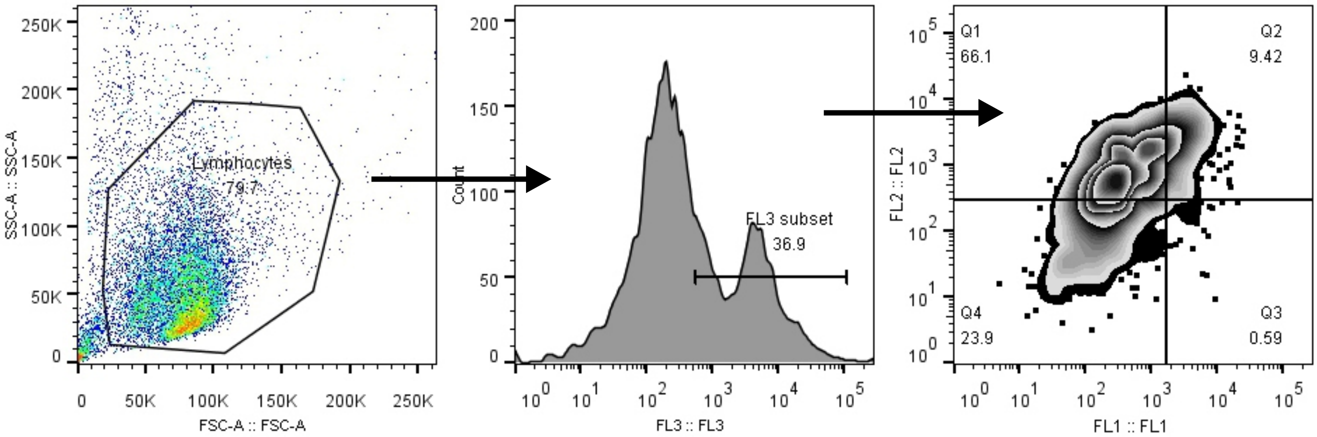

Figure 6B FSC and SSC gating

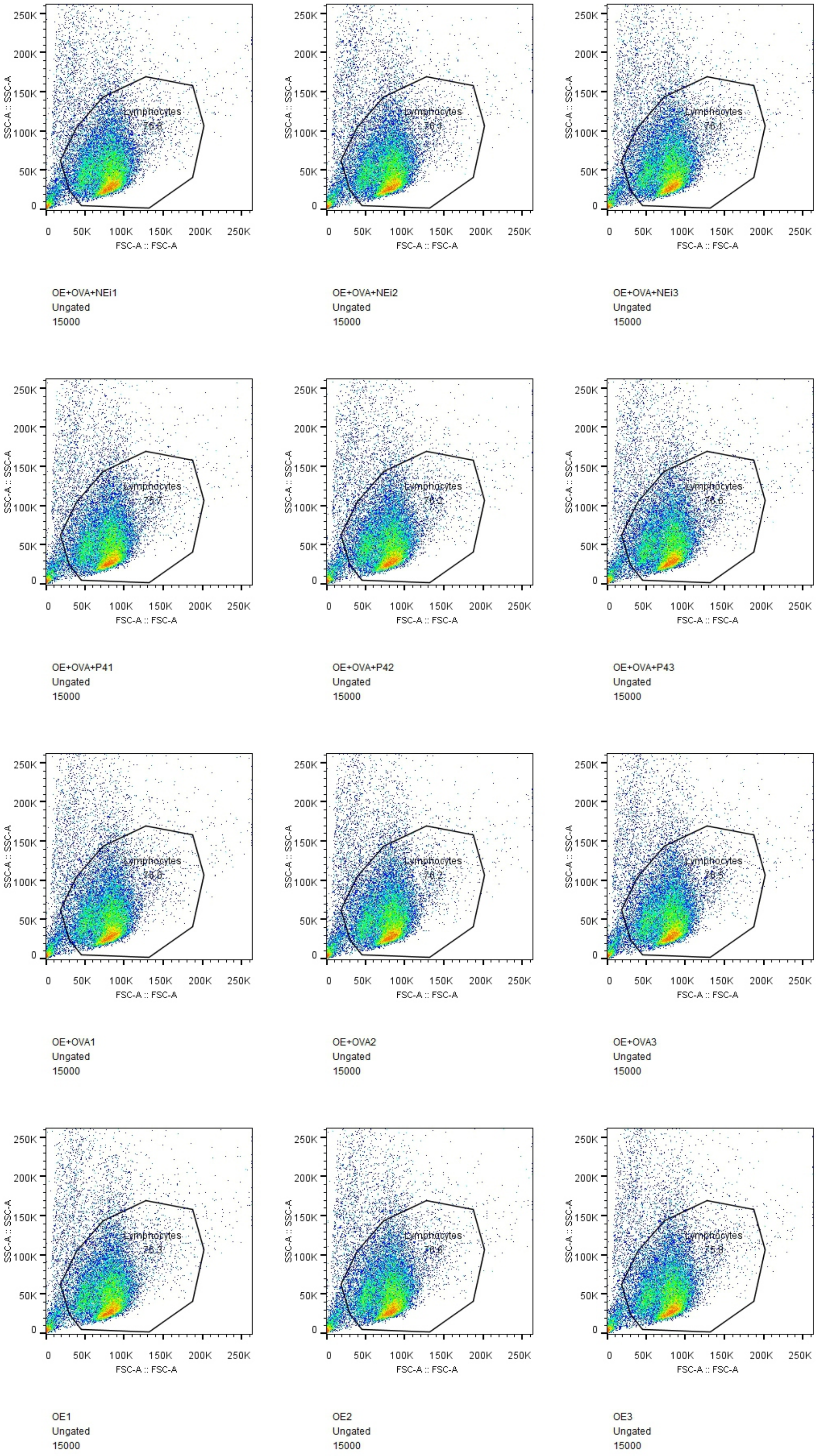

Figure 6B FL3 CD45 gating

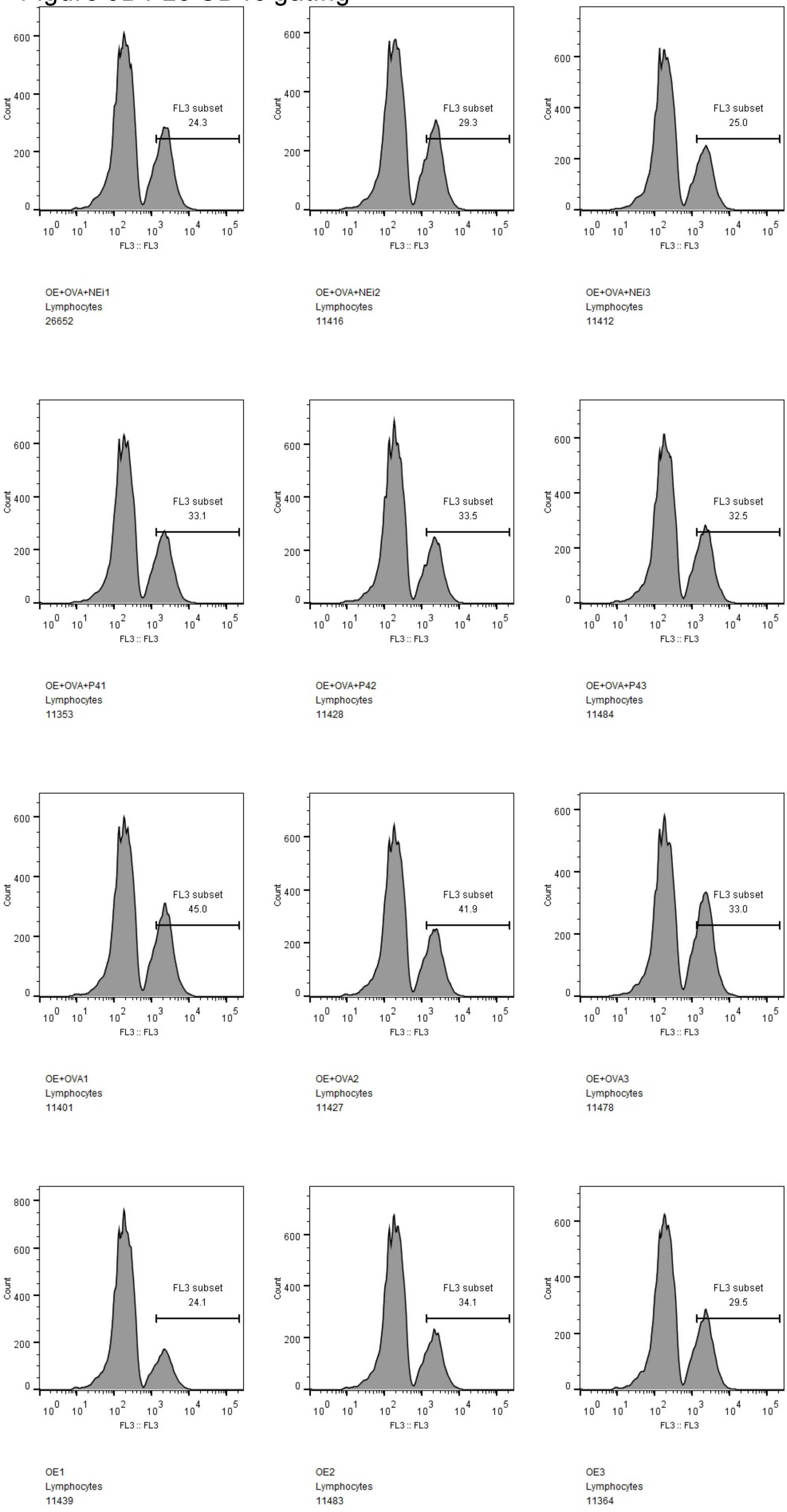

Figure 6B FL1/FL2 CD45+CD11b+Ly6G+

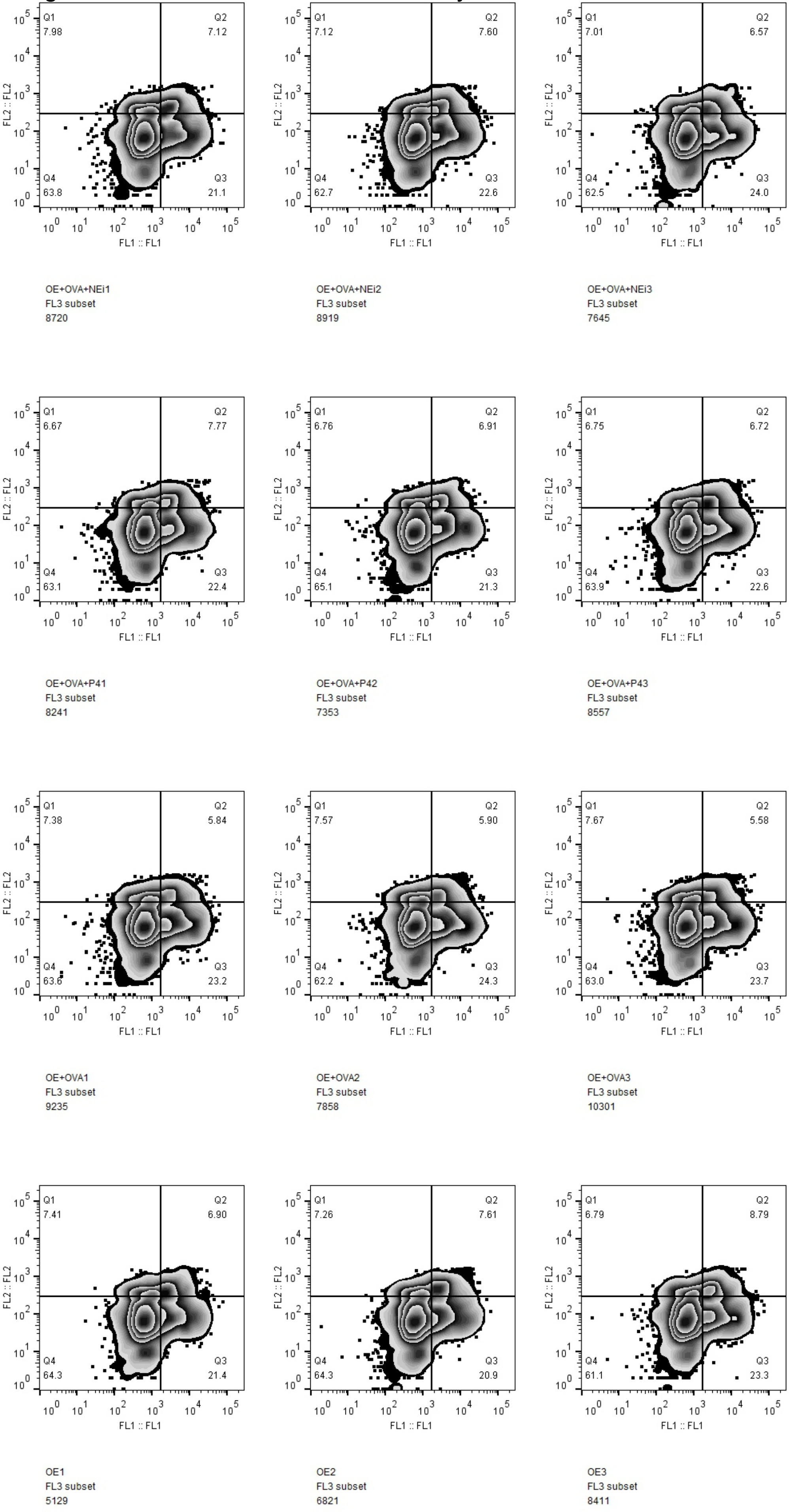

Figure 6F FSC and SSC

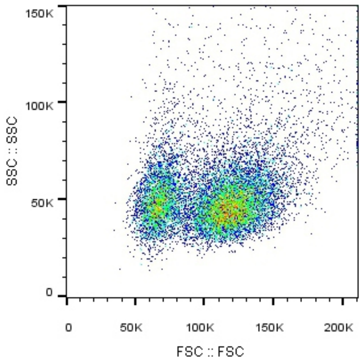

OE_OVA_NEi1
Ungated
15000

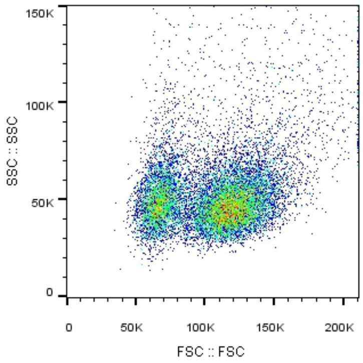

OE_OVA_NEi2
Ungated
15000

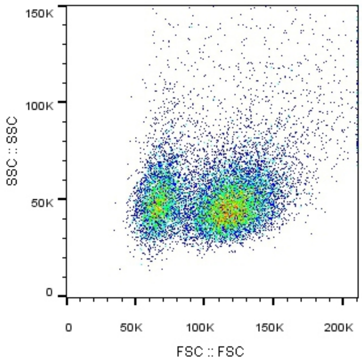

OE_OVA_NEi3
Ungated
15000

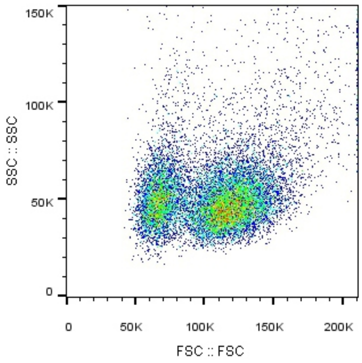

OE_OVA_P41
Ungated
15000

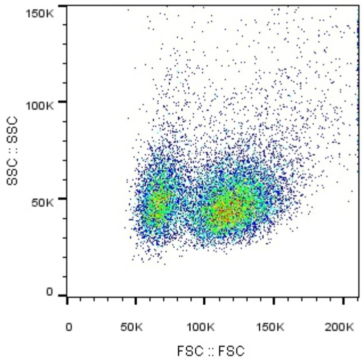

OE_OVA_P42
Ungated
15000

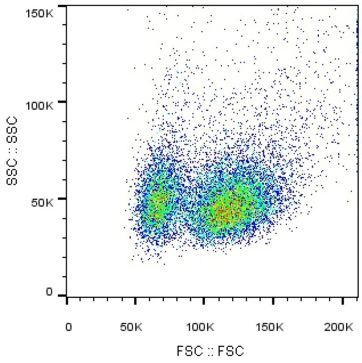

OE_OVA_P43
Ungated
15000

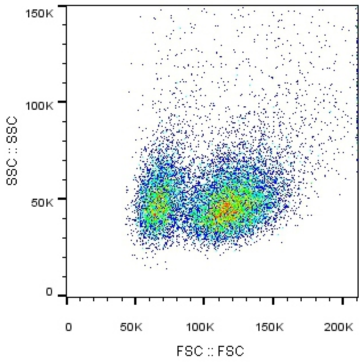

OE_OVA1
Ungated
15000

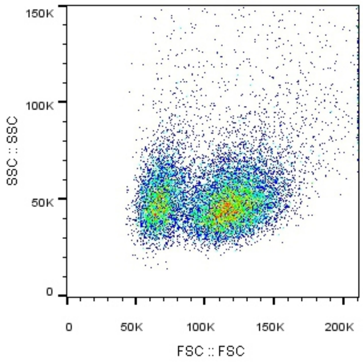

OE_OVA2
Ungated
15000

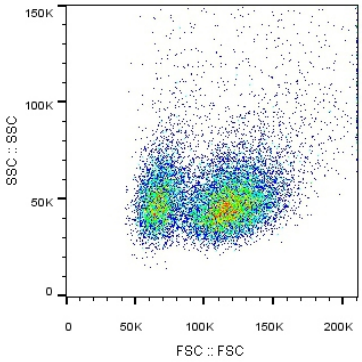

OE_OVA3
Ungated
15000

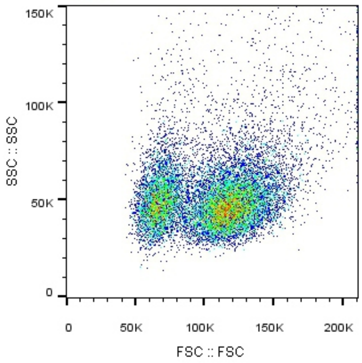

OE1
Ungated
15000

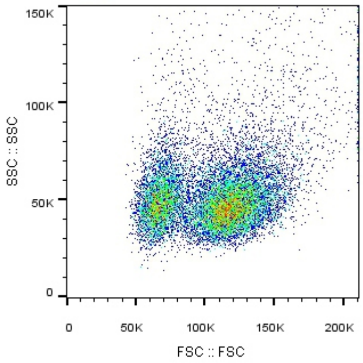

OE2
Ungated
15000

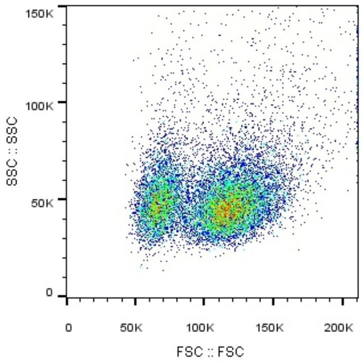

OE3
Ungated
15000

Figure 6F

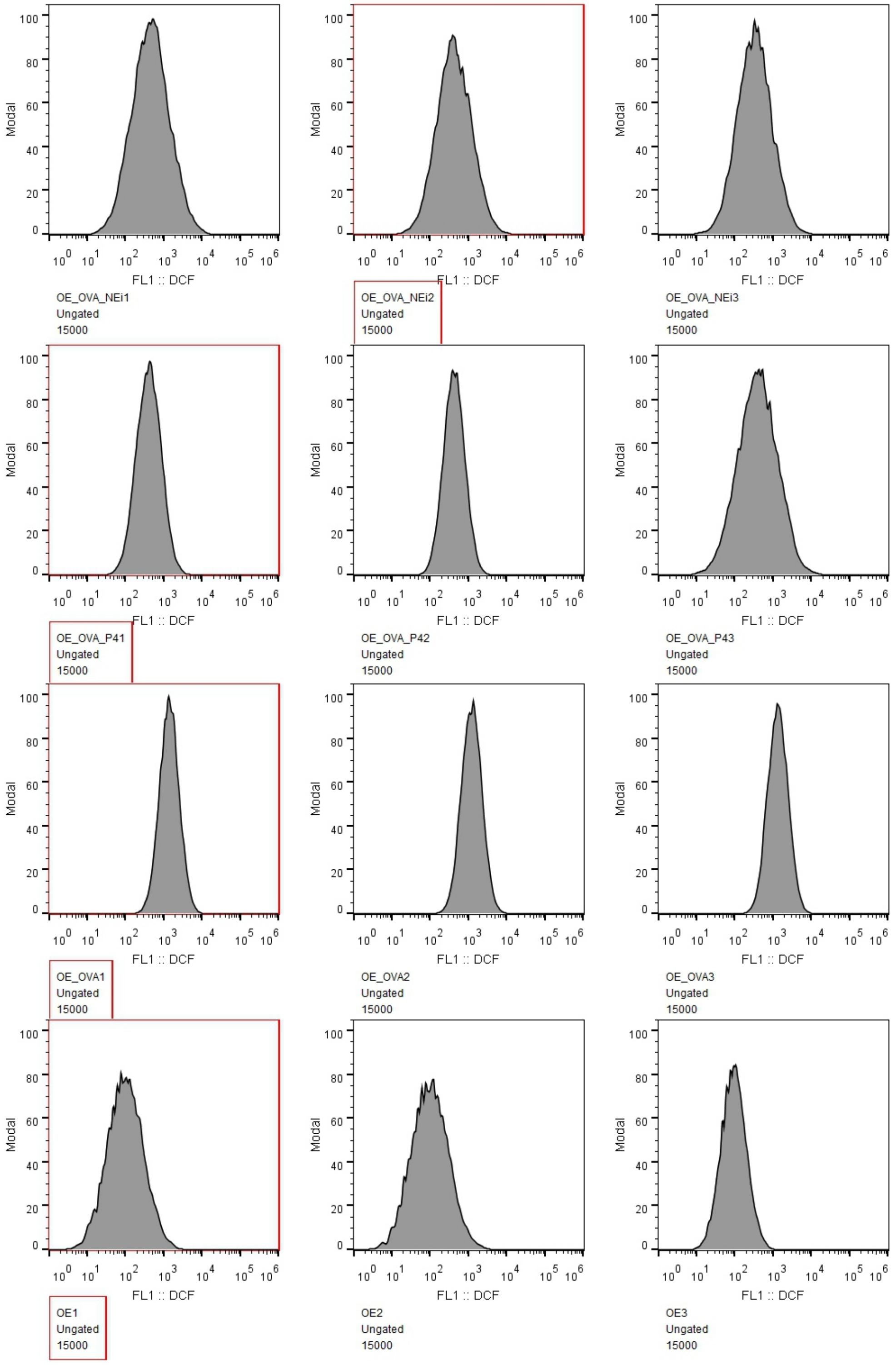

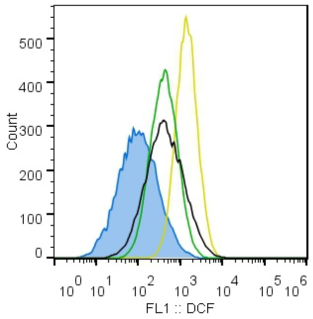

	Sample Name	Subset Name	Count
☐	OE_OVA_NEi2	Ungated	15000
☐	OE_OVA_P41	Ungated	15000
☐	OE_OVA1	Ungated	15000
☐	OE1	Ungated	15000

Figure 8. Gating strategy CD4 for Figure 9

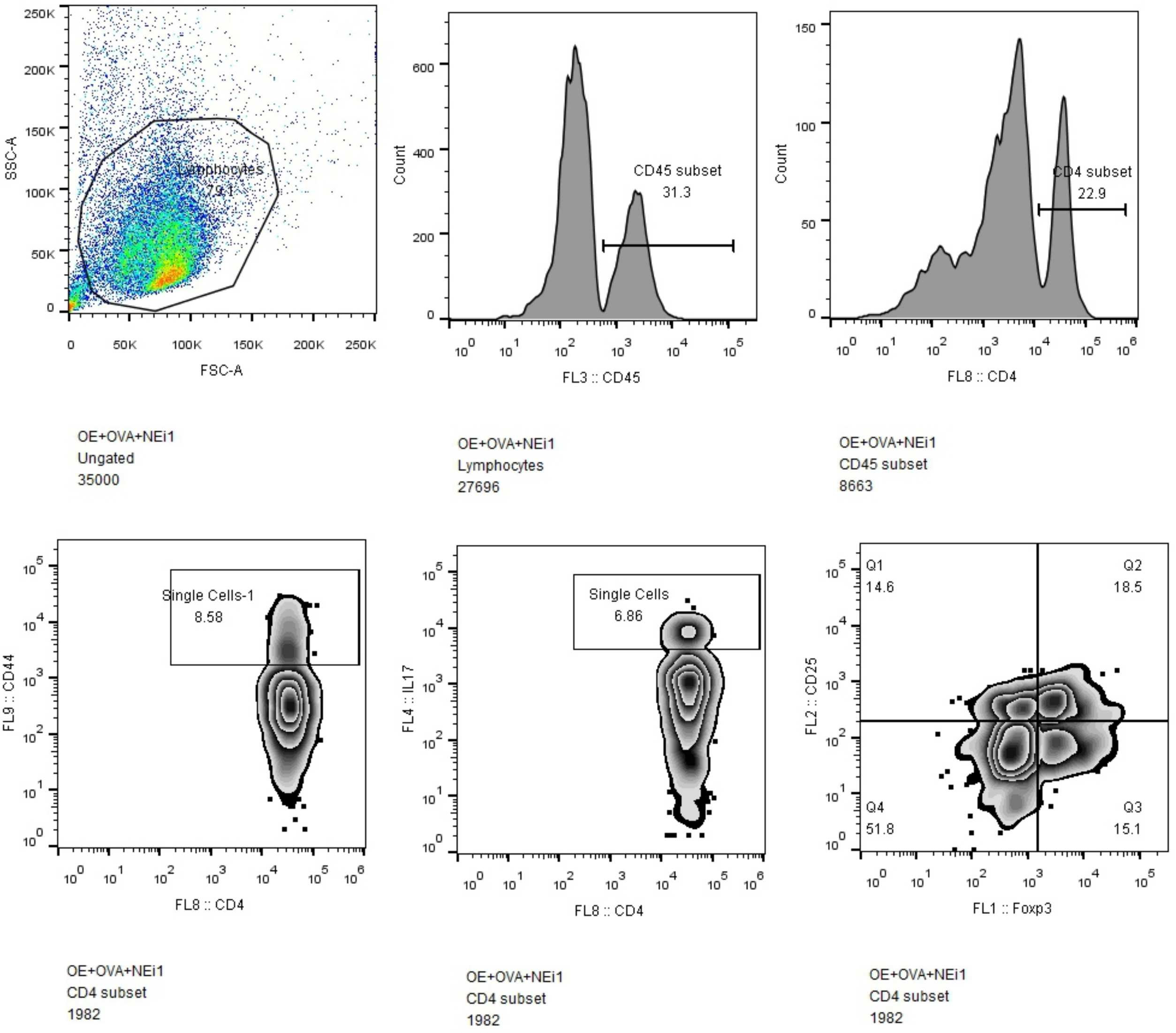

Figure 8. FSC/SSC Gating for Figure 9

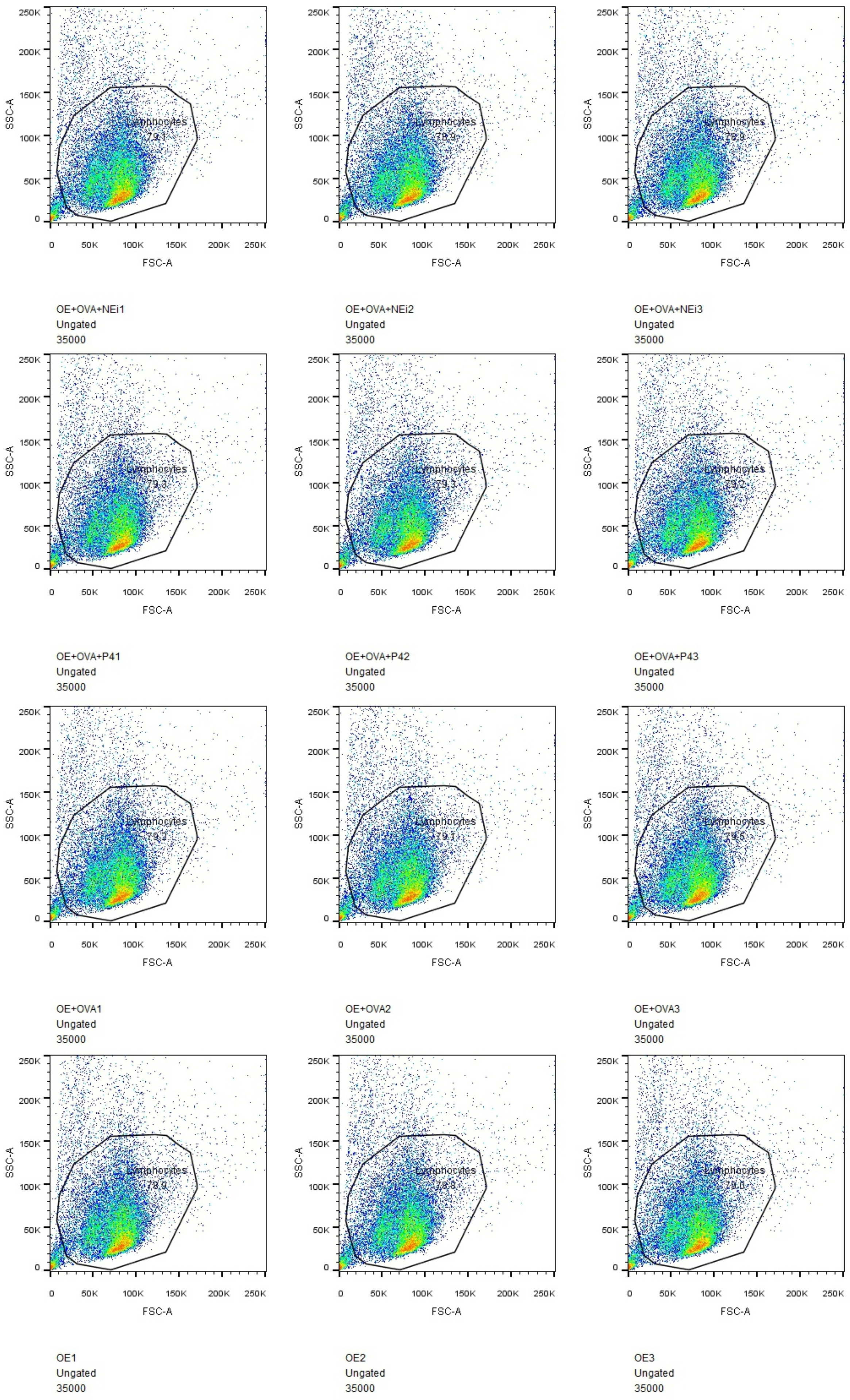

Figure 8. FL3 Gating CD45 for Figure 9

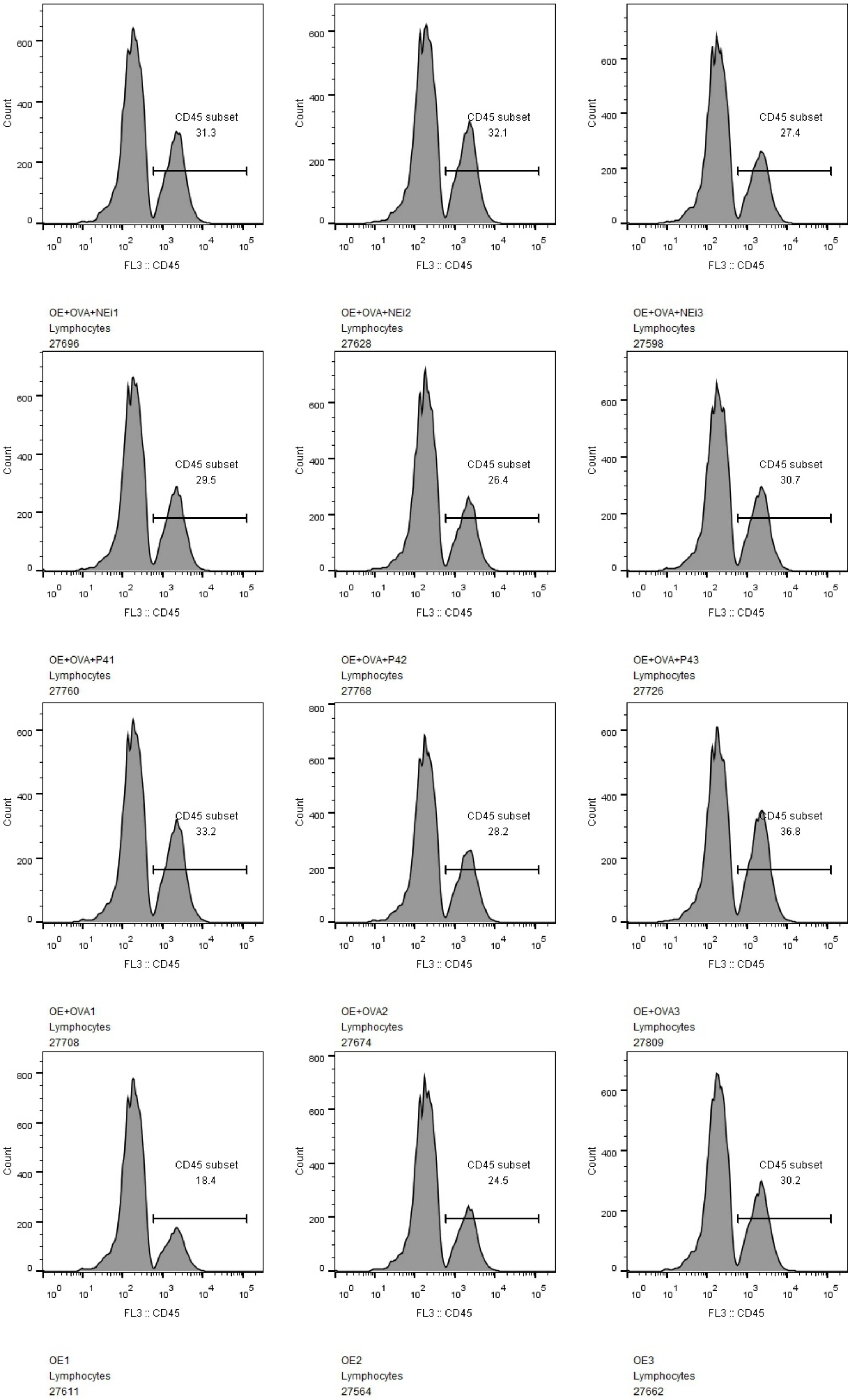

Figure 8. FL8 Gating for Figure 9

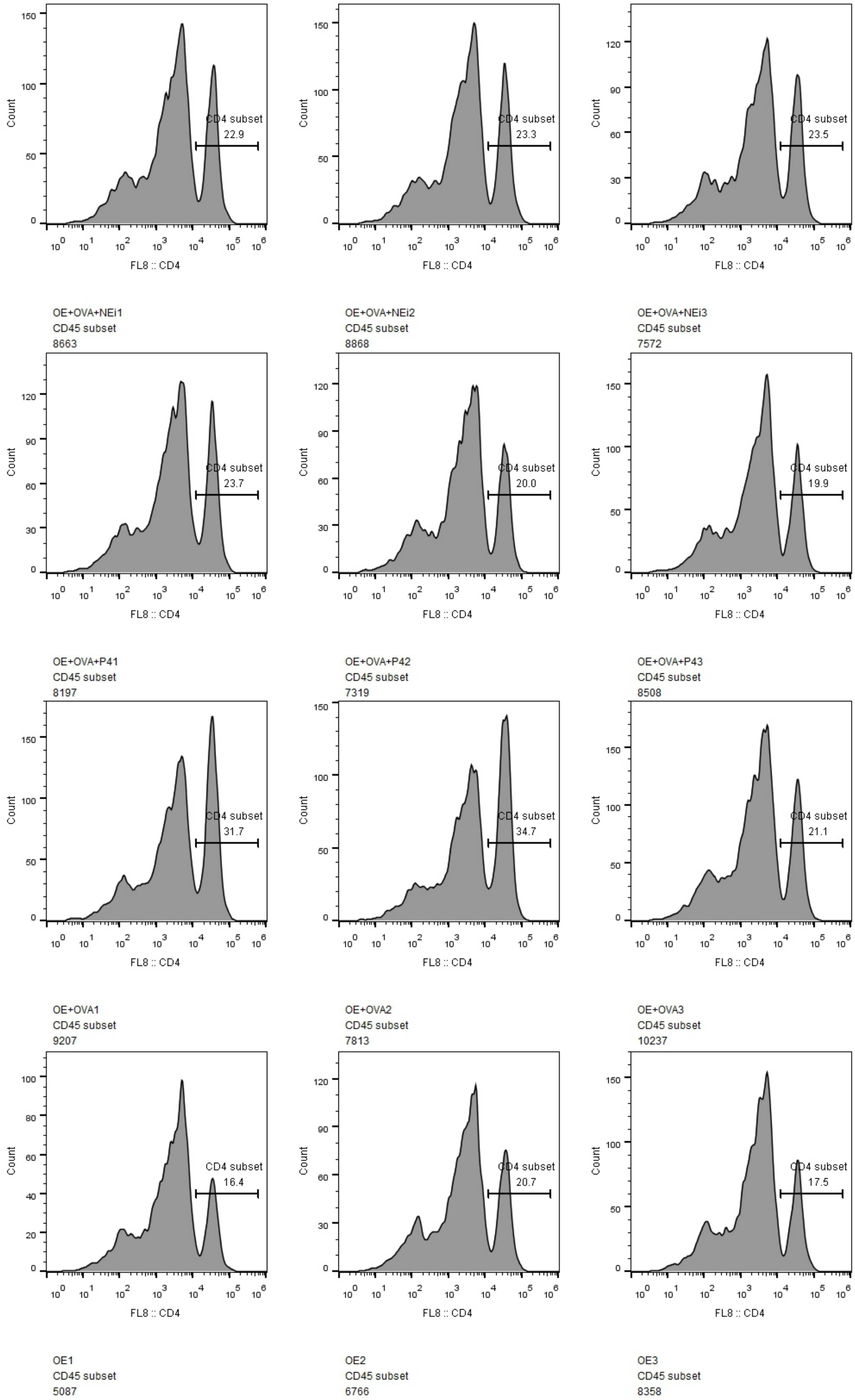

Figure 9A. CD45+CD4+CD44+ memory T cells

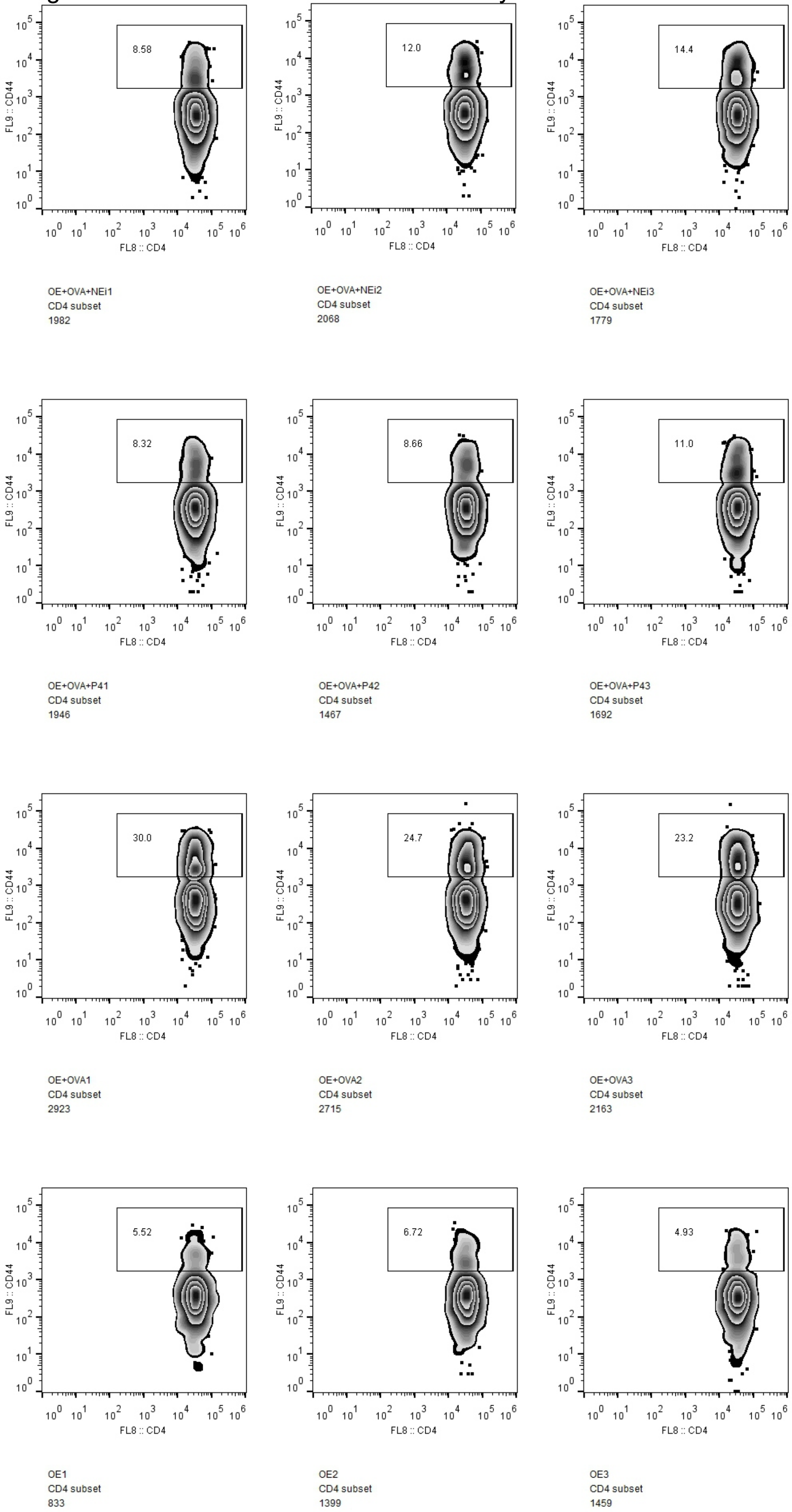

Figure 9B. CD45+CD4+IL-17 Th17 cells

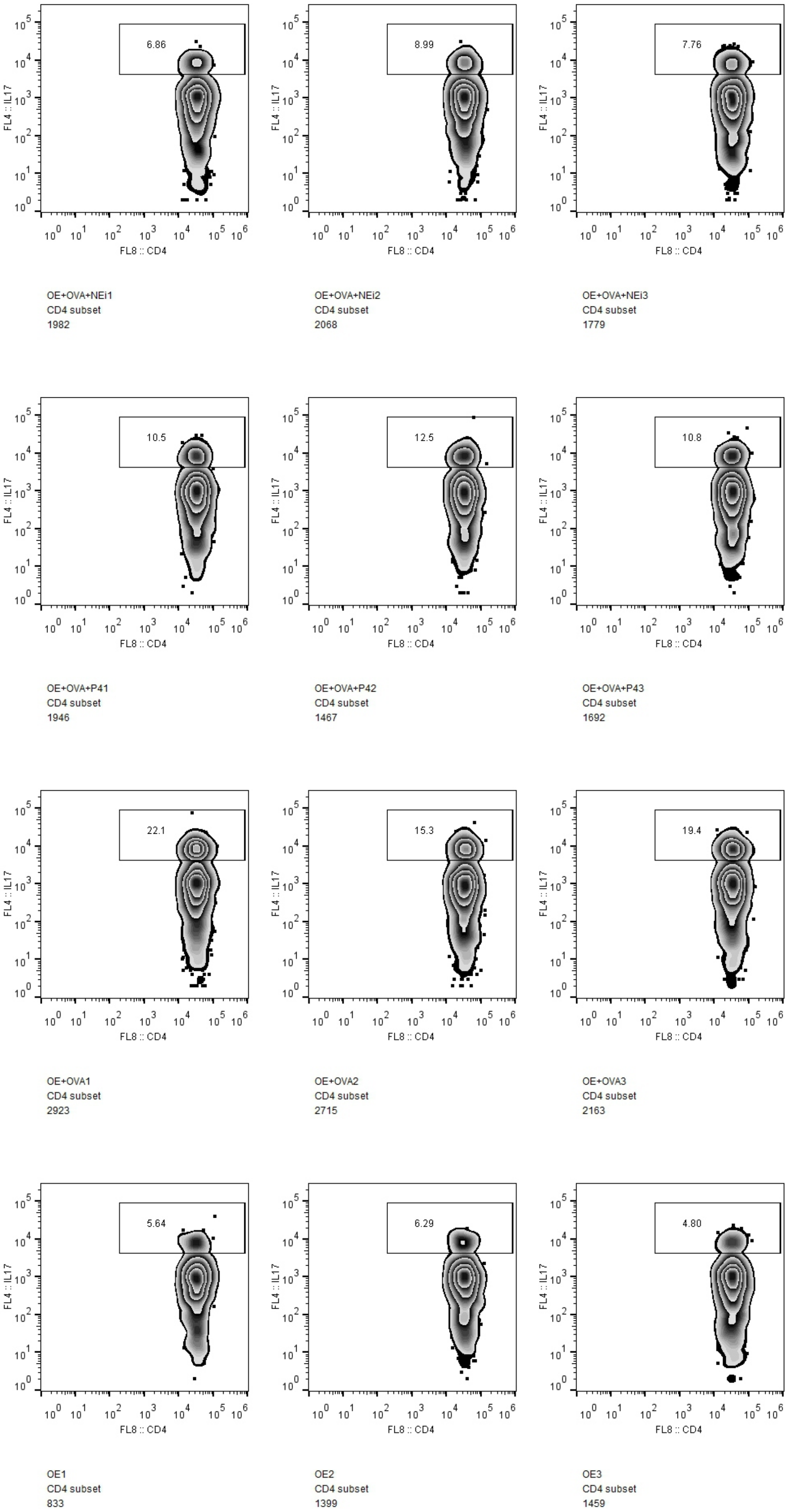

Figure 9C. CD45+CD4+CD25+Foxp3+ T cells

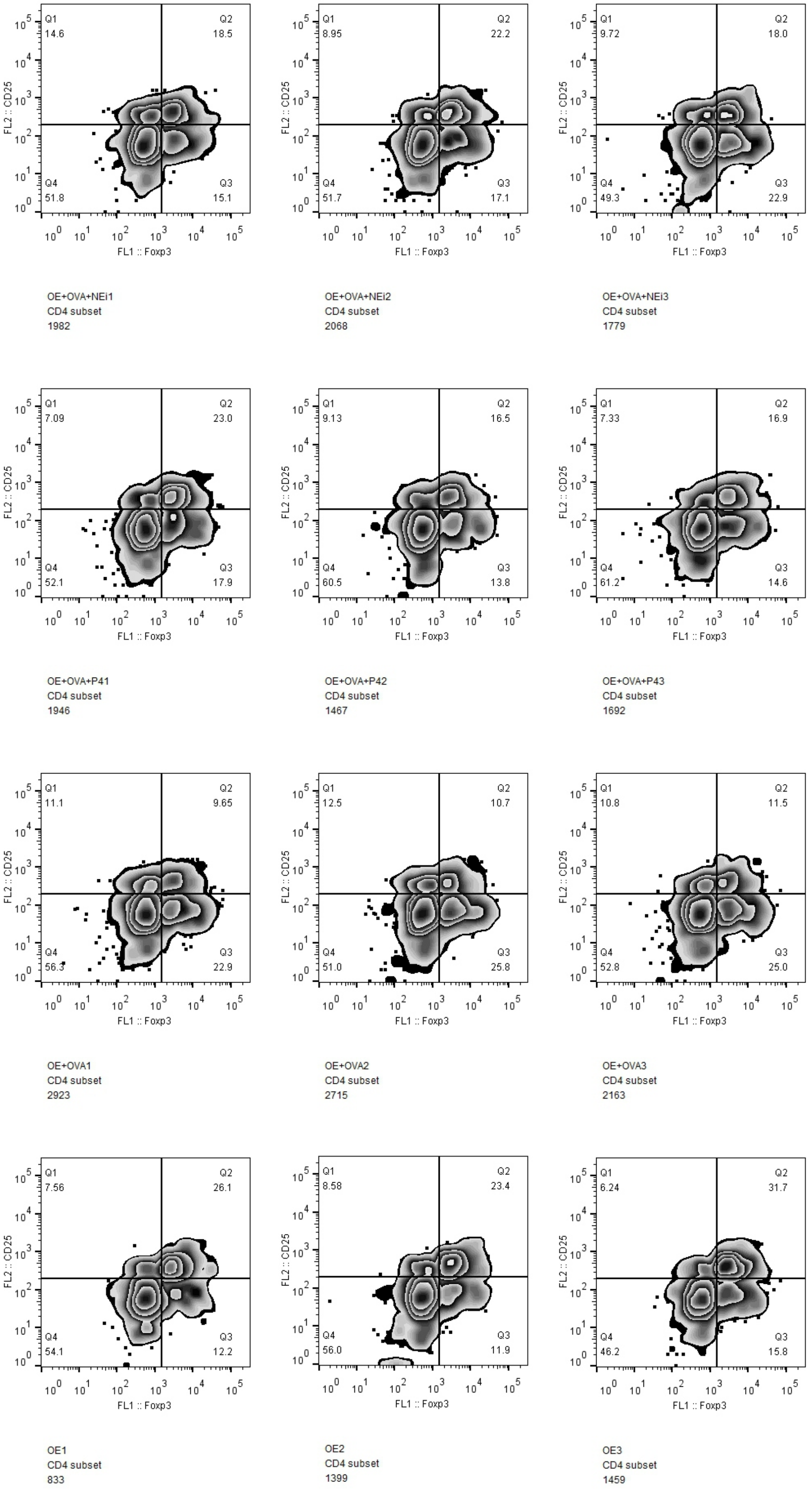

Gating strategy for Figure 10B, C, D

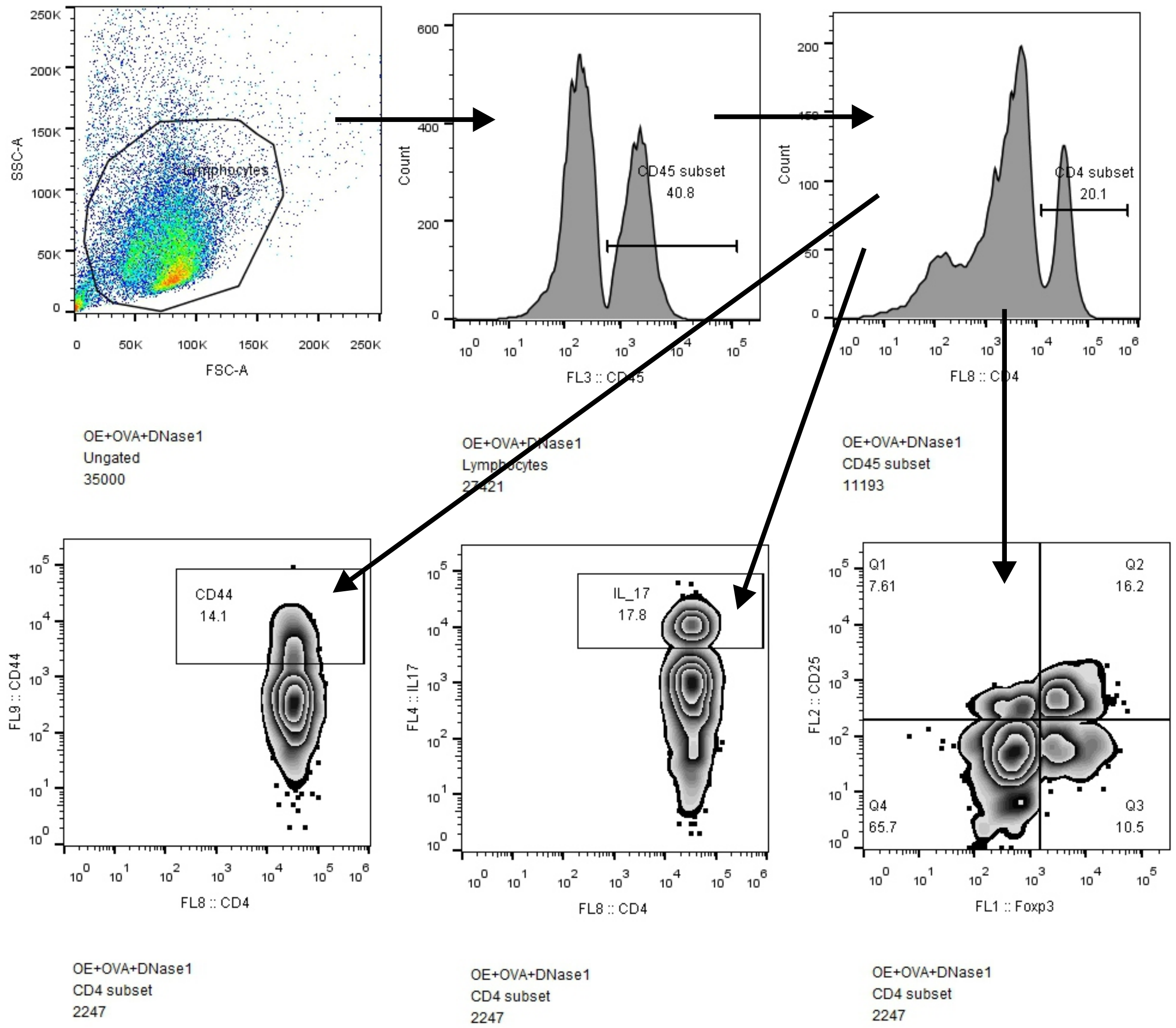

FSC/SSC Gating for Figure 10B, C, D

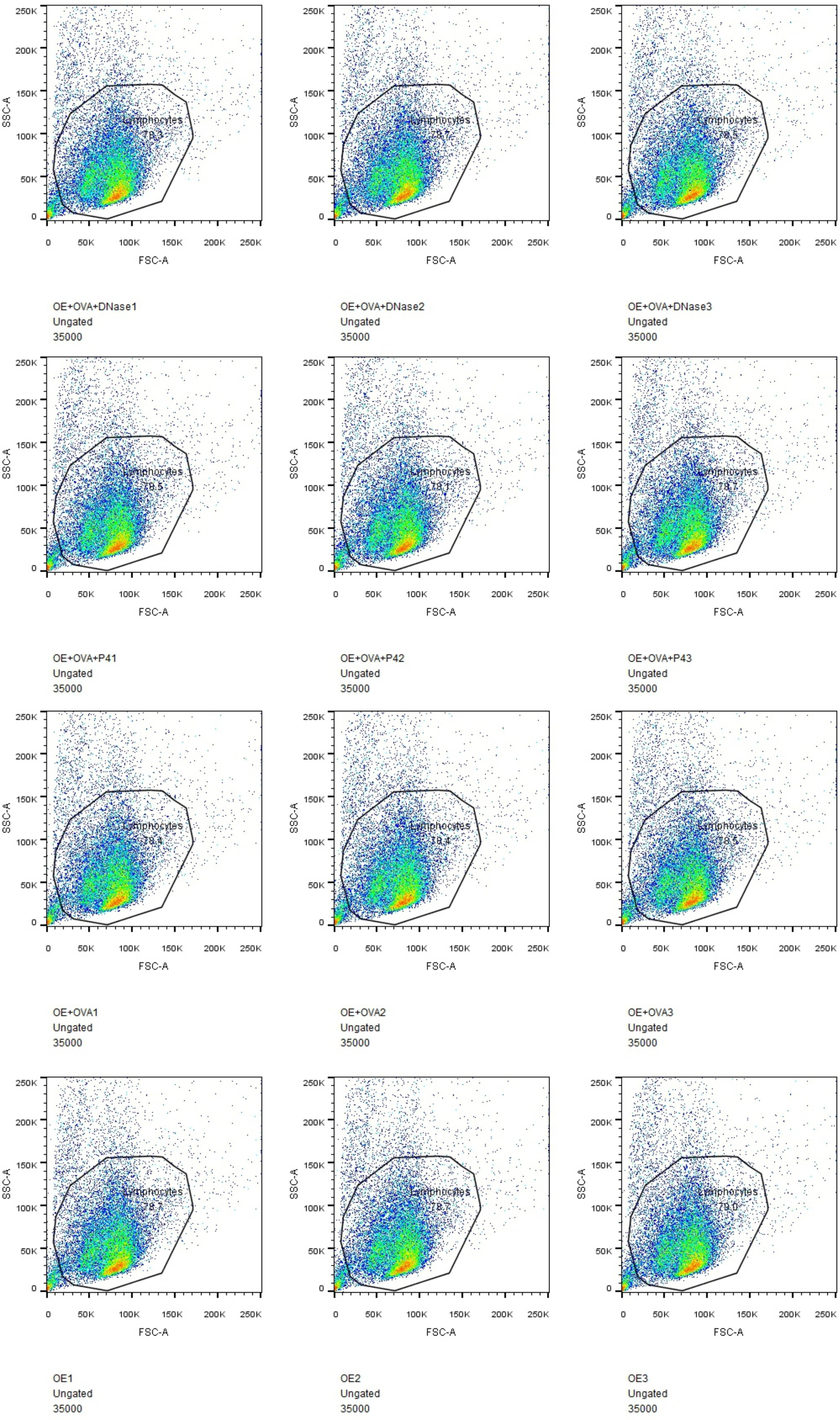

FL3 Gating CD45 for Figure 10B, C, D

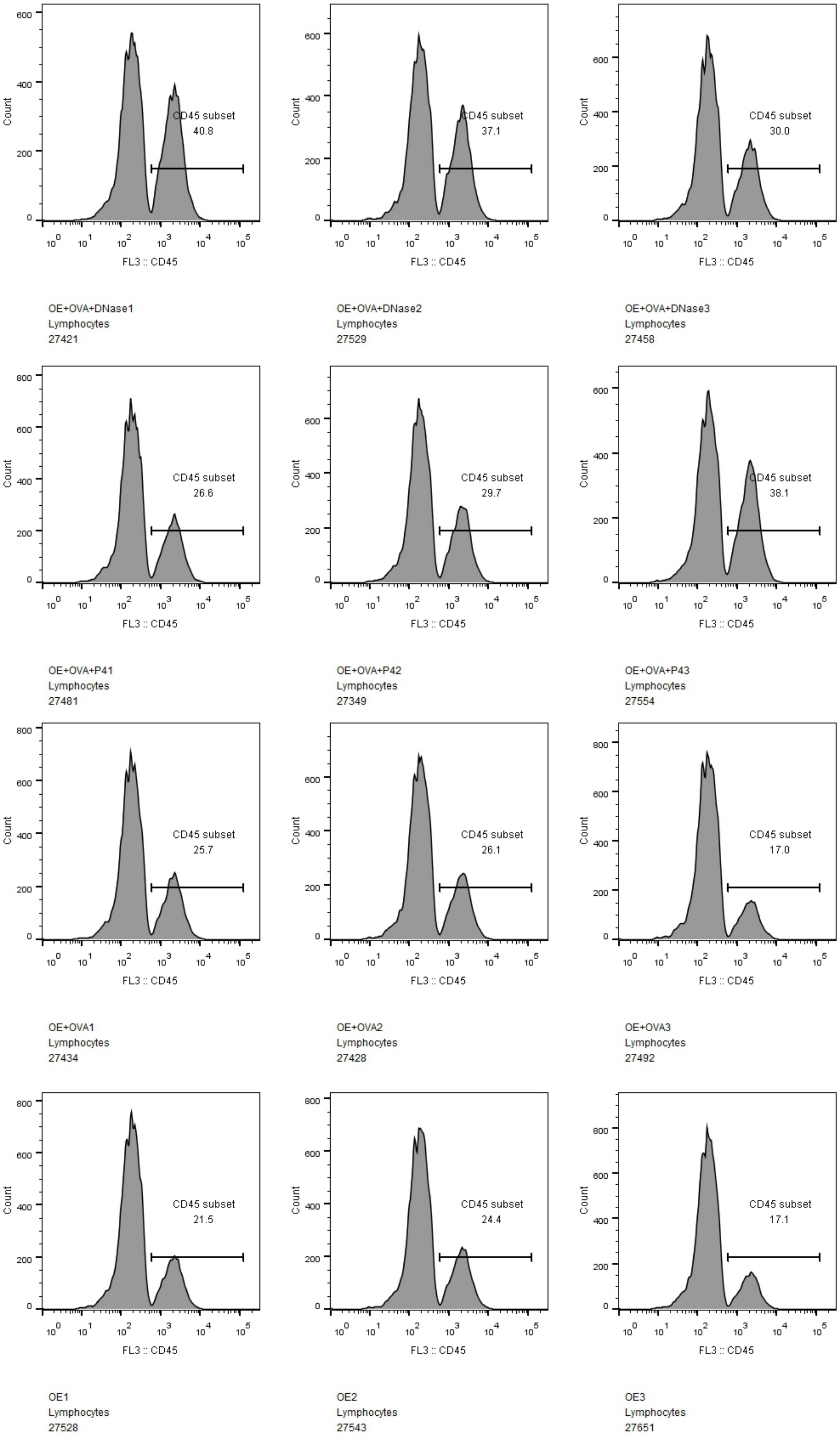

FL8 Gating CD4 for Figure 10B, C, D

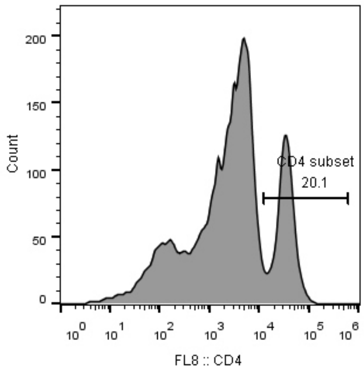

OE+OVA+DNase1
CD45 subset
11193

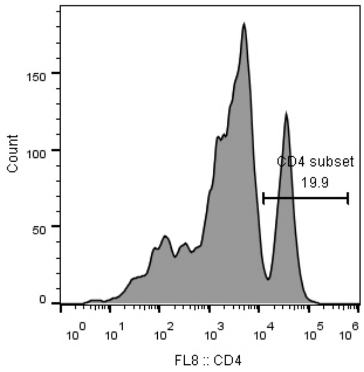

OE+OVA+DNase2
CD45 subset
10209

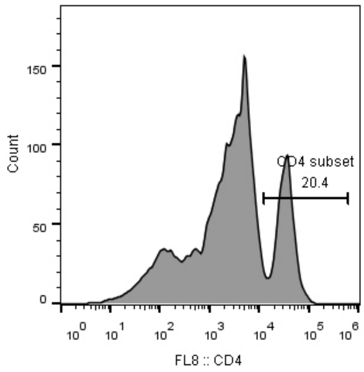

OE+OVA+DNase3
CD45 subset
8234

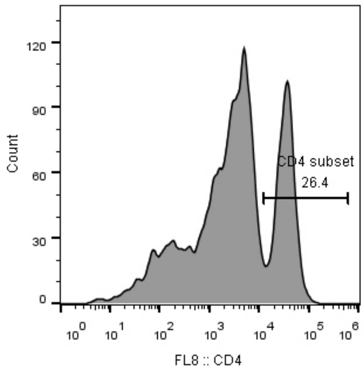

OE+OVA+P41
CD45 subset
7308

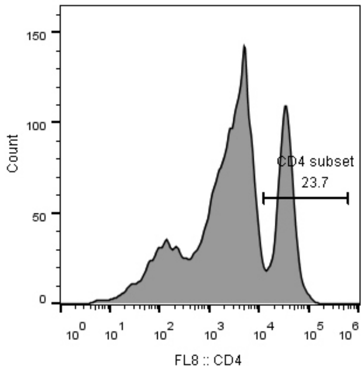

OE+OVA+P42
CD45 subset
8111

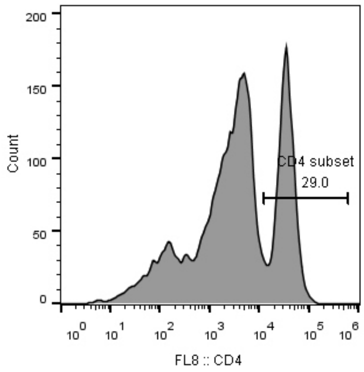

OE+OVA+P43
CD45 subset
10505

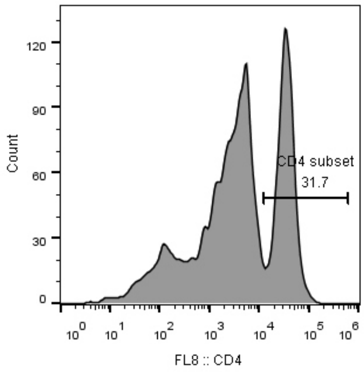

OE+OVA1
CD45 subset
7038

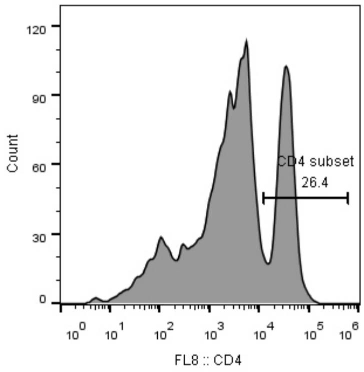

OE+OVA2
CD45 subset
7170

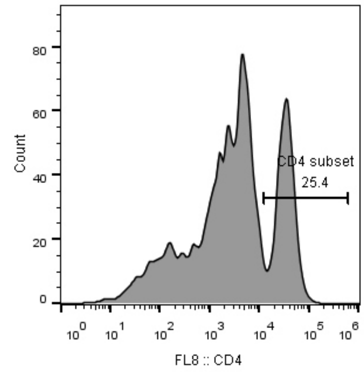

OE+OVA3
CD45 subset
4684

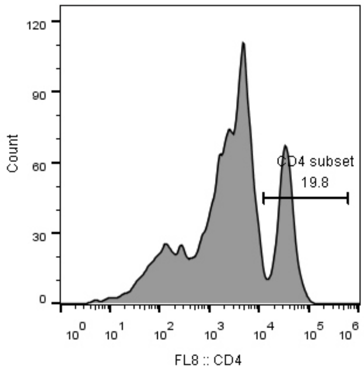

OE1
CD45 subset
5909

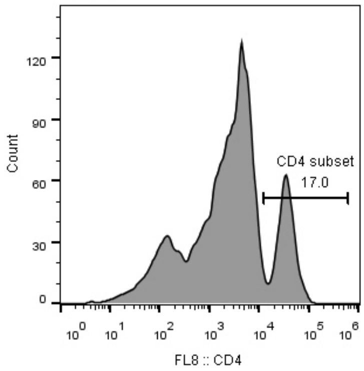

OE2
CD45 subset
6731

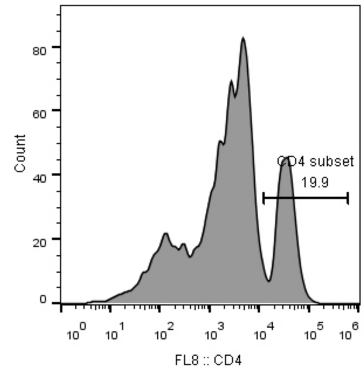

OE3
CD45 subset
4724

Figure 10B: CD45+CD4+CD44+

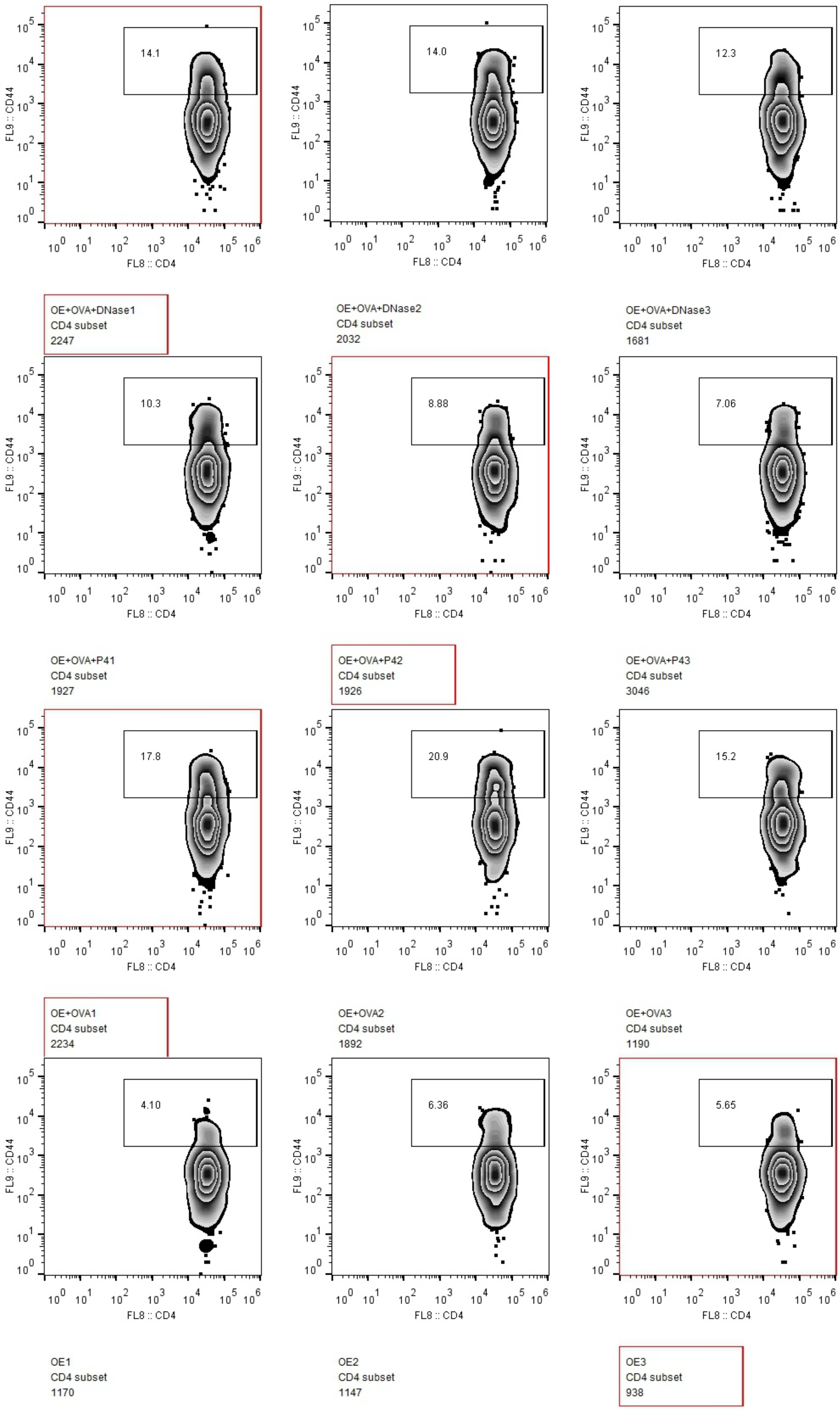

Figure 10C: CD45+CD4+IL17+

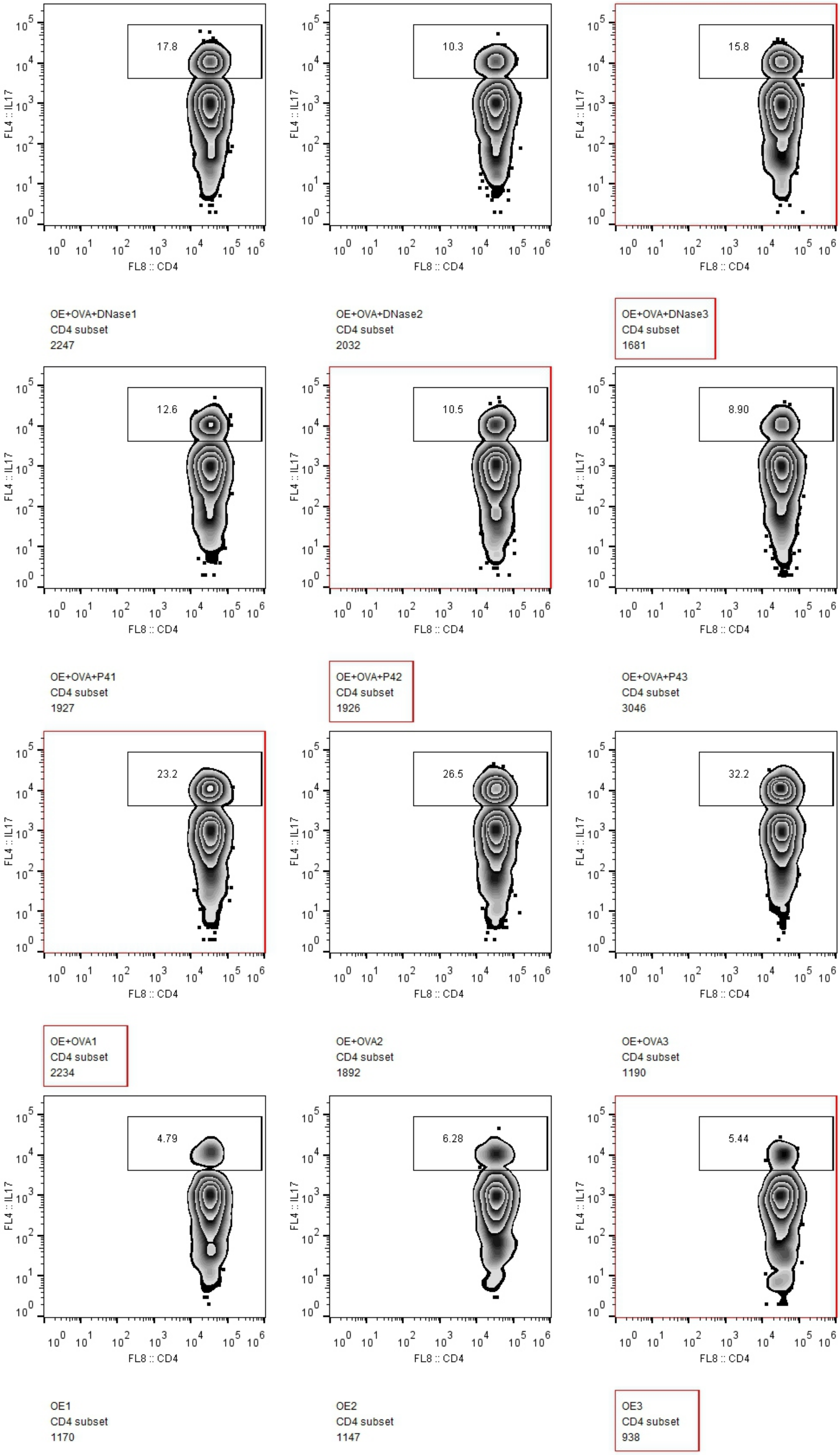

Figure 10D: CD45+CD4+Foxp3+CD25+

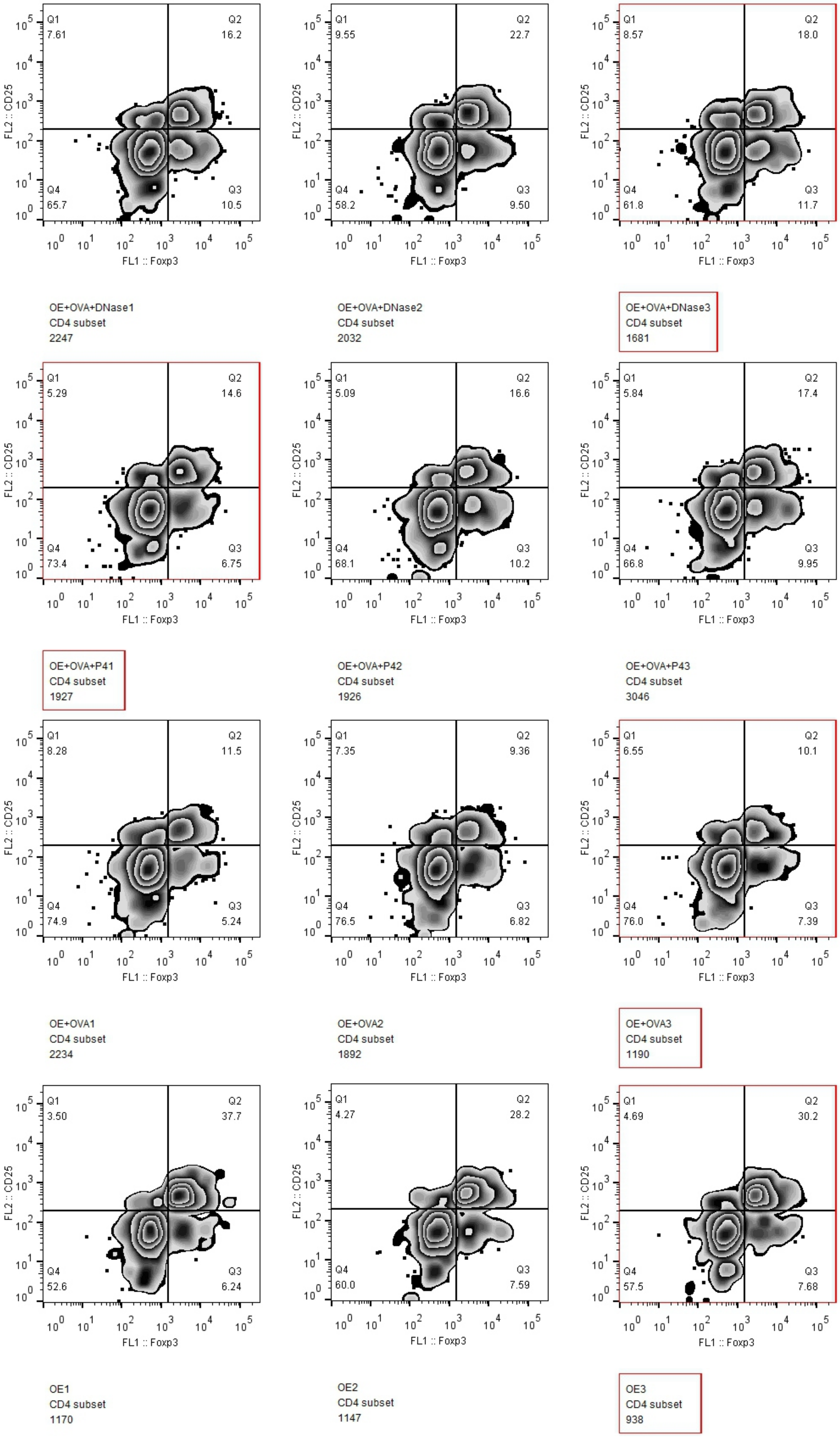

Supplement: Supplementary file 1 — Supplementary Information. [file 41598_2024_66439_MOESM1_ESM.pdf]
